# Online platform vs. doctors: a comparative exploration of congenital cataract patient education from virtual to reality

**DOI:** 10.3389/frai.2025.1548385

**Published:** 2025-06-03

**Authors:** Xuanqiao Lin, Lifang Bai, Xiaohuan Zhao, Lei Cai, Jin Yang

**Affiliations:** ^1^Department of Ophthalmology, Eye, Ear, Nose, and Throat Hospital of Fudan University, Shanghai, China; ^2^Key Laboratory of Myopia, Ministry of Health, Shanghai, China; ^3^Shanghai Key Laboratory of Visual Impairment and Restoration, Shanghai, China; ^4^Visual Rehabilitation Professional Committee, Chinese Association of Rehabilitation Medicine, Shanghai, China

**Keywords:** congenital cataracts, large language models, health education, public health care, artificial intelligence

## Abstract

**Objective:**

This study aimed to assess the quality and readability of patient education on congenital cataracts provided by Google, ChatGPT, and clinical doctors. Given the rarity of congenital cataracts and the need for accessible, accurate information for parents, we sought to evaluate the platforms’ effectiveness in delivering relevant health information.

**Methods and analysis:**

We developed two question banks related to congenital cataracts from different sources. Responses from Google, ChatGPT, and two doctors were evaluated across five criteria: correctness, completeness, readability, helpfulness, and safety. An ophthalmologist panel used a five-point Likert scale to score these responses. The readability of responses was also assessed using passage and readability statistics, with additional readability enhancements applied to ChatGPT responses.

**Results:**

The ChatGPT responses demonstrated similar quality to those from experienced doctors, particularly excelling in readability, which was enhanced further with simplification techniques. Resident doctors provided the most readable doctor responses, while Google results scored the lowest across all five evaluative criteria. Post-enhancement, ChatGPT responses showed significant improvements in readability and maintained response quality.

**Conclusion:**

ChatGPT is a promising tool for delivering accessible, accurate information on congenital cataracts, especially for populations with lower health literacy. This study underscores the value of AI in healthcare education for rare conditions and highlights the need for consulting multiple information sources for comprehensive health guidance. ChatGPT, with readability enhancements, stands out as a particularly effective resource for public health information on congenital cataracts.

## 1 Introduction

In recent years, with the advancement of network technology, there has been a growing trend of individuals and families seeking health advice or disease information through online channels and digital platforms (Bujnowska-Fedak and Węgierek, 2020; Wangler and Jansky, 2023). Digital platforms offer the advantages of fast search speeds, a wide variety of information, and easy accessibility from any device. In this context, Google, as the search engine of choice for the general public, has gained significant popularity and is widely utilized by patients with chronic conditions seeking online medical assistance (Lee et al., 2015). However, existing studies in the field of ophthalmology have shown that the content often surfaces in search results for common eye conditions across various specialties, such as retinal diseases, glaucoma, and oculoplastic surgery, frequently misleading and presented at a complexity level that exceeds the understanding of the average layperson (Cohen et al., 2023a; Cohen and Pershing, 2022; Cohen et al., 2023b).

An increasingly popular source of information among patients seeking to learn more about their health is artificial intelligence (AI) large language models (LLMs) (Dave et al., 2023). ChatGPT (Open AI) stands out as the most popular LLM in the United States, with its user base growing to nearly 200 million over the past year (AL-Smadi, 2023). ChatGPT has shown utility in the medical field, assisting in the creation of operative notes and discharge summaries (Brown et al., 2020; Singh et al., 2023). However, in the field of ophthalmology, the effectiveness of ChatGPT in answering patient questions remains uncertain (Bernstein et al., 2023; Potapenko et al., 2023).

Congenital cataract, a rare eye disease, stands as one of the major causes of visual loss in children globally (Sheeladevi et al., 2016). Late diagnosis and treatment can lead to irreversible deprivation amblyopia and permanent severe visual impairment or blindness (Bremond-Gignac et al., 2020; Lenhart and Lambert, 2022). Unfortunately, parents often find themselves with limited avenues to seek information and tend to rely on doctors and online platforms for guidance. While there has been extensive research on the responses to patient questions in several ophthalmology subspecialties, including retina and cataract, on common online platforms and AI systems like ChatGPT, there is a significant gap when it comes to information access for rare congenital eye diseases (Potapenko et al., 2023; Momenaei et al., 2023; Cohen et al., 2024). Moreover, they require a reliable channel to inquire about the surgery and postoperative visual rehabilitation for congenital cataracts to make informed decisions regarding their children’s eye care. However, to date, there has been no comprehensive comparison of different characteristics of medical responses obtained by patients with congenital cataracts through various online methods and different doctors.

**Figure 1:**
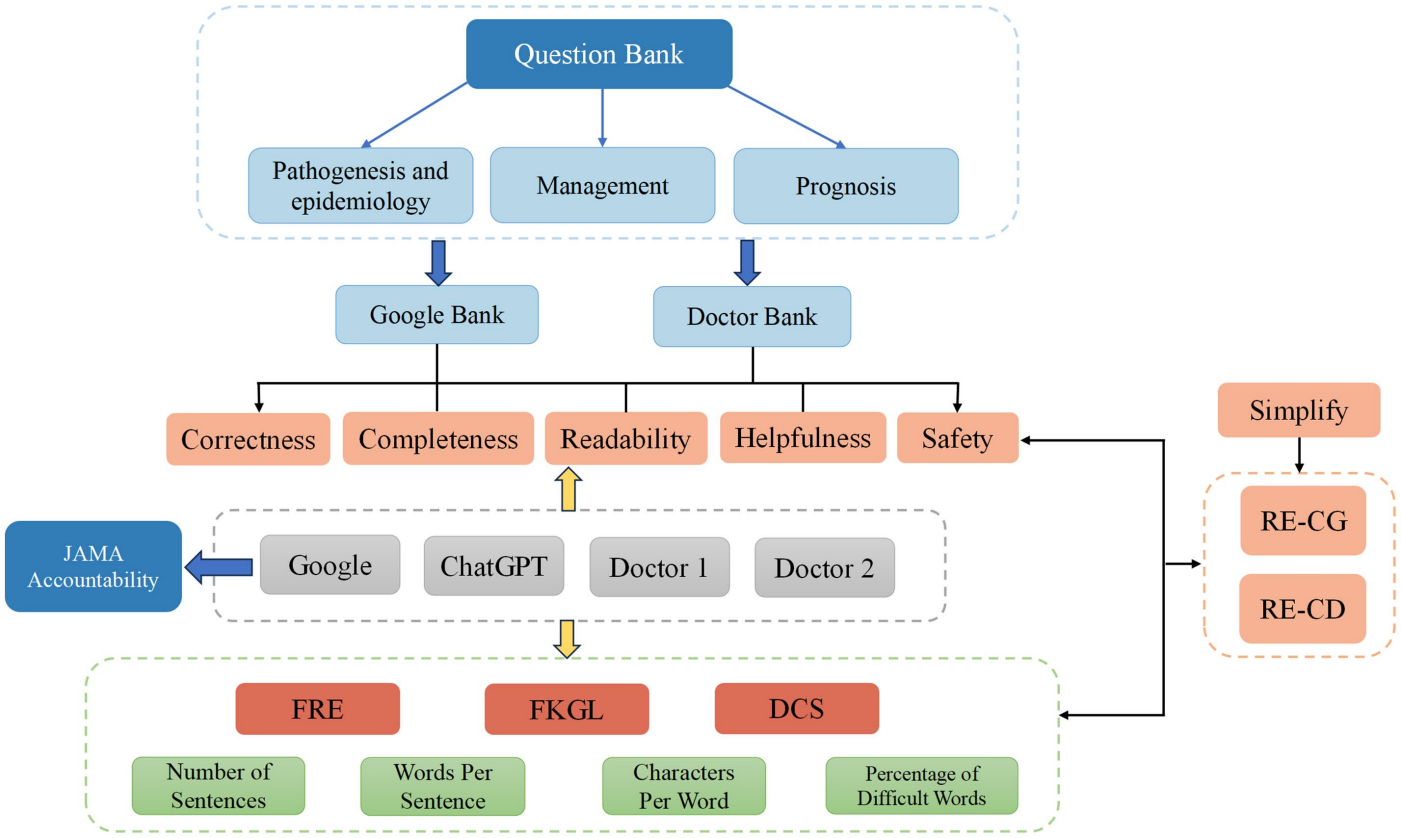
Flowchart of this study. FRE, Flesch Reading Ease; FKGL, Flesch–Kincaid Grade Level; DCS, Dale-Chall Score.

The objectives of our study are threefold. Firstly, we aim to identify the most common questions about congenital cataracts that patients inquire about online and those summarized by clinical doctors. Secondly, a panel of ophthalmologists will assess the characteristics and readability of responses to these questions on three different platforms: Google, ChatGPT, and clinical doctors. Lastly, we further simplified the responses to questions using LLM to make them more aligned with the reading abilities of the general public and compared the characteristics of the simplified responses (Figure 1).

## 2 Materials and methods

### 2.1 Question banks

To generate a representative set of frequently asked questions (FAQs) related to congenital cataracts, we curated two distinct question banks. Each question was categorized as “Pathogenesis and Epidemiology,” “Management,” or “Prognosis.”

#### 2.1.1 Google Bank

A clean-install Google Chrome browser (incognito mode, location and ad filters disabled) was used to search for the term “congenital cataract.” We collected the top 10–15 auto-populated queries under the “People Also Ask” (PAA) section. The process involved recursively expanding each listed question by clicking to reveal additional FAQs, and repeating this step until saturation (i.e., no new unique questions appeared). We then deduplicated and refined the final list through manual review by two ophthalmologists to ensure clinical relevance and linguistic clarity.

#### 2.1.2 Doctor Bank

This set was developed independently by two senior pediatric ophthalmologists, who were asked to provide 15 of the most common questions they encounter from patients and caregivers regarding congenital cataracts and related management. The lists were then consolidated and reviewed to remove overlap and ensure diversity in question types.

### 2.2 Questions queries

For each FAQ within the “Google Bank” and “Doctor Bank,” we conducted a search in Google’s search bar and recorded the first reasonable answer we found.

Since most patients are unlikely to pay solely for medical consultations, we opted to use ChatGPT 4o mini instead of more advanced version. ChatGPT 4o mini (Version: August 15, 2024) was queried with 30 FAQs from two question banks related to “congenital cataract,” and its responses were carefully recorded. While we recognize that LLMs may generate varied responses across sessions, in this study we focused on evaluating a single representative output per question, consistent with real-world use where a patient receives one answer at a time.

We also presented the 30 FAQs from both the Google and Doctor Question Banks to an experienced cataract surgeon (X.Z., Doctor 1) and a senior ophthalmology resident (L.B., Doctor 2), neither of whom had been involved in the creation of the question banks. After making minor grammatical adjustments and modifications that did not affect the essence of the responses, two surgeons’ answers were recorded. The responses to 15 FAQs from two question banks were provided by Google, ChatGPT, and two doctors, resulting in eight groups: (1) Google-Google (GG); (2) Google-Doctor (GD); (3) ChatGPT-Google (CG); (4) ChatGPT-Doctor (CD); (5) Doctor 1-Google (DG 1); (6) Doctor 1-Doctor (DD 1); (7) Doctor 2-Google (DG 2); (8) Doctor 2-Doctor (DD 2) (see Supplementary Table S1).

### 2.3 Passage and readability statistics

The responses to the 30 FAQs in this study were evaluated for readability using four passage statistic methods and three established assessment tools available on the Readability Analyzer platform^1^: Number of Sentences, Words Per Sentence, Characters Per Word, Percentage of Difficult Words, Flesch Reading Ease (FRE), Flesch–Kincaid Grade Level (FKGL), and Dale-Chall Score (DCS) (Cohen et al., 2024; Wu et al., 2023). FRE is calculated as: 206.835–1.015 × average sentence length—84.6 × average syllables per word. Scores range from 0 to 100, with higher values indicating easier text. A FRE score > 60 is generally considered acceptable for patient education. FKGL translates reading difficulty into U.S. school grade levels (e.g., 8.0 indicates 8th grade). Scores ≤ 8 are preferred for lay comprehension. DCS considers both sentence length and the percentage of unfamiliar (non–common) words, using the Dale–Chall 3,000-word list as a reference. Scores below 7.5 indicate appropriate readability for most health materials (see Supplementary Table S2).

### 2.4 JAMA accountability analysis

The accountability of the 30 Google-listed websites was assessed using a 0–4 scale based on the Journal of the American Medical Association (JAMA) benchmarks. According to JAMA guidelines, a trustworthy educational site must: (1) Include all authors and their relevant credentials, (2) List references, (3) Provide disclosures and (4) Provide date of last update (Silberg et al., 1997).

### 2.5 Expert panel evaluation

Three chief ophthalmologists (J.Y., L.C., and Y.C.) independently evaluated the responses generated by different platforms using five predefined criteria: “Correctness” (evaluating the medical correctness of the reply), “Completeness” (measuring the extent to which the response covers all necessary aspects of the query), “Readability” (assessing ease of understanding for the patient), “Utility” (evaluating the usefulness in aiding patient comprehension and management), and “Safety” (assessing the potential of the response to guide patients safely, ensuring the information provided does not mislead or pose any risk to patient health). Responses were measured using a 5-point Likert scale, where a rating of 1 signified strong disagreement, 2 indicated disagreement, 3 represented a neutral stance, 4 denoted agreement, and 5 conveyed strong agreement. Each rating was separated by a 48-h washout period (see Supplementary Table S3).

### 2.6 Enhancing the readability of ChatGPT responses

To improve the readability of responses from the LLM, we provided ChatGPT 4o mini (Version: October 10, 2024) with the following instruction: “Given that patient education materials are recommended to be written at a sixth-grade reading level, can you rewrite the following text to a simpler reading level: (insert text).” Record these responses (RE-CG and RE-CD, respectively) and conduct the same passage statistics, readability statistics, and scoring as before, then compare it with the original, unsimplified response (see Supplementary Tables S2, S3).

### 2.7 Statistical analysis

The statistical analysis was conducted using IBM SPSS, version 25.0. The Shapiro–Wilk test was used to assess the normality of the results. The overall readability and response characteristics of the two question banks were compared using paired *t* test the Mann–Whitney *U* test. Analysis of variance (ANOVA) or the Kruskal-Wallis test was used to compare differences among responses within each question bank. Pairwise comparisons between groups were conducted using the Bonferroni test or Dunn’s test. The statistical significance level for all tests was set at *p* < 0.05, and Bonferroni correction was applied to adjust the results of the multiple analyses.

## 3 Result

### 3.1 Question banks and classifications

Supplementary Table S1 presents the two question banks and their classifications. In both question banks, there are open-ended questions (e.g., What are the long-term effects of congenital cataracts?) as well as closed-ended questions (e.g., What are congenital cataracts?). Additionally, the Doctor Bank includes more practical questions (e.g., How long after congenital cataract surgery should the child avoid physical education classes?). Compared to the Google Bank, the Doctor Bank contains a higher proportion of questions classified as “Management” and “Prognosis” (*n* = 7, 46.67% vs. *n* = 14, 93.33%).

### 3.2 Comparison between the two question banks

**Figure 2:**
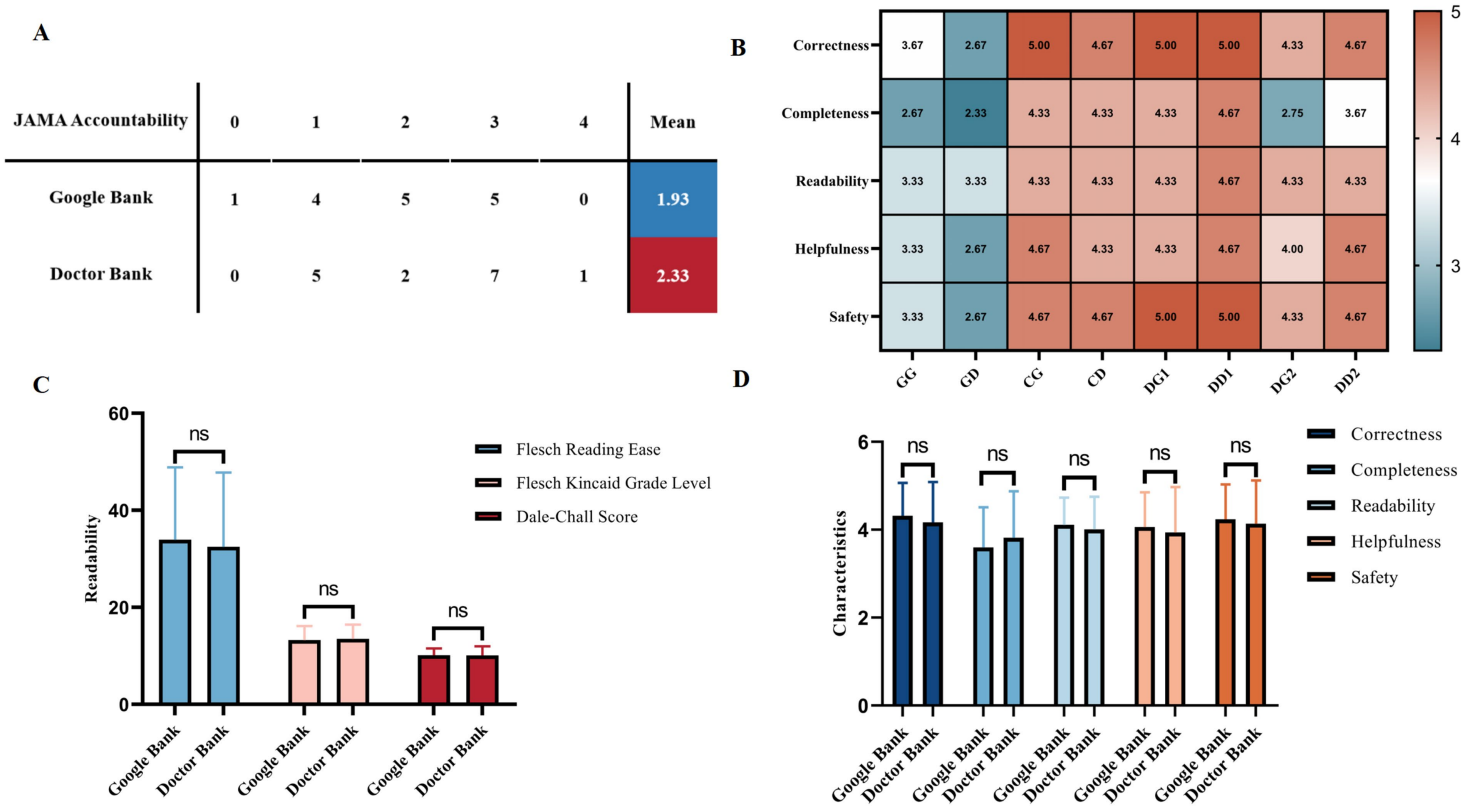
Comparison between the two question banks. **(A)** JAMA accountability of Google responses. **(B)** Heat-map for response characteristics of different groups. **(C)** Readability of different question banks. **(D)** Response characteristics of different question banks. Statistical significance is denoted as follows: ns = not significant (*p* ≥ 0.05); *p < 0.05; **p < 0.01; ***p < 0.001.

Figure 2 presents a comprehensive comparison between the two question banks, including JAMA accountability of the sources of Google answers (Figure 2A). The average JAMA accountability score for the 15 analyzed webpages was 1.93 for the Google Bank and 2.33 for the Doctor Bank, with a maximum possible score of 4. It also features the response characteristics for both question banks across different methods (Figure 2B), and the bar charts illustrate the comparison of readability statistics and response characteristics for the two banks (Figures 2C,D). It can be observed that there are no statistically significant differences between the two question banks in terms of either readability or response characteristics (Mann–Whitney *U* Test, *p* > 0.05).

Table 1 presents the passage statistics results for the initial eight groups, showing statistically significant differences across the four parameters (one-sample *t*-test or Wilcoxon test, *p* < 0.05). It was found that the two ChatGPT groups had higher values for Number of Sentences, Characters Per Word, and Percentage of Difficult Words compared to the other groups, while Words Per Sentence was the lowest among the four responders.

**Table 1:** Passage statistic of eight responser for two question banks.

Passage statistics	GG	GD	CG	CD	DG1	DD1	DG2	DD2	*p* value
Number of Sentences	3.20	4.40	17.80	19.53	5.20	11.40	2.80	3.13	< 0.05^*^
Words Per Sentence	18.05	19.64	16.24	16.84	19.24	18.74	22.28	21.17	< 0.05^**^
Characters Per Word	5.25	5.35	5.52	5.56	5.54	5.41	4.75	4.97	< 0.05^**^
Percentage of Difficult Words	26.49%	27.23%	27.06%	29.23%	27.15%	27.46%	16.46%	18.94%	< 0.05^*^

*One-sample *t*-test.

**One-sample Wilcoxon test.

### 3.3 Intra-group comparison between the two question banks

**Figure 3:**
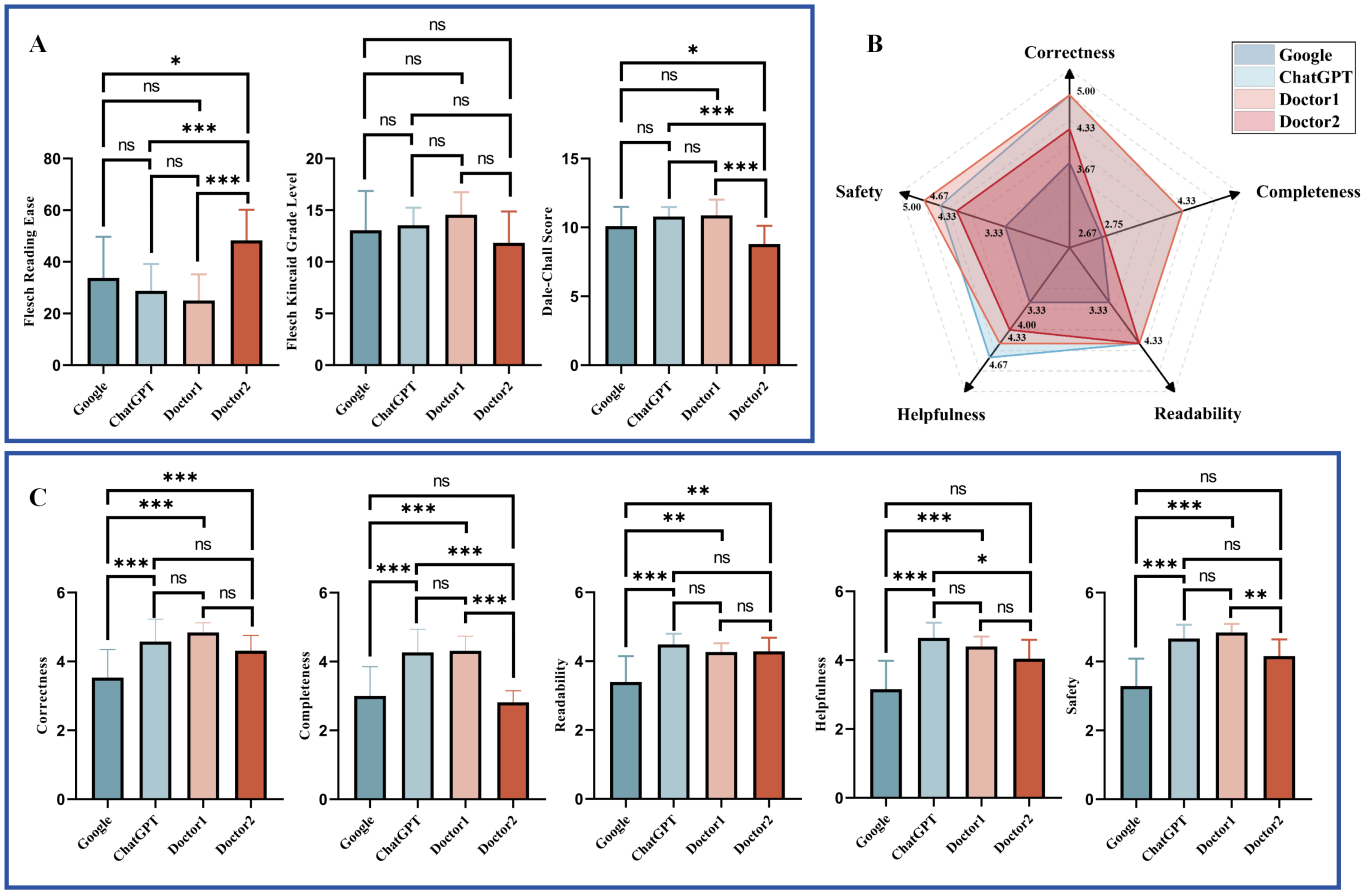
Comparative readability and characteristics of responses to Google Bank questions. **(A)**. Readability of different respondents to Google Bank. **(B)** Radar plot of responses to Google Bank from different responders. **(C)** Response characteristics of different respondents to Google Bank. Statistical significance is denoted as follows: ns = not significant (*p* ≥ 0.05); *p < 0.05; **p < 0.01; ***p < 0.001.

**Figure 4:**
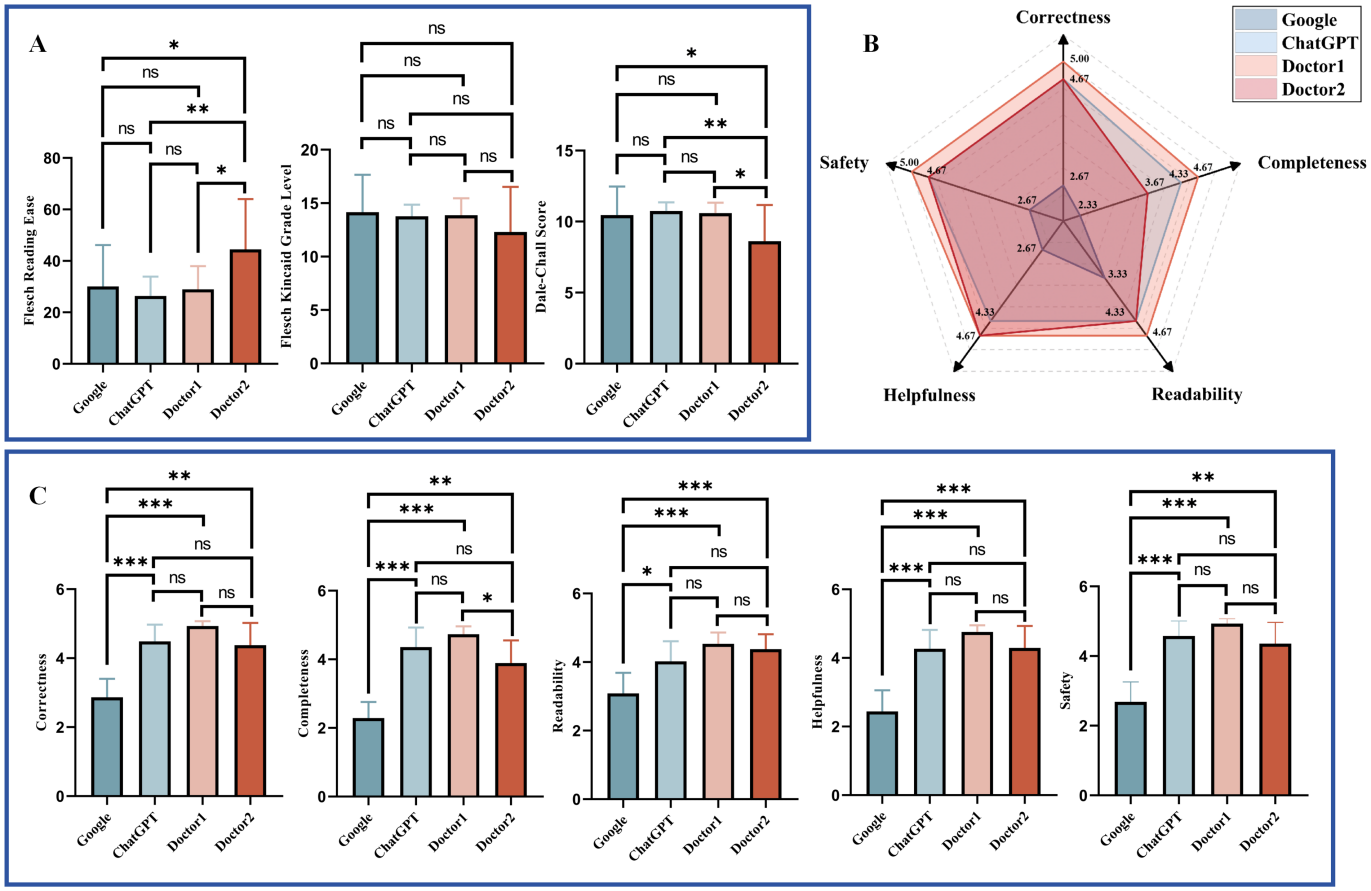
Comparative readability and characteristics of responses to Doctor Bank questions. **(A)** Readability of different respondents to Doctor Bank. **(B)** Radar plot of responses to Doctor Bank from different responders. **(C)** Response characteristics of different respondents to Doctor Bank. Statistical significance is denoted as follows: ns = not significant (*p* ≥ 0.05); *p < 0.05; **p < 0.01; ***p < 0.001.

The readability and response characteristics of answers from Google, ChatGPT, Doctor 1, and Doctor 2 were analyzed for two question banks: Google (Figure 3) and Doctor (Figure 4). For the readability analysis, Doctor 2 demonstrated significantly higher FRE scores compared to other doctors in both question banks (Bonferroni test, *p* < 0.05). However, the differences in FKGL were not significant across the groups in either bank (Bonferroni test, *p* > 0.05 for two Banks). DCS showed that Doctor 2 consistently had easier readability than the other groups (Bonferroni test, *p* < 0.05) in both question banks.

Response characteristics were visualized using radar charts (Figures 3B, 4B). Across both question banks, Doctor 1 achieved the highest scores in Correctness, Completeness, Readability, and Safety (5.00, 4.33, 4.33, 5.00 for Google Bank and 5.00, 4.67, 4.67, 5.00 for Doctor Bank, respectively). For Helpfulness, ChatGPT ranked the highest in the Google Bank (4.67), while Doctor 1 & 2 scored the highest in the Doctor Bank (4.67). Conversely, Google received the lowest scores across all five response characteristics.

The detailed statistical comparison (Figures 3C, 4C) revealed that in both question banks, Google had significantly lower scores in Correctness compared to other responders (Dunn’s test, *p* < 0.05). For Completeness, Google scored lower than the other groups in both banks, except for Doctor 2 in the Google Bank (Dunn’s test, adjusted *p* > 0.05). In the Doctor Bank, Google’s scores for the remaining three characteristics were also significantly lower than those of the other responders (Dunn’s test, *p* < 0.05). In the Google Bank, Google had significantly lower Readability scores (Dunn’s test, *p* < 0.05). In addition, ChatGPT and Doctor 1 scored higher in Helpfulness and Safety.

### 3.4 Analysis of responses after readability enhancement

Table 2 shows that both the RE-CG and RE-CD groups have fewer Words Per Sentence and significantly reduce Characters Per Word and Percentage of Difficult Words compared to their original groups after readability enhancement (Paired *t*-test or Mann–Whitney *U* test, *p* < 0.05). However, the Number of Sentences increased slightly, though not significantly.

**Table 2:** Passage statistic of responses after readability enhancement.

Passage statistics	CG	RE-CG	*P* Value	CD	RE-CD	*P* Value
Number of Sentences	17.80	20.33	0.423^*^	19.53	20.73	0.609^*^
Words Per Sentence	16.24	11.54	< 0.05^*^	16.84	15.36	0.079^*^
Characters Per Word	5.52	4.79	< 0.05^**^	5.56	4.72	< 0.05^**^
Percentage of Difficult Words	27.06%	13.92%	< 0.05^**^	29.23%	14.27%	< 0.05^*^

*Paired *t*-test.

**Mann–Whitney *U* Test.

**Figure 5:**
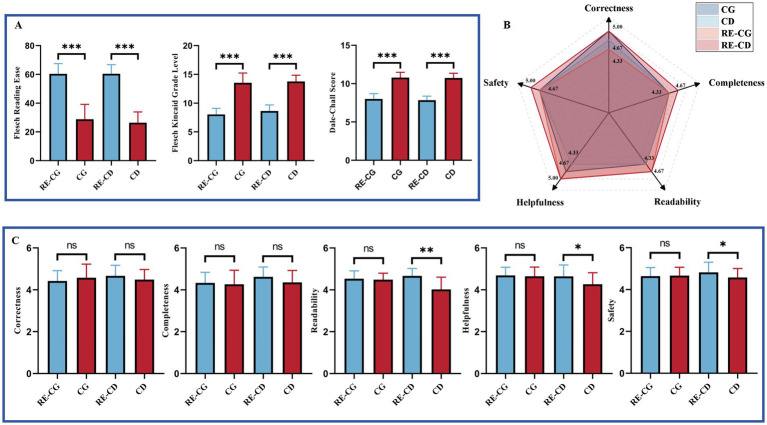
Comparative readability and characteristics of responses before and after readability enhancement. **(A)** Readability of responses before and after readability enhancement. **(B)** Radar plot of responses before and after readability enhancement. **(C)** Response characteristics before and after readability enhancement. Statistical significance is denoted as follows: ns = not significant (*p* ≥ 0.05); *p < 0.05; **p < 0.01; ***p < 0.001.

Figure 5 shows a comparison of ChatGPT’s readability and response characteristics across two question banks before and after applying readability enhancement instructions. In both banks, the FRE scores of RE groups were higher than those of the original group, while the other two readability metrics were significantly lower (Mann–Whitney *U* Test, *p* < 0.001) (Figure 5A). As for the response characteristics after the readability improvement, all RE groups scored higher than the original groups, except for a decrease in Correctness in the RE-CG group. Moreover, in the RE-CD group, Readability, Helpfulness, and Safety indicated significant improvement (Mann–Whitney *U* Test, *p* < 0.05) (Figures 5B,C).

## 4 Discussion

Several recent studies have adopted similar methodologies—using FAQs, comparing responses from ChatGPT and other platforms, and evaluating them with expert panels—in various medical fields such as dermatology and cardiology (Montag et al., 2025; Chen et al., 2025; Karnan et al., 2025). To the best of our knowledge, this is the first study utilizing multiple diverse question banks to investigate the characteristics of various online platforms and doctors at different levels about assisting parents in understanding online health information about congenital cataract. Our study found that the appropriate use of ChatGPT, regardless of the type of question, could provide online health information about congenital cataracts which was at least comparable in quality to that offered by senior attending doctors.

There is a significant difference between the content of the two question banks. The Google question bank focuses more on epidemiology, disease progression and clinical management, while neglecting postoperative care and long-term follow-up. In contrast, the Doctor question bank places more emphasis on these aspects. It is understandable that people tend to be more concerned about the surgery and the disease itself, especially when it comes to infants, while postoperative needs are often left to the decision of the doctor. However, long-term management after congenital cataract surgery is crucial for visual recovery. The differences between the two question banks also highlight the need for parents to seek medical health information through a variety of sources.

This study assessed the credibility of 30 websites sourced from Google responses using JAMA benchmarks and found that only one webpage met all four criteria. The average score for the Doctor Bank was higher, likely because the questions in the Doctor Bank were more complex and open-ended, requiring higher standards from the webpages and thus yielding greater credibility. Recently, a study by Cohen et al. addressed 20 questions in the fields of “Cataracts” and “Cataract Surgery,” with an average JAMA benchmark score of 1.4, which was lower than the scores of both question banks in this study (1.93 for Google Bank and 2.33 for Doctor Bank) (Cohen et al., 2024). This discrepancy may be due to the rarity of congenital cataracts, requiring higher qualifications for related websites.

According to the U.S. Department of Health and Human Services (USDHHS), online information should be written at a 7th to 8th grade reading level, which aligns with the average American reading ability (Edmunds et al., 2013). In our study, the average FKGL was over 13.00 and the average DCS exceeded 9.0 (college level), significantly higher than the recommended level. Additionally, all original groups had an FRE score below 40, which was considered difficult by USDHHS standards. The FKGL of congenital cataract responses on the Google platform in previous studies (12.4) was similar to our findings and higher than that for general cataracts (9.2), likely due to the greater complexity of vocabulary, syllable length, rare words, and question bank differences in congenital cataracts (Cohen et al., 2024; Elhusseiny et al., 2023). Notably, Doctor 2 demonstrated better readability across both question banks, highlighting the readability advantage of resident doctors compared to more experienced clinicians and other sources.

However, previous studies have shown that populations with lower health literacy are more likely to be affected by pediatric cataracts, and people in these regions may not have access to the necessary education (Gilbert and Foster, 2001). Fortunately, we can ask ChatGPT to provide responses with enhanced readability. After revision, the readability of ChatGPT’s answers improved significantly across both question banks. Surprisingly, even with simplified language, the revised answers retained nearly all the original characteristics. In fact, the readability, helpfulness, and safety scores in the Doctor question bank showed notable improvement. These results highlight the potential of ChatGPT as a valuable resource for delivering accessible and understandable medical information on congenital cataracts, particularly for populations with limited health literacy.

When considering overall response characteristics, Google performed poorly in both question banks. In contrast, Doctor 1 consistently scored the highest in correctness, completeness, readability, and safety, especially in the Doctor Bank. This may be due to the more flexible and context-specific nature of the questions in the doctor-focused bank. Unlike Google’s search engine, which cannot accurately interpret every scenario within a question, doctors regularly handle similar consultations, enabling them to provide more contextually accurate and comprehensive responses. Furthermore, ChatGPT demonstrated response capabilities comparable to Doctor 1 and significantly outperformed Doctor 2, particularly in terms of completeness. This can be attributed to the characteristics of LLMs, which tend to provide longer, more detailed answers, often including multiple points and suggestions for seeking further medical consultation. These features make ChatGPT the most suitable tool for initial consultations.

Despite the promising performance of ChatGPT in our evaluation—particularly in terms of readability and alignment with human-generated content—it is important to recognize the limitations of large language models in medical contexts. One of the most critical concerns is the potential for hallucinations, where the model generates information that is factually incorrect or misleading, despite appearing authoritative. Such occurrences can be especially problematic in high-stakes healthcare scenarios, where accuracy and safety are paramount. Therefore, we emphasize that LLMs like ChatGPT should be viewed as supplementary tools for patient education, not as standalone sources of clinical decision-making. Moreover, with the LLMs’ new multimodal capabilities, there are concerns about the potential for widespread misuse of LLMs within medical contexts. For instance, anyone with internet access can easily use a chatbot, which may lead to privacy breaches. Clear guidelines are essential to protect patient confidentiality and privacy, especially concerning medical images (Rabinovitch et al., 2025). Additionally, medicolegal challenges related to liability in cases of misdiagnosis or misleading recommendations remain significant concerns associated with LLMs (Sallam and Chat, 2023).

This study has several limitations. Firstly, the doctor question bank used in this study was derived from a single doctor, and future research will incorporate questions from a broader range of doctors to create a more comprehensive question bank. Secondly, the responses generated by the Google engine are recorded by professional doctors. However, the content across different webpages can vary, potentially leading to biases in the recorded answers. Furthermore, considering patients’ economic and cultural backgrounds, this study utilized a non-paid version of ChatGPT, which may have affected the readability and characteristics of the responses. We will try more different LLMs for further analysis in the future.

In conclusion, this pioneering study highlights the significant potential of utilizing diverse question banks to enhance the understanding of online health information regarding congenital cataracts. Our findings demonstrate that ChatGPT can generate responses that rival the quality of information provided by experienced doctors, particularly in its ability to improve readability, which is crucial for families with varying levels of health literacy. Additionally, the study emphasizes the importance of consulting multiple sources for comprehensive health information, as traditional platforms like Google may not adequately address key aspects of patient education. Overall, our research supports the use of advanced AI tools like ChatGPT as valuable resources for improving public access to understandable and accurate medical information, particularly for those facing challenges due to lower health literacy.

## Data Availability

The original contributions presented in the study are included in the article/Supplementary material, further inquiries can be directed to the corresponding authors.

## Footnotes

^1^ https://datayze.com/readability-analyzer
