# Supplementary material for: Online platform vs. doctors: a comparative exploration of congenital cataract patient education from virtual to reality
Source: Front Artif Intell. 2025 Jun 3;8:1548385. doi: 10.3389/frai.2025.1548385 (PMC12170551; doi:10.3389/frai.2025.1548385)
Supplement: Supplementary file 1 [file Table_1.docx]

| **Table S1 Questions and Classifications of the Two Question Banks** | |
| --- | --- |
| **Google Question Bank** | **Doctor Question Bank** |
| **Pathogenesis and epidemiology**  **(n=8, 53.33%)** | **Pathogenesis and epidemiology**  **(n=1, 6.67%)** |
| What are congenital cataracts? | Our eldest child has bilateral congenital cataracts, and we are considering having a second child. What is the likelihood of the second child developing cataracts? |
| What is the most common type of congenital cataract? | **Management**  **(n=9, 60.00%)** |
| Why was I born with cataracts? | Is the congenital cataract surgery risky? |
| What percent of people have congenital cataracts? | Do we have to proceed with the congenital cataract surgery now, or can we wait? |
| What is the difference between congenital cataract and developmental cataract? | Can we continue with amblyopia training and delay the congenital cataract surgery for a few years? |
| How serious are congenital cataracts? | After congenital cataract surgery, is it better to wear rigid gas-permeable lenses or regular spectacles? |
| What are the long term effects of congenital cataracts? | We have unilateral congenital cataract in our child. It's said that the surgical outcomes for unilateral cases are not as good. Is this true? |
| Does congenital cataract cause amblyopia? | After congenital cataract surgery in a child, is it necessary to regularly monitor intraocular pressure and axial length? |
| **Management**  **(n=5, 33.33%)** | How long after congenital cataract surgery should the child avoid physical education classes? |
| What is the minimum age for congenital cataract surgery? | Can a child have a multifocal intraocular lens implanted during congenital cataract surgery, or is a monofocal lens the only option? |
| How successful is congenital cataract surgery? | The severity of congenital cataracts in both of children's eyes is not consistent, should we do cataract surgery in both eyes, or do the severe eye first? |
| What is the most common complication of congenital cataract surgery? | **Prognosis**  **(n=5, 33.33%)** |
| What is the surgery of choice for congenital cataract? | Will the vision improve after congenital cataract surgery? |
| How are congenital cataracts optically corrected? | If an IOL is implanted now, will it need to be replaced when the individual reaches adulthood? |
| **Prognosis**  **(n=2, 13.33%)** | Will congenital cataract redevelop after the surgery? |
| What happens if congenital cataracts are left untreated? | The child currently has nystagmus. Will the nystagmus resolve after congenital cataract cataract surgery? |
| Is congenital cataract progressive? | The congenital cataract surgery has been completed, and now amblyopia training is required. However, all amblyopia training programs increase the duration and intensity of eye use, which could lead to a worsening of myopia. Is this a concern? |

| **Table S2 Readability of different responsers for both question banks** | | | | | | |
| --- | --- | --- | --- | --- | --- | --- |
| **Group** | **No.** | **Question** | **Response** | **FRE** | **FKGL** | **DCS** |
|  |  |  |  |  |  |  |
| **GG** | 1 | What are congenital cataracts? | A cataract is any clouding or opacity of the lens of an eye. Congenital means that it happens before birth or during a baby's first year of life. A baby with congenital cataracts has clouding in one or both eyes. | 66.4 | 7.31 | 6.67 |
|  | 2 | What is the most common type of congenital cataract? | In literature, lamellar congenital cataract is thought to be the most frequently encountered form of cataract in pediatric patients | 13.9 | 16.4 | 12.06 |
|  | 3 | Why was I born with cataracts? | Cataracts happen when proteins in the eye's lens change. They may change because of an infection, a change in DNA, or a chemical imbalance.  Congenital cataracts can happen in babies who:  had an infection before or soon after birth have a family history of congenital cataracts were born early (premature) The most common infections that cause congenital cataracts include: chickenpox cytomegalovirus herpes HIV rubella syphilis toxoplasmosis Many babies who develop congenital cataracts don't have other medical problems. In a lot of cases, a cause is not found. | 44.76 | 8.37 | 9.35 |
|  | 4 | What percent of people have congenital cataracts? | It is estimated that congenital cataracts are responsible for 5% to 20% of blindness in children worldwide. Incidence varies from country to country. One retrospective study of the prevalence of infantile cataracts in the U.S. showed a rate of 3-4 visually significant cataracts per 10,000 live births. | 42.16 | 9.56 | 10.17 |
|  | 5 | What is the difference between congenital cataract and developmental cataract? | These are known as childhood cataracts. Childhood cataracts are often referred to as: congenital cataracts – cataracts present when a baby is born or shortly afterwards. developmental, infantile or juvenile cataracts – cataracts diagnosed in older babies or children. | 25.12 | 12.82 | 10.65 |
|  | 6 | How serious are congenital cataracts? | A cataract is any light scattering opacity of the lens. It is estimated that congenital cataracts are responsible for 5% to 20% of blindness in children worldwide. | 38.48 | 11 | 9.94 |
|  | 7 | What are the long term effects of congenital cataracts? | It can profoundly impede normal visual development if not treated early or left untreated, resulting in permanently reduced vision (developing a “lazy eye”). | 10.61 | 17.49 | 12.33 |
|  | 8 | Does congenital cataract cause amblyopia? | Depending on the cause of a cataract and how big it is, a baby with a congenital cataract can have other eye problems, including: some vision loss (called "lazy eye" or amblyopia) a rip (tear) in the light-sensitive layer in the back of the eye (retinal detachment) | 27.69 | 21.32 | 9.64 |
|  | 9 | What is the minimum age for congenital cataract surgery? | Cataract surgery is the treatment of choice for visually significant congenital cataracts. To limit the severity of deprivation amblyopia, it is recommended that unilateral congenital cataracts be removed by 6 weeks of age and bilateral congenital cataracts be removed by 10 weeks of age. | 4.23 | 17.89 | 11.07 |
|  | 10 | What happens if congenital cataracts are left untreated? | Any obstruction (such as cataract) if left untreated may lead to permanent sight impairment or blindness, even if it is removed later. | 46.07 | 12.3 | 9.03 |
|  | 11 | How successful is congenital cataract surgery? | Removing a congenital cataract is usually a safe, effective procedure. The child will need follow-up for vision rehabilitation. Most infants with congenital cataract in one eye have some level of "lazy eye" (amblyopia) and will need to use patching after the surgery in an attempt to reverse it. | 40.05 | 11.73 | 8.96 |
|  | 12 | What is the most common complication of congenital cataract surgery? | If your child has an artificial lens fitted during cataract surgery, the main risk is a condition called posterior capsule opacification (PCO). This is where part of the lens capsule (the "pocket" that the lens sits inside) thickens and causes cloudy vision. | 46.53 | 11.99 | 10.69 |
|  | 13 | What is the surgery of choice for congenital cataract? | Lensectomy. In a lensectomy, most of the lens (including the posterior capsule) and anterior vitreous is removed. This leaves a permanently clear visual axis. However, it requires a vitrectomy machine, and any interference with the vitreous may increase the risk of late retinal detachment. | 36.08 | 10.96 | 11 |
|  | 14 | How are congenital cataracts optically corrected? | Usually, strong eyeglasses or contact lenses are prescribed for infants after surgery. For babies over one year of age, an artificial intraocular lens (IOL) may be recommended instead to replace the eye's natural lens. The ophthalmologist can recommend which procedure and optical correction is best for your child. | 31.97 | 12.78 | 10.68 |
|  | 15 | Is congenital cataract progressive? | Many more children suffer from partial cataracts that may slowly progress over time, increasing the visual difficulties as the child grows | 32.43 | 13.95 | 9.19 |
| **GD** | 1 | Will the vision improve after congenital cataract surgery? | The management of congenital cataract is very different to the treatment of a routine age-related cataract. In adults, surgery may be delayed for years without affecting the visual outcome. In infants, if the cataract is not removed during the first year of life, the vision will never be fully regained after surgery. | 42.05 | 11.78 | 9.58 |
|  | 2 | If an IOL is implanted now, will it need to be replaced when the individual reaches adulthood? | Unlike the natural lenses of the eye, IOLs do not break down and never need to be replaced. To reap these long-lasting benefits, patients should follow any aftercare instructions as given by their doctor following surgery. Complete recovery can be expected over 1 – 2 days, but this depends on your original eyesight and any underlying health conditions. | 44.37 | 11.71 | 9.07 |
|  | 3 | Is the congenital cataract surgery risky? | Removing a congenital cataract is usually a safe, effective procedure. The child will need follow-up for vision rehabilitation. Most infants with congenital cataract in one eye have some level of "lazy eye" (amblyopia) and will need to use patching after the surgery in an attempt to reverse it. | 40.05 | 11.73 | 8.96 |
|  | 4 | Do we have to proceed with the congenital cataract surgery now, or can we wait? | If the cataracts are present from birth, the operation will be carried out as soon as possible, usually 1 to 2 months after your baby is born. | 49.48 | 12.57 | 6.77 |
|  | 5 | Will congenital cataract redevelop after the surgery? | While your cataracts cannot come back after surgery, you can get what is called posterior capsular opacification (or PCO), also referred to as a secondary cataract. | 24.26 | 16.33 | 10.39 |
|  | 6 | Can we continue with amblyopia training and delay the congenital cataract surgery for a few years? | Deprivation amblyopia may be minimized with early surgery for dense congenital bilateral cataracts. During the first 14 weeks of life, there is a linear relationship between delay in surgery and longterm visual acuity outcome, with an average loss of one line of visual acuity for every three weeks of delay. After 14 weeks of age, up to 31 weeks of age, there is little additional adverse effect of further delay in surgery. | 32.68 | 14.41 | 10.5 |
|  | 7 | Our eldest child has bilateral congenital cataracts, and we are considering having a second child. What is the likelihood of the second child developing cataracts? | The causes of infantile cataracts have been the source of much speculation and research. Making a distinction between unilateral and bilateral cataracts may be useful when considering etiology.  The majority of bilateral congenital or infantile cataracts not associated with a syndrome have no identifiable cause. Genetic mutation is likely the most common cause. Over fifteen genes involved in cataract formation have been identified, and the inheritance is most often autosomal dominant although it can be X-linked or autosomal recessive.Within the same pedigree, there can be considerable morphologic variation.  Systemic associations include metabolic disorders such as galactosemia, Wilson disease, hypocalcemia and diabetes. Cataracts may be a part of a number of syndromes, the most common being trisomy 21. Intrauterine infections including rubella, herpes simplex, toxoplasmosis, varicella and syphilis are another cause.  In contrast, most unilateral cataracts are not inherited or associated with a systemic disease and are of unknown etiology although they do not rule out the possibility of an associated systemic disease. They are usually the result of local dysgenesis and may be associated with other ocular dysgenesis such as persistent fetal vasculature (PFV), posterior lenticonus or lentiglobus.  Trauma is a known cause of pediatric cataracts. If there is no known history of trauma to explain an acquired cataract in this age group, investigation must be considered in children who present with other signs suggestive of child abuse.  Regardless of the etiology, prompt treatment of visually significant cataracts is necessary to allow proper development of vision. | 13.22 | 15.8 | 11.48 |
|  | 8 | After congenital cataract surgery, is it better to wear rigid gas-permeable (RGP) lenses or regular spectacles? | RGPCL is a well-tolerated, safe, and effective treatment for infants and young children. The visual acuity and degree of nystagmus were significantly improved in children who wore RGPCL for aphakia refractive correction after congenital cataract surgery compared with spectacle correction. | 20.95 | 15.43 | 11.2 |
|  | 9 | The child currently has nystagmus. Will the nystagmus resolve after congenital cataract cataract surgery? | Amblyopia, strabismus, and nystagmus which may have developed prior to cataract surgery may continue despite removal of the cataract(s) and must also be addressed. In the postoperative period, it is important to prevent and keep under check any significant inflammatory reaction. | 22.36 | 15.36 | 11.07 |
|  | 10 | We have unilateral congenital cataract in our child. It's said that the surgical outcomes for unilateral cases are not as good. Is this true? | Pseudophakic eyes achieved better final visual acuity than aphakic eyes, and bilateral cataract patients had more favorable visual acuity outcomes than unilateral cataract patients. | 0 | 22.78 | 14.04 |
|  | 11 | After congenital cataract surgery in a child, is it necessary to regularly monitor intraocular pressure and axial length? | Elevation in intraocular pressure (IOP) is an early sign of postoperative glaucoma. Glaucoma does not always follow a slow course, as it can develop quickly. However, if affected children are not followed up regularly, the diagnosis may be delayed. | 35.29 | 11.57 | 9.14 |
|  | 12 | How long after congenital cataract surgery should the child avoid physical education classes? | The best guidance regarding when you can exercise after cataract surgery comes from your eye specialist. Be sure to stand by their recommendations since they may have knowledge of your specific situation.  The following timeline provides a basic guideline for when you can resume certain types of physical activity:  Week 1 following surgery  You can engage in low-impact physical activities the week following surgery, such as:  Walking outside;   Light stretching;  Mild treadmill exercise;  Simple household work;  Avoid bending over or lifting anything that weighs more than 10 to 15 pounds, including groceries and laundry. Your eyes may become more stressed as a result, which might prevent appropriate recovery.    Week 2 following surgery  Your doctor might give you the go-ahead for moderate-intensity exercises after two weeks, including:  Yoga;  Jogging;  Brisk walking;  Slow dancing;  4 to 6 weeks following surgery;  You should be able to start doing more strenuous physical activities again. These may consist of:  Lifting weights;  Running;  Swimming;  Hard cycling;  Once more, consult your ophthalmologist before starting this intensity of exercise. | 47.43 | 8.31 | 9.14 |
|  | 13 | Can a child have a multifocal intraocular lens implanted during congenital cataract surgery, or is a monofocal lens the only option? | Monofocal Lens is generally the preferred lens type for babies with congenital cataracts. | 24.44 | 13.08 | 12.78 |
|  | 14 | The severity of congenital cataracts in both of children's eyes is not consistent, should we do cataract surgery in both eyes, or do the severe eye first? | Dense cataracts that are likely to interfere with your baby's vision are usually removed very early, during the first few months of life. If one eye has a denser cataract and the other has a smaller or less dense cataract, then the eye with the denser cataract would usually be treated first. | 50.29 | 12.7 | 8.87 |
|  | 15 | The congenital cataract surgery has been completed, and now amblyopia training is required. However, all amblyopia training programs increase the duration and intensity of eye use, which could lead to a worsening of myopia. Is this a concern? | In congenital/developmental cataract patients who underwent cataract extraction and primary IOL implantation at age 2–3 years, eyes with longer pre-operative ALs had a slower myopic shift than those with average pre-operative ALs 3 years after surgery. This finding could have implications for the target refraction decision in congenital/developmental cataract surgery. | 5.12 | 18.88 | 13.88 |
| **CG** | 1 | What are congenital cataracts? | Congenital cataracts are a condition where a baby is born with clouding of the eye's lens, which can interfere with vision. The lens is a transparent structure inside the eye that helps focus light onto the retina, allowing us to see clearly. When the lens becomes cloudy, light cannot pass through properly, leading to blurred or impaired vision. | 55.94 | 10.26 | 9.5 |
|  | 2 | What is the most common type of congenital cataract? | The most common type of congenital cataract is the "nuclear cataract." This type of cataract affects the central part of the lens, known as the nucleus. Nuclear cataracts often have a characteristic appearance where the central part of the lens becomes cloudy or opaque, which can impact the clarity of vision.  Here are a few key points about nuclear cataracts:  Appearance: Nuclear cataracts appear as a cloudiness or opacity in the center of the lens. In infants, this may be observed as a white or grayish discoloration in the pupil.  Causes: Nuclear cataracts can be caused by genetic factors, and they may be associated with certain inherited conditions or metabolic disorders. They can also occur in isolation without any other health issues.  Detection: In infants and young children, nuclear cataracts are often detected during routine eye exams or when parents notice abnormalities in the child’s vision. Early detection is important to manage the condition effectively.  Treatment: Treatment typically involves surgical removal of the cataractous lens, followed by the use of corrective lenses or other visual aids to support vision development.  While nuclear cataracts are common, there are also other types of congenital cataracts, such as cortical cataracts (which affect the outer edges of the lens) and posterior subcapsular cataracts (which affect the back of the lens). The specific type of cataract and its impact on vision will guide the treatment approach. | 39.38 | 12.18 | 10.74 |
|  | 3 | Why was I born with cataracts? | Congenital cataracts, which are cataracts present at birth or develop shortly after, can be caused by a variety of factors. Sometimes, they occur due to genetic mutations or inherited conditions. Other times, they might be associated with certain infections or exposures that occurred during pregnancy, such as rubella or other illnesses. In some cases, the exact cause isn’t clear.Congenital cataracts can vary in severity and impact on vision. It's important to have regular check-ups to monitor and manage the condition. If you have any concerns or questions about your specific situation, it’s best to discuss them with your ophthalmologist, who can provide personalized information and guidance based on your case. | 38.77 | 11.83 | 9.22 |
|  | 4 | What percent of people have congenital cataracts? | Congenital cataracts are relatively rare, occurring in about 1 to 6 cases per 10,000 live births. This equates to 0.01% to 0.06% of people being born with cataracts. While the exact incidence can vary slightly based on region and access to healthcare, congenital cataracts are considered uncommon compared to other eye conditions. Early detection and treatment, typically in infancy or early childhood, are crucial for preserving vision and preventing long-term visual impairment. | 33.6 | 11.39 | 10.93 |
|  | 5 | What is the difference between congenital cataract and developmental cataract? | Congenital cataracts and developmental cataracts refer to cataracts that occur at different stages of life, and they have distinct characteristics and causes:  Congenital Cataract: Definition: Congenital cataracts are present at birth or develop shortly after birth. Causes: They can result from genetic factors, infections during pregnancy (such as rubella or toxoplasmosis), metabolic disorders, or other conditions affecting the fetus. Characteristics: Congenital cataracts may be present in one or both eyes and can vary in size, opacity, and impact on vision. They are often detected during newborn screening or early in infancy. Management: Treatment typically involves early surgical intervention to remove the cataract and restore clear vision, along with corrective lenses or other aids to support visual development. Developmental Cataract: Definition: Developmental cataracts occur during childhood, usually after the first few months of life, as the child grows and their vision develops. Causes: These cataracts can develop due to various factors, including trauma, certain systemic diseases, or can be idiopathic (with no known cause). They might also be associated with other eye conditions or systemic disorders. Characteristics: Developmental cataracts might not be present at birth but can develop over time as the child grows. They may be more progressive and can affect one or both eyes. Management: The approach to treating developmental cataracts depends on their severity, impact on vision, and underlying cause. Treatment options might include monitoring, corrective lenses, or surgery, depending on how the cataracts affect the child’s vision and overall development. Summary of Differences: Timing: Congenital cataracts are present at or near birth, while developmental cataracts develop later during childhood. Causes: Congenital cataracts are often due to prenatal factors or genetic conditions, whereas developmental cataracts may result from postnatal factors or progress over time. Management: Both types may require surgical intervention and corrective measures, but the timing and specific approach can differ based on when the cataracts are detected and their impact on vision. Understanding these differences helps tailor the approach to diagnosis and treatment, aiming for the best possible visual outcomes for the individual. | 16.76 | 16.15 | 10.82 |
|  | 6 | How serious are congenital cataracts？ | Congenital cataracts can be quite serious, particularly if they are left untreated or not managed appropriately. Their impact largely depends on several factors, including the size and location of the cataract, whether one or both eyes are affected, and the timing of diagnosis and intervention. Here’s an overview of why they can be serious:  Impact on Vision Visual Impairment: Congenital cataracts obstruct light from reaching the retina, which can severely impair vision. If the cataract is large or dense, it can significantly reduce visual acuity or even cause blindness in the affected eye.  Developmental Delays: For infants and young children, vision development is crucial during early years. Untreated cataracts can interfere with normal visual development, leading to issues such as amblyopia (lazy eye), where the brain begins to ignore input from the affected eye.  Potential Complications Amblyopia: This is a common complication of congenital cataracts. If the cataract prevents clear vision, the brain may not develop proper visual pathways, leading to a condition where the affected eye has poor vision despite the presence of a normal eye.  Strabismus: Misalignment of the eyes can occur if the vision is impaired, further complicating visual function and requiring additional treatment.  Visual Development: Congenital cataracts can disrupt normal visual development, which may affect the child’s ability to perform tasks that require good vision, such as reading and writing.  Treatment Considerations Early Intervention: Early diagnosis and treatment are crucial. Surgical removal of the cataract is often necessary to restore vision and prevent developmental issues. The timing of the surgery is critical to support normal visual and cognitive development.  Ongoing Care: After surgery, the child may require corrective lenses or other visual aids, and regular follow-up care is needed to monitor visual development and manage any complications.  Long-term Outcomes With timely and effective treatment, many children with congenital cataracts can achieve good visual outcomes. However, untreated congenital cataracts can lead to permanent vision impairment and developmental delays. The severity of these outcomes can vary based on the individual circumstances and the success of the interventions.  In summary, congenital cataracts can be serious due to their potential to significantly impact vision and visual development. Early detection and appropriate treatment are essential to minimize these risks and support the best possible visual outcomes for the child. | 23.09 | 14.14 | 11.35 |
|  | 7 | What are the long term effects of congenital cataracts? | The long-term effects of congenital cataracts can vary depending on several factors, including the timing of diagnosis, the type and severity of the cataract, and the effectiveness of treatment. Here are some potential long-term effects:  Visual Impairment Reduced Visual Acuity: Even with treatment, some children may experience reduced visual acuity in the affected eye. This can affect their ability to see clearly, especially if the cataract was dense or involved both eyes.  Amblyopia (Lazy Eye): If the cataract was not treated promptly, the affected eye might develop amblyopia, where the brain starts to ignore visual input from that eye. This condition can lead to long-term vision impairment if not addressed with additional therapies.  Visual Development and Function Binocular Vision Issues: Proper alignment and coordination of both eyes can be disrupted, leading to problems with depth perception and binocular vision. Strabismus (eye misalignment) may develop, which could require further treatment.  Difficulty with Low Vision Tasks: Even after cataract surgery, some children may have difficulty with tasks requiring fine visual discrimination, such as reading or participating in sports.  Psychosocial Impact Educational Challenges: Vision issues can impact learning and academic performance. Children might need special accommodations or support in educational settings to help them succeed.  Social and Emotional Effects: Vision problems can affect self-esteem and social interactions. Children with visual impairments may face challenges in social settings and may need additional support to build confidence and social skills.  Need for Ongoing Care Refractive Errors: Post-surgery, children might need corrective lenses to manage refractive errors such as nearsightedness or farsightedness. Regular eye exams are essential to monitor these changes and update prescriptions as needed.  Potential for Further Surgery: In some cases, additional surgeries or treatments may be needed over time to address complications or manage residual vision problems.  Risk of Other Eye Conditions Glaucoma: Children who have had cataract surgery might be at an increased risk of developing glaucoma, a condition where increased pressure in the eye can damage the optic nerve.  Retinal Issues: Depending on the severity of the cataract and the success of surgery, there may be an increased risk of retinal issues that require monitoring.  Quality of Life Adaptation and Support: With appropriate treatment and support, many children adapt well and lead normal lives. Early intervention and ongoing care play a critical role in ensuring the best possible outcomes.  Functional Independence: Many children with congenital cataracts can achieve functional independence and participate in a wide range of activities, though they may need accommodations and support.  Overall, while congenital cataracts can have significant long-term effects, early diagnosis and effective treatment greatly improve the prognosis. Regular follow-up care is essential to manage any ongoing issues and support optimal visual development and quality of life. | 26.39 | 13.64 | 11.21 |
|  | 8 | Does congenital cataract cause amblyopia? | Yes, congenital cataracts can cause amblyopia, often referred to as "lazy eye." Amblyopia is a condition where one eye has poor vision because the brain and eye are not working together properly. Here's how congenital cataracts can lead to amblyopia:  Mechanism Obstruction of Clear Vision: Congenital cataracts cause a clouding of the lens, which obstructs light from reaching the retina. This prevents the affected eye from sending clear visual signals to the brain.  Visual Development: In infants and young children, visual development is crucial during early years. If one eye is not providing clear visual input, the brain may start to favor the other eye. This can lead to a situation where the affected eye does not develop proper visual pathways.  Brain's Adaptation: The brain may begin to ignore or suppress the input from the affected eye because it is receiving less clear or no visual information. This adaptation results in the brain relying predominantly on the stronger eye.  Consequences Reduced Vision: The affected eye develops poorer vision compared to the unaffected eye, even though the eye itself may be structurally normal after treatment.  Difficulty in Binocular Vision: Amblyopia can impact the ability to use both eyes together effectively. This can lead to issues with depth perception and binocular vision.  Prevention and Treatment Early Intervention: Early diagnosis and treatment of congenital cataracts are crucial in preventing or minimizing amblyopia. If a cataract is detected and treated early, there is a better chance of supporting normal visual development and reducing the risk of amblyopia.  Surgical Treatment: Surgery to remove the cataract is often necessary. After surgery, corrective lenses or other visual aids are typically used to help the child achieve clear vision.  Vision Therapy: In cases where amblyopia develops, additional treatments such as vision therapy or patching the stronger eye may be needed to encourage the brain to use the affected eye more effectively.  Ongoing Monitoring: Regular follow-up care is important to monitor visual development and address any issues that may arise.  In summary, congenital cataracts can indeed cause amblyopia if not treated promptly. Early diagnosis and intervention are key to minimizing the risk of amblyopia and supporting optimal visual development. | 30.84 | 12.86 | 10.36 |
|  | 9 | What is the minimum age for congenital cataract surgery? | The minimum age for congenital cataract surgery is not strictly defined by a specific age but rather depends on the individual child's condition and the timing of diagnosis. However, in general practice:  Typical Timing Early Intervention: Ideally, congenital cataract surgery is performed as early as possible to minimize the risk of vision problems. Surgery is often considered within the first few months of life.  Timing for Surgery: Most ophthalmologists aim to perform cataract surgery within the first 6 to 12 weeks of life if the cataract is causing significant visual impairment or is likely to affect visual development.  Factors Influencing Timing Severity of Cataract: The size, density, and location of the cataract can influence the timing of surgery. More severe cataracts may require earlier intervention.  Visual Impact: The potential impact on vision and visual development is a critical factor. If the cataract significantly obstructs vision and affects normal visual development, earlier surgery is prioritized.  Health and Stability of the Infant: The overall health and stability of the infant are also considered. Surgery may be postponed if there are other medical issues that need to be addressed first.  Ophthalmologist’s Recommendations: The decision on timing is guided by the ophthalmologist based on the individual child’s condition and needs.  Benefits of Early Surgery Prevention of Amblyopia: Early surgery helps to prevent or minimize the risk of amblyopia (lazy eye) and supports proper visual development.  Normal Visual Development: Early intervention helps in achieving better visual outcomes and supports normal visual and cognitive development.  Reduction in Long-Term Complications: Performing surgery early can reduce the risk of complications associated with untreated congenital cataracts, such as severe visual impairment and developmental delays.  Follow-Up and Care After surgery, ongoing follow-up care is crucial to monitor the child’s vision and ensure proper visual development. This may include the use of corrective lenses, vision therapy, and regular eye exams to track progress and address any additional issues.  In summary, while there is no fixed minimum age for congenital cataract surgery, it is generally recommended to perform the surgery as early as possible, often within the first few months of life, to optimize visual outcomes and support normal development. | 21.46 | 14.51 | 11.3 |
|  | 10 | What happens if congenital cataracts are left untreated? | If congenital cataracts are left untreated, they can lead to several serious issues, particularly concerning vision development in children. Here’s what might happen:  Visual Impairment: Congenital cataracts can obstruct light from reaching the retina, which is crucial for clear vision. If the cataract is not addressed, it can lead to significant visual impairment or even blindness in the affected eye.  Amblyopia (Lazy Eye): The obstruction caused by the cataract can prevent the brain from receiving clear images from the affected eye. This can result in amblyopia, where the brain starts to ignore input from that eye, leading to poor vision development.  Poor Visual Development: For infants and young children, vision development is a critical process that needs to occur during certain developmental stages. Untreated cataracts can disrupt this process, potentially leading to long-term visual deficits.  Strabismus (Eye Misalignment): The lack of clear vision can lead to eye misalignment or strabismus, where the eyes do not work together properly. This can further complicate vision issues and may require additional treatment.  Impact on Daily Activities: Vision problems resulting from untreated cataracts can affect a child's ability to perform everyday tasks, including reading, writing, and other activities that require good vision.  It’s important to diagnose and treat congenital cataracts as early as possible to minimize these risks and help ensure the best possible visual outcomes for the child. Treatment typically involves surgery to remove the cataract and may include additional measures such as corrective lenses or vision therapy to support optimal visual development. | 27.81 | 14.18 | 10.82 |
|  |  | What is the success rate of congenital cataract surgery? | The success rate of congenital cataract surgery is generally high, especially when the procedure is performed early and followed by appropriate post-operative care. However, the exact success rate can vary based on several factors, including:  1. Timing of Surgery Early Intervention: When congenital cataract surgery is performed early, typically within the first few months of life, the success rate is generally higher. Early surgery helps prevent or minimize issues such as amblyopia and supports normal visual development. 2. Type and Severity of Cataract Cataract Characteristics: The success of the surgery can depend on the size, density, and location of the cataract. More complex or dense cataracts may pose additional challenges. 3. Surgical Technique and Expertise Experienced Surgeons: The skill and experience of the ophthalmic surgeon play a crucial role in the success of the surgery. Surgeons with expertise in pediatric cataract surgery are more likely to achieve favorable outcomes. 4. Post-Operative Care Rehabilitation and Follow-Up: Successful outcomes also depend on proper post-operative care, including the use of corrective lenses, adherence to follow-up appointments, and monitoring for any complications. 5. Individual Factors Overall Health of the Child: The overall health and stability of the child, as well as any other existing health conditions, can influence the success of the surgery. General Success Rates Visual Outcomes: Studies generally show that the majority of children who undergo congenital cataract surgery achieve significant improvement in vision. Reports suggest that approximately 70-80% of children can achieve a functional level of vision with early intervention and appropriate follow-up care.  Complication Rates: While the success rate for improving vision is high, there can be complications, such as secondary glaucoma, lens displacement, or retinal issues. However, with proper management, many of these complications can be addressed effectively.  Long-Term Outcomes Visual Development: Early surgery and follow-up care can lead to good long-term visual outcomes, including the development of normal vision and visual skills. Regular monitoring and management are essential to address any issues that may arise as the child grows. In summary, congenital cataract surgery has a high success rate, particularly when performed early and supported by thorough post-operative care. The majority of children achieve significant improvements in vision, although individual outcomes can vary based on specific factors related to the cataract and the child’s overall health. | 16.42 | 15.88 | 10.88 |
|  | 12 | What is the most common complication of congenital cataract surgery? | The most common complication of congenital cataract surgery is posterior capsule opacification (PCO), also known as "secondary cataract." This occurs when the posterior part of the lens capsule, which is left intact during surgery to support an intraocular lens (IOL), becomes cloudy after cataract removal. In pediatric patients, particularly infants, the risk of PCO is higher because of the aggressive wound-healing response in their eyes. If left untreated, PCO can lead to significant visual impairment. To address this, surgeons often perform a posterior capsulotomy (removal of part of the posterior capsule) during the initial surgery, and sometimes an anterior vitrectomy (removal of some of the gel-like vitreous behind the lens). This helps to reduce the chance of PCO formation and maintain clear visual pathways. | 27.06 | 14.7 | 11.7 |
|  | 13 | What is the surgery of choice for congenital cataract? | The surgery of choice for congenital cataracts is phacoemulsification with intraocular lens (IOL) implantation. This procedure is commonly used due to its effectiveness and precision. Here’s an overview of the process and key aspects:  1. Phacoemulsification Procedure: Phacoemulsification is a technique where the cloudy lens is broken into small fragments using ultrasound waves. These fragments are then suctioned out of the eye. Advantages: This method is minimally invasive and allows for precise removal of the cataract with smaller incisions compared to traditional methods. 2. Intraocular Lens (IOL) Implantation Procedure: After the cataract is removed, an artificial lens, known as an intraocular lens (IOL), is implanted into the eye. This lens helps to restore focus and vision. Types of IOLs: The choice of IOL depends on the child's needs and the surgeon’s recommendations. Options include monofocal lenses, which provide clear vision at one distance, or accommodating lenses that adjust focus for near and far vision. In some cases, if the child is very young or has certain conditions, a contact lens may be used temporarily instead of an IOL. 3. Timing of Surgery Early Intervention: It’s generally recommended to perform the surgery as early as possible, often within the first few months of life, to minimize the risk of visual development issues such as amblyopia (lazy eye) and to support proper visual development. 4. Post-Operative Care Follow-Up: After surgery, regular follow-up visits are essential to monitor the child’s vision, ensure proper healing, and manage any complications. Corrective Measures: The child may need corrective lenses (glasses or contact lenses) to optimize visual acuity after the surgery. 5. Benefits Improved Vision: The primary goal of the surgery is to restore clear vision and support normal visual development. Reduced Complications: Phacoemulsification with IOL implantation is associated with a lower risk of complications compared to older surgical techniques and provides effective visual rehabilitation. In summary, phacoemulsification with intraocular lens implantation is the preferred surgical approach for congenital cataracts due to its effectiveness in removing the cloudy lens and restoring vision. Early intervention and careful post-operative care are crucial to achieving the best outcomes. | 22.06 | 14.9 | 11.37 |
|  | 14 | How are congenital cataracts optically corrected? | Congenital cataracts can be optically corrected through several methods, depending on the specifics of the cataract and the child’s visual needs. Here’s how congenital cataracts are typically managed and corrected optically: 1. Surgical Correction Phacoemulsification and Intraocular Lens (IOL) Implantation Procedure: After cataract removal, an intraocular lens (IOL) is implanted to replace the cloudy lens. This lens helps restore clear vision by focusing light properly onto the retina. Types of IOLs: The choice of IOL depends on the child's age and specific needs. For very young children, an IOL may be placed at the time of cataract surgery, or the child may initially use contact lenses until the eye grows enough to accommodate an IOL. 2. Optical Correction with Lenses Glasses Use: Post-surgery, glasses are often used to correct refractive errors and provide the best possible vision. Glasses can help manage any residual refractive issues such as nearsightedness (myopia), farsightedness (hyperopia), or astigmatism. Customization: The prescription for glasses will be tailored based on the child’s specific vision needs and any changes in vision following surgery. Contact Lenses Use: In some cases, particularly if IOL implantation isn’t possible or as a temporary measure before the eye grows enough for an IOL, contact lenses may be used. They provide a more flexible solution for vision correction and can be adjusted as needed. Benefits: Contact lenses can provide a wider field of vision and reduce issues related to the fit of glasses. 3. Vision Therapy Rehabilitation Use: In cases where there are issues such as amblyopia (lazy eye) or difficulties with binocular vision, vision therapy or patching may be recommended. This helps to improve the coordination between the eyes and encourages the use of the affected eye. Techniques: Therapy may include exercises to strengthen eye muscles and improve visual skills. 4. Follow-Up Care Regular Monitoring Importance: Ongoing follow-up visits with an ophthalmologist are essential to monitor visual development, adjust optical prescriptions, and address any complications or changes in vision. Adjustments: Regular eye exams help ensure that any adjustments to glasses or contact lenses are made as needed to support optimal vision. Summary Optical correction for congenital cataracts involves a combination of surgical intervention, such as phacoemulsification with IOL implantation, and post-surgical correction with glasses or contact lenses. Vision therapy may be used to address additional visual development issues. Regular follow-up and monitoring are crucial to ensure the best visual outcomes and adjust treatments as needed. | 32.19 | 12.11 | 10.57 |
|  | 15 | Is congenital cataract progressive? | Congenital cataracts are typically not progressive in the sense that they do not usually worsen over time once they are present. However, there are some important nuances to consider: 1. Nature of Congenital Cataracts Stable After Birth: Once a congenital cataract is formed, it generally remains stable in terms of its opacification. Unlike age-related cataracts, which tend to worsen progressively over time, congenital cataracts don’t usually change significantly in density or size after birth. 2. Potential for Complications Progressive Visual Issues: While the cataract itself may not progress, untreated congenital cataracts can lead to progressive visual issues due to their impact on vision development. For instance: Amblyopia (Lazy Eye): If a congenital cataract obstructs vision, it can cause amblyopia, where the affected eye's vision does not develop normally. This can lead to worsening visual function if not addressed. Strabismus (Eye Misalignment): Ongoing visual impairment can lead to strabismus, which may worsen if the visual issues are not corrected. 3. Surgical and Post-Surgical Considerations Surgical Outcomes: After cataract surgery, while the cataract itself is removed, some children may develop other issues such as posterior capsule opacification (PCO), where the membrane behind the lens becomes cloudy. This condition can affect vision but is typically treatable with a minor laser procedure. Vision Development: Even after successful cataract removal and optical correction, some children may face challenges related to visual development. Ongoing monitoring and adjustments are necessary to manage these issues effectively. 4. Management and Follow-Up Regular Monitoring: Regular eye exams are essential to monitor for any changes in vision and to ensure that optical corrections (glasses, contact lenses) are appropriate. This helps address any new or arising visual issues promptly. In summary, while congenital cataracts themselves are not typically progressive once formed, their impact on vision can lead to secondary issues that may progress if not treated. Early diagnosis and timely surgical intervention are crucial to preventing these complications and supporting normal visual development. Regular follow-up care is important to manage any potential post-surgical or developmental issues. | 19.65 | 14.3 | 11.18 |
| **CD** | 1 | Will the vision improve after congenital cataract surgery? | Yes, vision often improves significantly after congenital cataract surgery, especially if the cataract was obstructing vision. The primary goal of the surgery is to remove the cloudy lens and replace it with a clear artificial intraocular lens (IOL) or to use contact lenses or glasses to correct vision if an IOL isn't suitable.  Here are a few key points to consider:  Early Intervention: The earlier the surgery is performed, the better the visual outcomes tend to be. Early intervention helps in the proper development of visual pathways and reduces the risk of amblyopia (lazy eye) and other complications.  Rehabilitation: Post-surgery, vision improvement may take some time as the brain adjusts to the new visual input, especially in very young children. Vision therapy and regular follow-up care are important.  Visual Outcomes: While many individuals experience significant improvement in vision after surgery, the final outcome can vary based on factors such as the severity of the cataract, the presence of any other eye conditions, and how well the brain adapts to the corrected vision.  Your ophthalmologist will provide guidance on what to expect and how to support the best possible visual outcomes following surgery. | 29.27 | 14.53 | 10.4 |
|  | 2 | If an IOL is implanted now, will it need to be replaced when the individual reaches adulthood? | In most cases, once an intraocular lens (IOL) is implanted, it doesn’t need to be replaced as the person grows older. IOLs are designed to be durable and to last a lifetime. However, there are a few exceptions and considerations:  Lens Power Changes: If the child’s eye continues to grow significantly after the IOL is implanted, the power of the lens might no longer be optimal. This might necessitate a lens replacement or adjustment in adulthood, but it's relatively rare.  Complications or Changes: If there are complications or if the lens becomes dislocated or causes other issues, it might need to be replaced or adjusted.  Advances in Technology: In some cases, advancements in IOL technology might offer improved options, and a replacement could be considered for enhanced vision or comfort.  Regular follow-up appointments with your ophthalmologist will help ensure that the IOL continues to meet your vision needs and address any issues that might arise. | 44.42 | 11.91 | 9.77 |
|  | 3 | Is the congenital cataract surgery risky? | Congenital cataract surgery is generally safe and effective, but, like any surgical procedure, it does come with some risks and potential complications. Here's a summary of what you might expect:  Risks and Complications: Infection: As with any surgery, there's a risk of infection, though this is relatively rare due to modern sterile techniques and antibiotics.  Inflammation: Post-operative inflammation can occur, but it's usually manageable with medication.  Glaucoma: Increased eye pressure can sometimes develop after surgery, but this is usually monitored and treated if necessary.  Retinal Detachment: Though rare, there's a slight risk of retinal detachment, especially in young children whose eyes are still developing.  Lens Dislocation: The intraocular lens (IOL) could become dislocated, which may require additional surgery.  Visual Outcomes: While many patients achieve excellent vision post-surgery, some may still have visual issues or require additional interventions such as glasses, contact lenses, or further surgeries.  Benefits and Considerations: Improved Vision: The primary benefit is a significant improvement in vision, especially if the cataract was impairing sight.  Early Intervention: Performing surgery at an early age can help prevent or minimize the impact on visual development and reduce the risk of complications such as amblyopia.  Long-term Monitoring: Regular follow-up appointments are crucial to monitor for any issues and to ensure the best possible visual outcomes.  Overall, congenital cataract surgery is a well-established procedure with a high success rate. Discussing the potential risks and benefits with your ophthalmologist can help you make an informed decision and prepare for the recovery process. | 22.52 | 14.31 | 11.65 |
|  | 4 | Do we have to proceed with the congenital cataract surgery now, or can we wait? | Deciding whether to proceed with congenital cataract surgery immediately or to wait involves several important considerations. Here’s a general overview to help you understand the factors that might influence this decision:  Reasons to Consider Early Surgery: Visual Development: Early intervention is crucial for proper visual development. The visual pathways in the brain are still developing in infants and young children. If a congenital cataract is left untreated, it can lead to long-term vision problems, such as amblyopia (lazy eye) or poor visual acuity.  Preventing Complications: Untreated cataracts can cause additional complications, such as strabismus (crossed eyes) or further visual impairment, which might be harder to manage later.  Improved Outcomes: Studies have shown that earlier surgery often leads to better visual outcomes and reduces the need for additional corrective measures later on.  Factors to Consider Before Deciding to Wait: Severity of the Cataract: The urgency of surgery can depend on the severity of the cataract and how much it is affecting vision. If the cataract is not significantly impairing vision or causing other issues, your ophthalmologist might recommend monitoring the situation.  Age and Overall Health: The child’s overall health and readiness for surgery are important. In some cases, your ophthalmologist might recommend waiting if they believe that the child is too young or if there are other health considerations.  Potential for Natural Resolution: In rare cases, congenital cataracts might have the potential to improve on their own, although this is not common.  Making the Decision: Consultation: It’s important to have a detailed discussion with your ophthalmologist about the specific characteristics of the cataract, the potential risks of waiting, and the benefits of early surgery.  Regular Monitoring: If the decision is made to wait, regular eye exams are crucial to monitor any changes in the cataract and to ensure that it is not adversely affecting the child's vision.  Ultimately, the decision will be based on the specific details of the cataract and the child's visual needs. Your ophthalmologist can provide personalized advice and help you make the best choice for your child’s health and development. | 29.34 | 13.66 | 9.86 |
|  | 5 | Will congenital cataract redevelop after the surgery? | Congenital cataracts typically do not redevelop after surgery, but there are some important considerations to be aware of:  Possible Post-Surgical Complications: Posterior Capsule Opacification (PCO): Sometimes, after the removal of a congenital cataract, the thin membrane (capsule) that holds the artificial lens in place can become cloudy or opacified. This is not a reoccurrence of the original cataract but a new issue that can affect vision. PCO can be treated with a relatively simple outpatient procedure called YAG laser capsulotomy.  Lens Dislocation or Opacification: In rare cases, the intraocular lens (IOL) that is implanted during surgery can become dislocated or develop problems, which might affect vision. This is not the cataract returning but a different issue that might require intervention.  Other Eye Conditions: If there were underlying or associated eye conditions, they may still require attention and monitoring after the cataract surgery.  Long-Term Monitoring: Regular Follow-Up: Even after successful cataract surgery, regular eye exams are important to monitor the health of the eye and the performance of the IOL.  Visual Development: Especially in children, continuous follow-up is crucial for assessing visual development and addressing any issues that may arise as the child grows.  While the risk of the original congenital cataract redeveloping is not present, ongoing eye care and monitoring are essential to ensure the best visual outcomes and to address any new concerns that might arise. | 21.76 | 15.01 | 10.74 |
|  | 6 | Can we continue with amblyopia training and delay the congenital cataract surgery for a few years? | While amblyopia (lazy eye) training can be a crucial part of managing visual development, it generally cannot replace the need for congenital cataract surgery if the cataract is significantly impairing vision. Here’s why:  Importance of Early Surgery: Visual Development: For infants and young children, the primary goal is to prevent or minimize visual impairment. Cataracts that obstruct vision can interfere with the development of the visual pathways in the brain. Early removal of the cataract helps ensure proper visual development and reduces the risk of amblyopia.  Amblyopia Prevention: If a congenital cataract is left untreated, it can lead to amblyopia, where one eye becomes weaker than the other. Amblyopia treatment, such as patching or vision therapy, is more effective when initiated early, but it works best when the cataract is already removed.  Risks of Delaying Surgery: Progressive Visual Impairment: Delaying surgery can lead to progressive vision loss and make the treatment of amblyopia more challenging. The longer a cataract obstructs vision, the harder it may be to achieve optimal visual outcomes, even with amblyopia training.  Complications: Untreated cataracts can lead to additional complications, such as strabismus (crossed eyes) or further visual impairment, which might complicate treatment and management later on.  Possible Approach: Consult Your Ophthalmologist: It’s essential to discuss the specific details of the cataract and your child’s visual needs with your ophthalmologist. They can provide personalized advice based on the severity of the cataract and the potential impact on vision.  Combine Strategies: In some cases, an ophthalmologist might recommend proceeding with surgery while also continuing amblyopia training to address any visual development issues that arise post-surgery.  In general, early surgery is often recommended to prevent the complications associated with congenital cataracts and to provide the best foundation for visual development. Your ophthalmologist can help you weigh the benefits of early surgery against the potential advantages of any other treatments or delays. | 20.8 | 14.68 | 11.01 |
|  | 7 | Our eldest child has bilateral congenital cataracts, and we are considering having a second child. What is the likelihood of the second child developing cataracts? | The likelihood of a second child developing congenital cataracts can be influenced by several factors, including genetic and environmental considerations. Here’s a general overview:  Genetic Factors: Inherited Conditions: If the congenital cataracts in your eldest child are due to a genetic condition, there may be an increased risk for the second child. Some genetic disorders associated with congenital cataracts can be passed from parents to children. In such cases, genetic counseling might be recommended to assess the risk more accurately.  Genetic Counseling: Consulting with a genetic counselor can help you understand the risk based on your family's specific genetic background and any known hereditary conditions. They can provide information on the likelihood of recurrence and the available options for screening or early intervention.  Environmental and Other Factors: Non-Genetic Causes: Congenital cataracts can also result from non-genetic factors, such as prenatal infections (e.g., rubella) or exposure to certain substances during pregnancy. If your eldest child’s cataracts were due to such factors, the risk to a second child might be lower, but it’s still important to maintain good prenatal care and avoid known risks.  Individual Risk Assessment: Each pregnancy is unique, and the risk of congenital cataracts in a second child may be different from that in the first child. Factors such as the health of the pregnancy and any prenatal issues should be monitored by your healthcare provider.  Next Steps: Consult Your Healthcare Provider: Speak with your obstetrician or a genetic counselor about your family’s history and any concerns you may have. They can provide personalized advice based on your specific situation.  Prenatal Care: Ensure that you receive regular prenatal care to monitor the health of the pregnancy and to address any potential risks.  Overall, while there may be a higher risk if the congenital cataracts were due to a genetic condition, each pregnancy is unique. Consulting with a specialist will provide the most accurate information and help you make informed decisions. | 36.86 | 11.95 | 10.54 |
|  | 8 | After congenital cataract surgery, is it better to wear rigid gas-permeable (RGP) lenses or regular spectacles? | After congenital cataract surgery, the choice between rigid gas-permeable (RGP) lenses and regular spectacles depends on several factors, including the specific needs of the patient, the quality of vision achieved with the intraocular lens (IOL), and personal comfort. Here’s a comparison to help understand the options:  Rigid Gas-Permeable (RGP) Lenses: Advantages:  Visual Acuity: RGP lenses can provide excellent visual acuity, especially if there are irregularities in the cornea or if the IOL power is not perfectly corrected with spectacles. Stability: They offer a stable and precise optical correction, which can be beneficial if there is any irregular astigmatism. Durability: RGP lenses are generally durable and resistant to scratching. Disadvantages:  Comfort: They can be less comfortable than soft contact lenses or spectacles, particularly for people who are not accustomed to wearing them. Adaptation: Some people may require a period of adaptation to get used to wearing RGP lenses. Regular Spectacles: Advantages:  Ease of Use: Spectacles are easy to use and don’t require direct contact with the eye, making them a non-invasive option. Comfort: Many people find spectacles more comfortable than contact lenses. Less Maintenance: They are easier to clean and maintain compared to contact lenses. Disadvantages:  Vision Correction Limits: Spectacles may not correct certain types of vision issues as effectively as contact lenses, especially if there are significant visual distortions or irregularities. Peripheral Vision: Spectacles might not provide as wide a field of view as contact lenses. Factors to Consider: Visual Needs: If the child has specific visual needs or issues like astigmatism that are not fully corrected with spectacles, RGP lenses might be a better option.  Comfort and Lifestyle: Consider the comfort and lifestyle of the child. Young children, for example, might find it easier to adapt to spectacles rather than contact lenses.  Follow-Up Care: Regular follow-up appointments with the ophthalmologist or optometrist are crucial to ensure that the chosen corrective method is providing optimal vision and comfort.  Conclusion: Both RGP lenses and spectacles have their benefits and drawbacks. The decision often depends on the individual’s specific visual needs, comfort, and lifestyle preferences. Your ophthalmologist or optometrist can provide personalized recommendations based on the results of the cataract surgery and the child’s visual requirements. | 29.95 | 12.48 | 10.86 |
|  | 9 | The child currently has nystagmus. Will the nystagmus resolve after congenital cataract cataract surgery? | Nystagmus, which is characterized by involuntary, rapid eye movements, may improve, remain the same, or in some cases worsen after congenital cataract surgery. The impact of the surgery on nystagmus depends on various factors:  Potential Outcomes of Congenital Cataract Surgery on Nystagmus: Improvement:  Visual Clarity: If the cataracts were significantly obstructing vision, removing them can improve visual clarity. Enhanced visual input may lead to some reduction in nystagmus, particularly if the nystagmus was partly due to poor visual input. Reduced Visual Deficits: Improved vision can help in stabilizing the visual system, which might lead to a reduction in the intensity of nystagmus. No Change:  Underlying Conditions: If the nystagmus is due to underlying neurological or structural issues that are not related to the cataract, the surgery may not have a significant impact on it. Worsening:  Adaptation Phase: Occasionally, after surgery, there might be a period of visual adaptation where nystagmus could temporarily worsen before improving, especially if the visual system is adjusting to the new input from the corrected vision. Management and Follow-Up: Post-Surgical Monitoring: After cataract surgery, it's essential to monitor the child's vision and nystagmus closely. Regular follow-up appointments with the ophthalmologist are necessary to assess the outcomes and make any required adjustments.  Additional Therapies: If nystagmus persists or remains problematic after surgery, additional therapies may be considered. These can include vision therapy, corrective lenses, or even surgical options depending on the specific case.  Multidisciplinary Approach: In some cases, a multidisciplinary approach involving pediatric ophthalmologists, neurologists, and vision therapists may be needed to address nystagmus comprehensively.  In summary, while cataract surgery can improve visual acuity and potentially have a positive effect on nystagmus, it does not guarantee that nystagmus will resolve. Close monitoring and additional interventions might be required based on the child's specific condition and response to the surgery. | 13.58 | 15.35 | 11.68 |
|  | 10 | We have unilateral congenital cataract in our child. It's said that the surgical outcomes for unilateral cases are not as good. Is this true? | The surgical outcomes for unilateral congenital cataracts can indeed be different from those for bilateral cases, but they are not necessarily worse. Several factors influence the success of surgery and the final visual outcomes:  Factors Affecting Outcomes: Visual Development:  Visual Dominance: In unilateral cases, the eye with the cataract may have been deprived of clear vision for a period, which can affect visual development. If the cataract is removed early, the brain may adapt better, but outcomes can still be influenced by the duration of visual deprivation before surgery. Amblyopia:  Risk of Amblyopia: The eye with the cataract is at risk of developing amblyopia (lazy eye) due to the lack of clear visual input. Successful treatment requires not only cataract removal but also careful management of amblyopia with therapies such as patching or vision therapy. Surgical Technique and Lens Choice:  Intraocular Lens (IOL) Placement: The choice of IOL and the precision of its placement are crucial. In unilateral cases, the focus and alignment of the IOL must be carefully managed to provide the best visual outcome. Rehabilitation:  Post-Surgery Rehabilitation: Post-operative care, including visual rehabilitation and correction with glasses or contact lenses, plays a significant role in achieving the best visual outcome. Outcomes and Expectations: Visual Acuity: With timely and appropriate surgery, many children with unilateral congenital cataracts achieve good visual acuity. However, the final visual outcome can vary based on factors like the age at surgery, presence of amblyopia, and effectiveness of post-surgical therapies.  Adaptation: The brain and visual system can adapt well, especially if surgery is performed early. This adaptation can help in achieving near-normal vision, though some degree of visual disparity between the two eyes may persist.  Conclusion: While unilateral congenital cataracts can present unique challenges, especially concerning visual development and amblyopia, many children achieve excellent outcomes with proper surgical intervention and post-surgical care. The key to success is early diagnosis and treatment, as well as comprehensive follow-up to address any arising issues and support optimal visual development. Consulting with a pediatric ophthalmologist can provide a more personalized outlook based on your child’s specific situation and needs. | 21.25 | 14.13 | 11.39 |
|  |  | After congenital cataract surgery in a child, is it necessary to regularly monitor intraocular pressure and axial length? | Yes, after congenital cataract surgery in a child, regular monitoring of intraocular pressure (IOP) and axial length is important for several reasons:  Monitoring Intraocular Pressure (IOP): Glaucoma Risk: Post-surgical patients, including children, are at risk of developing glaucoma. This condition can be caused by various factors, including inflammation, changes in eye anatomy, or the use of certain medications. Regular IOP checks help in the early detection and management of glaucoma.  Post-Surgical Complications: Elevated IOP can also be a sign of complications such as inflammation or steroid response, which need to be addressed promptly to prevent long-term damage.  Monitoring Axial Length: Growth and Development: In children, the eye continues to grow and develop, even after cataract surgery. Monitoring axial length helps track this growth and ensures that any changes are managed appropriately. This is crucial for adjusting vision correction strategies, such as lenses or glasses.  Lens Power Adjustment: Changes in axial length can affect the power needed for optimal vision correction. Regular measurements help in fine-tuning corrective lenses or determining the need for additional interventions.  Follow-Up Schedule: Post-Surgery Visits: Frequent follow-up visits are essential, especially in the early months and years after surgery. Your ophthalmologist will typically schedule these visits to monitor IOP, axial length, and overall visual development.  Long-Term Care: Ongoing monitoring is crucial as the child grows. The frequency of visits may decrease over time but should be maintained to ensure that any issues are identified and addressed promptly.  Conclusion: Regular monitoring of both intraocular pressure and axial length after congenital cataract surgery is a key part of comprehensive care. It helps manage potential complications, track the child's visual development, and ensure that any necessary adjustments to vision correction are made. Your ophthalmologist will guide you on the appropriate schedule for these evaluations based on your child's specific needs and the details of the surgery. | 29.46 | 12.86 | 10.41 |
|  | 12 | How long after congenital cataract surgery should the child avoid physical education classes? | The duration for which a child should avoid physical education (PE) classes or strenuous activities after congenital cataract surgery can vary based on several factors, including the child’s overall health, the specifics of the surgery, and the ophthalmologist's recommendations. Here’s a general guideline:  Immediate Post-Surgery Period: First Few Weeks: It is typically advised that children avoid vigorous activities and physical education classes for at least 4 to 6 weeks after congenital cataract surgery. This period allows the eye to heal properly and minimizes the risk of complications such as dislodging the intraocular lens (IOL) or causing trauma to the eye.  Initial Healing: During the first few weeks, the focus is on ensuring that the eye heals well. Activities that involve heavy lifting, running, or jumping should be avoided. Gentle activities and a gradual return to physical activity can be considered based on the child’s recovery and the ophthalmologist’s advice.  Follow-Up and Gradual Return: Follow-Up Appointments: Regular follow-up visits with the ophthalmologist will assess the healing progress and determine when it is safe for the child to resume more vigorous activities. The ophthalmologist will provide specific guidelines based on the individual case.  Gradual Resumption: After the initial healing period, physical activities can typically be resumed gradually. The ophthalmologist might recommend starting with less strenuous activities and progressively increasing the intensity as long as there are no signs of complications or issues.  Protective Measures: If the child is involved in sports or activities that pose a risk of eye injury, additional protective measures such as wearing safety goggles or eye shields may be recommended.  Individual Considerations: Surgical Details: The specifics of the surgery, such as whether there were any complications or additional procedures, may affect the recommended period for avoiding physical activities.  Child’s Condition: The child’s overall health, comfort level, and healing progress should also be considered when determining when to resume physical education classes.  Conclusion: While a general guideline suggests avoiding strenuous physical activities for about 4 to 6 weeks after congenital cataract surgery, the best approach is to follow the specific recommendations of your child’s ophthalmologist. They will provide tailored advice based on the child’s surgical outcome and overall recovery. | 22.42 | 14.6 | 10.63 |
|  | 13 | Can a child have a multifocal intraocular lens implanted during congenital cataract surgery, or is a monofocal lens the only option? | The choice between a multifocal intraocular lens (IOL) and a monofocal IOL for congenital cataract surgery in children generally leans towards monofocal lenses, especially in very young children. Here’s why:  Monofocal IOLs: Simplicity and Safety: Monofocal IOLs are the standard choice for children undergoing congenital cataract surgery. They provide a single, fixed focus for either near or distance vision. They are simpler and have a well-established safety profile, which is crucial in the context of pediatric cataract surgery.  Predictability: Monofocal lenses allow for more predictable outcomes and easier post-surgical adjustments. This predictability is important when dealing with the dynamic visual development of children.  Visual Development: In young children, visual development is still ongoing. A monofocal IOL can be set to focus at a distance, and the child can use corrective lenses for near vision as needed. This approach can be more manageable and adaptable as the child grows.  Multifocal IOLs: Complexity: Multifocal IOLs, which provide multiple focal points for near, intermediate, and distance vision, are more complex. They may introduce issues such as reduced contrast sensitivity and visual disturbances, which might be challenging for a child.  Adaptation: Multifocal lenses require a degree of adaptation, and their benefits are generally more suited to adults or older children who have more mature visual systems and a stable visual demand.  Less Common in Pediatrics: Multifocal IOLs are less commonly used in pediatric cases due to the potential for complications and the need for precise visual outcomes. Most pediatric ophthalmologists prefer monofocal lenses for the simplicity and reliability they offer.  Special Considerations: Age and Development: For very young children, who are still undergoing significant visual development, monofocal lenses are usually preferred. For older children, who are closer to full visual maturity, discussions about multifocal lenses might be more relevant, but they are still less common.  Consultation with Specialists: If considering multifocal lenses, it's crucial to consult with a pediatric ophthalmologist who has experience with advanced lens options in children. They can provide guidance based on the child’s specific needs, visual requirements, and overall eye health.  Conclusion: In most cases, a monofocal IOL is the preferred choice for congenital cataract surgery in children due to its predictability and safety. Multifocal IOLs are typically reserved for adult patients or older children with stable visual needs. Discussing your child's specific situation with a pediatric ophthalmologist will help determine the most appropriate lens option for their needs. | 23.46 | 13.82 | 11.25 |
|  | 14 | The severity of congenital cataracts in both of children's eyes is not consistent, should we do cataract surgery in both eyes, or do the severe eye first? | When dealing with congenital cataracts of varying severity in both eyes, the approach to surgery generally involves careful consideration of several factors. Here’s a detailed look at the typical approach:  General Approach to Surgery: Prioritize the Severe Eye:  Immediate Impact: If one eye has a more severe cataract that significantly impairs vision, it is often prioritized for surgery. The rationale is to address the more severe visual impairment first to prevent further damage to visual development. Visual Development: Early intervention in the more severely affected eye can help reduce the risk of developing amblyopia (lazy eye) and improve overall visual outcomes. Assess and Plan for the Second Eye:  Staging: After addressing the more severely affected eye, the next step is to evaluate the less affected eye. This typically involves regular monitoring to assess whether and when intervention might be needed. Timing: The timing of surgery for the second eye can depend on factors such as how the visual development is progressing and whether the less affected eye begins to experience more issues. Factors to Consider: Visual Impairment: The degree to which each cataract affects vision is a critical factor. The more significant the visual impairment in one eye, the higher the priority for surgery.  Age and Development: The age of the child and their visual development stage are crucial. Early surgery in the more severely affected eye is essential to promote normal visual development.  Surgical Risks and Benefits: Each surgery carries risks, and managing these risks is crucial. Performing surgery on the most severely affected eye first allows for better planning and management of the child’s recovery and rehabilitation.  Post-Surgical Monitoring: Close follow-up is necessary to monitor the outcomes of the initial surgery and to determine the best time for addressing the less affected eye. The visual and overall health of the eye should be closely watched.  Consultation and Decision-Making: Pediatric Ophthalmologist: Consult with a pediatric ophthalmologist who can assess the specific situation of your children’s cataracts and recommend a personalized treatment plan. They will consider the severity of each cataract, the potential impact on visual development, and the best approach to achieve optimal outcomes.  Individualized Plan: The decision on whether to operate on both eyes simultaneously or in stages will be based on an individualized plan tailored to the child's needs, taking into account the severity of the cataracts and the overall health of the eyes.  Conclusion: In most cases, the more severely affected eye is prioritized for surgery to address significant visual impairment and reduce the risk of amblyopia. The less affected eye is then monitored and managed accordingly. This staged approach helps in managing risks and optimizing visual outcomes. Your pediatric ophthalmologist will provide the best guidance based on the specific details of your children’s conditions. | 29.34 | 13.12 | 9.96 |
|  | 15 | The congenital cataract surgery has been completed, and now amblyopia training is required. However, all amblyopia training programs increase the duration and intensity of eye use, which could lead to a worsening of myopia. Is this a concern? | Amblyopia training, often referred to as vision therapy or patching, is an essential part of treating amblyopia (lazy eye) after congenital cataract surgery. The concern about worsening myopia (nearsightedness) due to increased eye use during amblyopia training is understandable, but here’s how it can be managed:  Key Points Regarding Amblyopia Training and Myopia: Purpose of Amblyopia Training:  Visual Development: The primary goal of amblyopia training is to improve the visual acuity of the weaker eye and encourage proper visual development. This training often involves activities that promote visual engagement and strengthen the eye's focus and coordination. Myopia Considerations:  Monitoring and Management: While increased use of the eyes can potentially affect refractive errors like myopia, the benefits of amblyopia treatment generally outweigh these risks. Regular monitoring by your ophthalmologist or optometrist can help manage any changes in refractive error. Adjustments: If myopia progression is noted, corrective measures such as adjusting glasses prescriptions or incorporating specialized lenses (e.g., orthokeratology or myopia control lenses) can be considered. Balanced Approach:  Structured Programs: A structured and supervised amblyopia training program helps ensure that the duration and intensity of eye use are appropriate for the child’s condition. This reduces the risk of overuse and helps balance the need for visual stimulation with overall eye health. Individualized Treatment: The amblyopia training plan should be tailored to the child’s specific needs and capabilities. Your healthcare provider will adjust the training as necessary to avoid excessive strain on the eyes. Regular Follow-Up:  Eye Exams: Regular follow-up visits with the ophthalmologist or optometrist are crucial to monitor the progress of amblyopia treatment and any changes in myopia. This allows for timely adjustments to the treatment plan and corrective measures if needed. Additional Measures:  Myopia Control Strategies: If myopia progression becomes a concern, additional strategies might be employed to control its progression, such as myopia management lenses, atropine eye drops, or lifestyle modifications. Conclusion: While there is a potential concern that increased eye use during amblyopia training could impact myopia, this risk is typically managed through careful monitoring and individualized treatment plans. The benefits of amblyopia training in improving visual acuity and development usually outweigh the potential for myopia progression. Regular follow-up with your child’s eye care provider ensures that any issues are promptly addressed and that the treatment plan remains effective and appropriate for your child’s needs. | 20.64 | 14.12 | 11.01 |
| **DG1** | 1 | What are congenital cataracts? | Congenital cataracts refer to cataracts that are either present at birth or develop shortly after due to genetic or developmental abnormalities. This condition is relatively common in children and is one of the leading causes of childhood blindness and vision impairment. | 20.95 | 15.43 | 10.05 |
|  | 2 | What is the most common type of congenital cataract? | The classification of congenital cataracts includes many types based on severity, location of onset, and morphology. Among them, the most common type is perinuclear congenital cataract. | 11.42 | 14.9 | 12.18 |
|  | 3 | Why was I born with cataracts? | The causes of congenital cataracts can be grouped into three main categories: genetic factors, environmental factors, and unknown causes.  1.Genetic Factors: Approximately one-third of congenital cataract cases are due to genetic causes. There are three primary patterns of inheritance: autosomal dominant (AD), autosomal recessive (AR), and X-linked recessive (XR), with autosomal dominant inheritance being the most common. Due to the complexity of genetics, mutations in the same gene can lead to different clinical presentations, and the same clinical presentation can result from mutations in different genes. Currently, congenital cataracts are associated with four main categories of genetic mutations:  (1) Structural abnormalities in lens proteins, leading to improper arrangement of lens fibers (e.g., CRYAA, CRYBA, CRYG).  (2) Changes in membrane proteins within the lens, affecting normal communication between cells and leading to cloudiness (e.g., gap junction protein alpha).  (3) Abnormal expression of regulatory and metabolic proteins involved in lens development (e.g., homeobox genes 3 and 6).  (4) Mutations in heat shock protein 4 (HSF4), which are also linked to cataract formation.  2.Environmental Factors: Congenital cataracts can also be caused by environmental factors, such as:  (1) Viral infections in the mother during the first trimester of pregnancy, including rubella, measles, chickenpox, and mumps, as well as exposure to certain chemicals, X-rays, or poor maternal nutrition during pregnancy.  (2) Premature birth, low birth weight, and prolonged oxygen therapy after birth, all of which can disrupt normal lens development and lead to cataracts.  3.Unknown Causes: In many cases, the exact cause cannot be identified, and these cases are considered sporadic. They are not clearly linked to genetic or environmental factors. | 29.11 | 12.47 | 11.91 |
|  | 4 | What percent of people have congenital cataracts? | Currently, the global prevalence of congenital cataracts is around 4%, while in China it is approximately 0.5%. Among newborns, the incidence is 2.2 to 3 cases per 10,000 births. Worldwide, about 1.5 million children have low vision or are blind due to congenital cataracts. | 43.28 | 9.11 | 9.42 |
|  | 5 | What is the difference between congenital cataract and developmental cataract? | Onset: Congenital cataracts are present at birth or shortly after, whereas developmental cataracts occur later in childhood. Causes: Congenital cataracts are typically caused by prenatal factors or genetic conditions, while developmental cataracts may result from factors occurring after birth or progress over time. Treatment: Both types of cataracts may require surgical intervention and corrective measures, but the timing and approach may vary depending on when the cataract is detected and how much it affects vision. | 16.77 | 17.13 | 12.03 |
|  | 6 | How serious are congenital cataracts? | The severity of congenital cataracts often depends on factors such as which eye is affected, the timing of onset, the type of cataract, the course of the disease, and when treatment is initiated. Mild clouding outside the visual axis typically has little impact on vision. However, if the cloudiness fills the central part of the pupil, it can block light from entering the eye, leading to impaired visual development. Without timely treatment, this can result in amblyopia and may lead to other complications like nystagmus or strabismus. | 36.95 | 13.51 | 9.43 |
|  | 7 | What are the long term effects of congenital cataracts? | The long-term effects of congenital cataracts depend on the severity of the condition and the timing of treatment. In general, mild cataracts have minimal long-term impact, with children typically maintaining good vision and being less likely to develop complications. If the cataracts are more severe but detected and treated early, the long-term effects are usually manageable, and most children can achieve normal visual development through corrective measures and visual training. However, if treatment is delayed, it can lead to significant visual impairment that may be difficult to correct, and complications such as strabismus or nystagmus may occur. | 24.1 | 16.11 | 10.09 |
|  | 8 | Does congenital cataract cause amblyopia? | Congenital cataracts can indeed lead to amblyopia. The clouding of the lens interferes with light entering the eye, which disrupts normal visual development in children. If both eyes are severely affected and treatment is delayed, the risk of developing amblyopia increases, and the child may experience worsening vision along with complications like strabismus or nystagmus . In cases where only one eye is affected and the other eye has a clear lens, even with timely surgery and no complications, the affected eye can still develop amblyopia if post-surgical treatment is not aggressive enough. This is because the brain tends to rely on the eye with better vision, leading to visual development problems in the weaker eye, a condition known as deprivation amblyopia. | 30.21 | 15.11 | 10.41 |
|  | 9 | What is the minimum age for congenital cataract surgery? | There is no strict minimum age for congenital cataract surgery, as the timing depends on the child’s overall health, the surgeon’s expertise, and the anesthesia requirements. Doctors typically aim to perform surgery during the critical period of visual development to maximize the potential for vision recovery. For one eye congenital cataracts, surgery is generally recommended within 4 to 6 weeks after birth. For bilateral cataracts, surgery can be delayed slightly. The timing is based on factors such as the type and location of the lens clouding, how much it obstructs light from entering the eye, and the development of the eye’s axial length and corneal curvature. | 27.91 | 14.53 | 10.59 |
|  | 10 | What happens if congenital cataracts are left untreated? | If congenital cataracts go untreated, they can lead to a range of serious consequences. First, the cloudiness of the lens obstructs light from entering the eye and focusing on the retina, which can significantly impair visual clarity and result in severe vision loss. Additionally, because the visual system develops rapidly in the first few years of life, the lack of clear visual input can cause the brain to favor the eye with better vision. This can hinder the visual development of the affected eye, leading to amblyopia. In more severe cases, untreated cataracts may also result in complications such as strabismus and nystagmus. | 38.08 | 13.07 | 9.87 |
|  | 11 | How successful is congenital cataract surgery? | The success rate of congenital cataract surgery depends on various factors, including the timing of the procedure, the child's age, the type and severity of the cataract, and whether the child has any other congenital abnormalities affecting the eyes. The surgeon's clinical experience and the quality of postoperative care and visual rehabilitation training also play crucial roles. The primary goal of the surgery is to restore the child's vision and prevent the development of amblyopia. However, a successful surgery does not guarantee that the child's postoperative vision and visual development will be the same as that of children without cataracts. Professional visual rehabilitation training and careful postoperative care are essential to maximize the surgical outcome. | 13.55 | 17.08 | 11.09 |
|  | 12 | What is the most common complication of congenital cataract surgery? | Currently, the most common complications following congenital cataract surgery are: Visual Axis Opacification (VAO): Younger patients are at a greater risk for developing VAO, which results from the regeneration of lens cortical material and leads to secondary opacification of the visual axis. Research indicates that VAO occurs in over 95% of children. Postoperative Elevated Intraocular Pressure (IOP): This complication is more common in children who have surgery without an intraocular lens, with younger patients facing a higher risk. | 12.6 | 16.28 | 13.41 |
|  | 13 | What is the surgery of choice for congenital cataract? | Surgeons will choose different surgical approaches based on the child's age:  For children under 2 years old: The preferred method is simple cataract extraction, followed by a secondary intraocular lens (IOL) implantation at a later stage.  For children over 2 years old: The typical procedure involves phacoemulsification cataract extraction, IOL implantation, posterior capsule removal, and anterior vitrectomy.  For children aged 5 to 8 years: The decision to perform posterior capsule removal and anterior vitrectomy will be made based on preoperative evaluations, the child's ability to cooperate during examinations, and their adherence to follow-up care. | 12.68 | 17.14 | 11.19 |
|  | 14 | How are congenital cataracts optically corrected? | After congenital cataract removal surgery, optical correction methods primarily include:  Glasses: They are relatively inexpensive, easy to fit, and can be changed as needed. However, children without a lens in one eye may experience discomfort due to differences in vision between the two eyes.  Contact Lenses: These include soft contact lenses and rigid gas-permeable (RGP) lenses. Contact lenses provide better visual quality and a more natural appearance, making them particularly suitable for patients with unilateral cataracts or those who cannot receive an intraocular lens. However, they tend to be more costly and require strict adherence to usage and care guidelines. | 28.09 | 13.52 | 11.19 |
|  | 15 | Is congenital cataract progressive? | Most congenital cataracts are present at birth and do not progress as the child grows. However, some developmental cataracts may not initially affect vision despite early lens opacification. In these cases, doctors often recommend regular follow-up and observation. As the child matures, metabolic changes may lead to increased lens clouding, resulting in what is known as adult "developmental cataracts". | 29.46 | 12.88 | 10.43 |
| **DD1** | 1 | Will the vision improve after congenital cataract surgery? | Many parents of children with congenital cataracts are understandably concerned about whether their child's vision will significantly improve after surgery. Today, I want to give you a detailed rundown on the surgical outcomes for congenital cataracts. As you know, the main goal of cataract surgery is to remove the cloudy lens and then implant a clear IOL. For children who aren't candidates for IOL, we correct vision with contact lenses or glasses. But here are a few key points to keep in mind for the best postoperative results.  Early Intervention: The earlier we perform the surgery, the better the outcome for vision improvement. Early intervention is crucial for the normal development of the visual pathway and reduces the risk of amblyopia. However, performing surgery too early can increase the risk of secondary glaucoma and visual axis opacification.  Postoperative Visual Rehabilitation: After cataract surgery, vision improvement in children is different from that in adults. It requires a dedicated visual rehabilitation program to effectively enhance vision. The brain needs time to adapt to the new visual input, especially in very young children, making regular rehabilitation and follow-ups essential.  Visual Prognosis Influenced by Various Factors: While vision generally improves to some extent after surgery for all children with congenital cataracts, the final visual outcome can vary due to several factors. These include the morphology of the cataract, the severity of obstruction in the visual axis, the presence of other congenital eye diseases, the timing of the surgery, potential surgical complications, the frequency and duration of postoperative rehabilitation, and whether it's unilateral or bilateral cataracts. Therefore, long-term ophthalmic follow-ups are vital for children with congenital cataracts. | 19.24 | 15.46 | 11.04 |
|  | 2 | If an IOL is implanted now, will it need to be replaced when the individual reaches adulthood? | In most cases, the IOLimplanted during the first cataract surgery for babies with congenital cataracts are designed to last a lifetime. They're made to be durable and serve the child's vision needs as they grow. However, there are a few considerations to keep in mind:  Changes in IOL Power: If a child's eye axis continues to grow significantly after the IOL is implanted, the power of the IOL might not be optimal when they reach adulthood. This could potentially require a replacement or adjustment of the IOL later in life, but this is not very common.  Long-term Complications with the IOL: If there's displacement, opacification, or other issues with the implanted lens, it might need to be replaced or adjusted. These situations are quite rare, but they do happen.  So, regular check-ups are essential to ensure that the IOL continues to meet the child's visual needs and to address any potential issues that may arise. | 51.12 | 11 | 9.14 |
|  | 3 | Is the congenital cataract surgery risky? | Congenital cataract surgery is generally safe and effective, but like any surgery, it comes with some risks and potential complications. Let's go over them:  1.Infection: Just like with any surgery, there's a risk of infection. But don't worry, modern minimally invasive techniques and sterile procedures, along with antibiotics used before and after surgery, have really brought that risk down.  2.Sterile Inflammation: Kids can have a more noticeable inflammatory response after cataract surgery compared to adults. If the surgery takes a long time or if there's additional surgery on the iris, the reaction can be even stronger. That's why using steroids after surgery is a must. To avoid a severe inflammatory response, it's crucial to have the surgery done at a specialized eye hospital by a doctor with lots of experience in pediatric cataract surgery.  3.Secondary Glaucoma: Sometimes after surgery, there can be a temporary increase in eye pressure. That's why we check the eye pressure at every follow-up visit, and treat it if necessary, to prevent damage to the optic nerve from high pressure.  4.IOL Malposition or Dislocation: If the cataract surgery isn't perfect, it can lead to the intraocular lens (IOL) being stuck or misaligned. Long-term misalignment can even cause the IOL to shift out of place.  Now, let's talk about the benefits and things to keep in mind:  1.Vision Improvement: The biggest benefit of congenital cataract surgery is improving vision in children during their critical period of visual development, helping to prevent or reduce amblyopia caused by visual deprivation from the cataract.  2.Early Intervention: Getting the surgery early can help prevent or minimize the impact of the cataract on the development of the visual cortex, reducing the risk of complications like amblyopia.  3.Long-term Monitoring: Regular follow-ups are essential to ensure long-term vision improvement.  In summary, congenital cataract surgery is a proven and effective procedure with a high success rate. While the risks can't be completely avoided like they can with adult cataract surgery, parents of children with congenital cataracts should discuss the potential risks and benefits with their ophthalmologist before the surgery. This will help make an informed decision and prepare for the postoperative recovery process. | 33.39 | 13.35 | 10.41 |
|  | 4 | Do we have to proceed with the congenital cataract surgery now, or can we wait? | Parents of babies with congenital cataracts have to decide whether to go ahead with surgery right away or wait, and there are a few key things to think about: (1) The severity of the cataracts: The need for surgery is based on how bad the cataracts are and how much they affect vision. If it's a total white cataract or a posterior subcapsular cataract in the visual axis, not treating it in time could lead to amblyopia or low vision. If the cataracts aren't significantly harming vision or causing other issues, I'd suggest regular check-ups and elective surgery if needed. (2) Visual development: Early intervention is crucial for proper visual development. The visual pathways in a young child's brain are still developing, and timely surgery can reduce the risk of deprivation amblyopia and abnormal visual development caused by the cataracts. (3) Preventing complications: Delaying treatment for cataracts can lead to other complications, such as strabismus, nystagmus, and poor stereopsis, which are visual dysfunctions. (4) Age and overall health: The child's overall health and readiness for general anesthesia are important. If the baby is too young or has other health concerns, we might recommend waiting. In the end, I advise parents to carefully weigh the pros and cons when making the decision for surgery. If you decide to wait, regular eye exams to monitor the cataracts and ensure they're not negatively affecting your child's vision are essential. | 35.38 | 13.59 | 9.63 |
|  | 5 | Will congenital cataract redevelop after the surgery? | If congenital cataract surgery in children and infants doesn't involve removing the posterior capsule, there's a 100% chance of developing posterior capsule opacification (PCO), also known as Visual Axis Opacification (VOA). So, how can we prevent or avoid the occurrence of PCO or VOA? Here are some important considerations to keep in mind:  (1) The younger the child, the more likely they are to develop VOA. Surgeons should aim to remove as much peripheral cortex as possible during surgery for children under six and ensure that the posterior capsule and anterior vitreous are removed with a sufficient diameter.  (2) For children over six years old with congenital cataracts, the surgery can involve a simple posterior capsule tear during the procedure or, in the first few months after cataract removal when the child is more cooperative, a relatively straightforward outpatient procedure known as YAG laser posterior capsulotomy.  (3)Regular follow-ups: Even with a successful cataract surgery where the posterior capsule and anterior vitreous are adequately removed, some children may still develop PCO or VOA. It's essential to have regular eye exams post-surgery, and if VOA or PCO occurs, a secondary posterior capsulotomy may be necessary. | 24.73 | 15.77 | 11.24 |
|  | 6 | Can we continue with amblyopia training and delay the congenital cataract surgery for a few years? | Although amblyopia training is a crucial part of managing vision development, if cataracts have already caused opacification in the visual axis, leading to deprivation amblyopia, regular amblyopia training cannot replace the need for congenital cataract surgery. Why do we say that? Let's analyze the importance of early surgery for congenital cataracts:    (1) Visual development: For infants and young children, the main goal of cataract surgery is to prevent or reduce vision loss. Cloudy lenses can interfere with the development of the brain's visual pathways. Removing the cataracts early on helps ensure stable vision improvement and reduces the risk of severe amblyopia.    (2) Preventing amblyopia: Congenital cataracts, especially in one eye, can lead to amblyopia if left untreated. Amblyopia treatments, such as glasses, patching the sound eye, and other therapies, are more effective the earlier they are started. Training after cataract removal yields much better results than before removal.    (3) Risks of delayed surgery: Progressive congenital cataracts can lead to ongoing vision loss and increase the difficulty of amblyopia training if surgery is delayed. The longer the cataract affects vision, the harder it is to improve it, even with ongoing amblyopia training.    (4) Complications: Untreated cataracts can lead to other complications, such as strabismus, nystagmus, and poor stereopsis, which are visual dysfunctions that complicate later treatment and management.    It's clear that early surgery is the best way to prevent complications from congenital cataracts and provides the best foundation for vision recovery. | 21.72 | 14.74 | 11.03 |
|  | 7 | Our eldest child has bilateral congenital cataracts, and we are considering having a second child. What is the likelihood of the second child developing cataracts? | The likelihood of a child having congenital cataracts can be influenced by a variety of factors, including genetic and environmental factors.    (1) Let's talk about genetic factors first: If your first child has congenital cataracts due to genetic reasons, it might increase the risk for your second child to develop cataracts as well. Some hereditary conditions related to congenital cataracts can be passed down from parents to their children. In such cases, it's recommended to undergo genetic testing to more accurately assess the risk for your second child to have cataracts.    (2) Environmental and other factors: Non-genetic causes: Congenital cataracts can also be caused by non-genetic factors, such as prenatal infections (like rubella) or exposure to certain substances during pregnancy. If your eldest child's cataracts were due to these factors, the risk for your second child might be reduced. Maintaining good prenatal care and avoiding known risks is important. Make sure you receive regular prenatal care to monitor the health of your pregnancy and address any potential cataract risks.    Overall, if congenital cataracts are caused by genetic factors, then each pregnancy might carry a higher risk. Getting timely prenatal genetic testing, consulting with a genetic specialist, and obtaining the most accurate information can help parents make informed decisions. | 30.76 | 14.14 | 10.67 |
|  | 8 | After congenital cataract surgery, is it better to wear rigid gas-permeable (RGP) lenses or regular spectacles? | After a congenital cataract extraction, choosing the right type of eyewear for an aphakic eye can be a challenge. The decision between rigid gas-permeable (RGP) contact lenses and regular spectacles depends on several factors, including the patient's specific needs, the visual quality of an intraocular lens (IOL) if used, and the comfort of the baby's eyes. Let's compare the two to help you understand:  Advantages of RGP lenses: (1) They can provide undistorted good vision, especially if there is an irregular cornea or high hyperopia that cannot be fully corrected by spectacles. (2) Stability: RGP offers a stable and precise optical correction, even for irregular astigmatism.  Disadvantages: (1) Comfort: They may not be as comfortable as soft contact lenses or glasses, especially for babies who are not used to wearing them. (2) Adaptation: It might take some time for the child to get used to wearing RGP lenses.  Advantages of regular spectacles: (1) Ease of use: Glasses are easy to use, do not require direct contact with the eyes, and are universally applicable. (2) Comfort: Some babies may find glasses more comfortable than contact lenses. (3) Less maintenance: They are easier to clean and maintain than contact lenses.  Disadvantages: (1) Limited vision correction: Spectacles cannot correct high hyperopia in aphakic eyes as effectively as contact lenses, especially when combined with irregular astigmatism. (2) Peripheral vision: Glasses do not provide as wide a peripheral field of view as RGP lenses.  In summary, RGP lenses and regular spectacles each have their pros and cons. An ophthalmologist or optometrist can provide personalized advice based on the results of the cataract surgery and the child's visual requirements. If considering the child's comfort and a simple, harmless lifestyle, spectacles might be preferred over RGP. Regular follow-ups with an ophthalmologist or optometrist are crucial to ensure that the chosen correction method provides the best vision and comfort. | 35.29 | 12.11 | 10.26 |
|  | 9 | The child currently has nystagmus. Will the nystagmus resolve after congenital cataract cataract surgery? | Nystagmus refers to an involuntary rapid eye movement, and its presence after congenital cataract surgery may improve, remain the same, or even worsen. The impact of surgery on nystagmus depends on various factors:  (1) Improving visual clarity: If the cataract severely impedes vision, removing it can enhance visual clarity. Enhanced visual input may reduce nystagmus to some extent, especially if the primary cause of the nystagmus is poor visual input.  (2) Reducing visual disability: Improved vision can help stabilize the visual system, which may lead to a decrease in the intensity of nystagmus.  (3) Reasons for unchanged nystagmus: If the nystagmus is due to underlying neurological or structural issues unrelated to the cataract, the surgery may not affect it.  Close monitoring of the child's vision and nystagmus after cataract surgery is essential. If nystagmus persists, surgical treatment may be considered based on the specific circumstances. It requires a collaborative effort from pediatric ophthalmologists, neurologists, and strabismus specialists to address nystagmus.  In summary, while congenital cataract surgery can improve vision and potentially have a positive effect on nystagmus, it does not guarantee the complete resolution of nystagmus. Depending on the specific circumstances of the child with congenital cataracts and their response to surgery, other interventions may be necessary. | 13.83 | 15.94 | 12.14 |
|  | 10 | We have unilateral congenital cataract in our child. It's said that the surgical outcomes for unilateral cases are not as good. Is this true? | The surgical outcomes for congenital cataracts in one eye are indeed different from those in both eyes. The reason is that a cataract in one eye deprives it of clear vision for a certain period after birth, affecting the development of the visual nerves on that side. Meanwhile, the healthy eye on the opposite side develops normally, leading the child to rely primarily on the healthy eye for visual tasks and the transmission of visual information before cataract surgery and amblyopia training. As a result, the eye with the cataract is highly likely to develop amblyopia. Successful treatment requires not only the removal of the cataract but also the use of forced visual training by patching the healthy eye. This encourages the brain and visual system to adapt well, especially if the surgery is performed early. This adaptation can help achieve near-normal vision, although some degree of visual difference between the two eyes may persist.  Therefore, with timely cataract surgery and continuous, proper patching for amblyopia training, many children with unilateral congenital cataracts can achieve good vision. However, the final visual outcomes may vary depending on factors such as the age at the time of surgery, the presence of amblyopia, and the effectiveness of postoperative treatment. Unilateral congenital cataracts present unique challenges in terms of visual development and amblyopia. The key to success lies in early diagnosis and treatment, followed by persistent postoperative patching for amblyopia training. | 25.98 | 15.01 | 10.42 |
|  | 11 | After congenital cataract surgery in a child, is it necessary to regularly monitor intraocular pressure and axial length? | Regular postoperative checks of intraocular pressure (IOP) and eye axial length in children with congenital cataracts are crucial.  (1) Regular IOP monitoring is vital due to the 3-5% risk of secondary glaucoma after surgery. Factors like inflammation, anatomical changes, and corticosteroid use can play a role. These checks aid in early glaucoma detection and management.  (2) Axial length monitoring is important as it grows with the child, sometimes faster after cataract surgery. It helps track refractive error changes and adjust correction strategies like lens replacements or glasses. Follow-ups at 1 week, 1 month, 3 months, 6 months, and yearly are typical, aiding in managing long-term post-surgical complications and ensuring proper vision correction. | 27.46 | 13.26 | 10.4 |
|  | 12 | How long after congenital cataract surgery should the child avoid physical education classes? | The answer to this question depends on several factors, including the overall eye health of the child and the complexity of the cataract surgery. As a general guideline, it's recommended that children with congenital cataracts avoid strenuous exercise and physical education classes for at least 4 to 6 weeks after cataract surgery. This period allows the eye to heal properly and minimizes the risk of surgical complications, such as displacement of the intraocular lens, infection within the eye, dehiscence of the incision, or injury to the eye. Additionally, activities involving weightlifting, running, or jumping should be avoided.  Consider engaging in some gentle activities to gradually regain strength. Regular follow-ups with an eye doctor are essential to assess the progress of the surgical wound healing. If the child participates in sports or activities that could potentially cause eye injury, it is advisable to take extra protective measures, such as wearing safety goggles or eye shields. | 25.4 | 15.15 | 10.19 |
|  | 13 | Can a child have a multifocal intraocular lens implanted during congenital cataract surgery, or is a monofocal lens the only option? | Selecting an IOL for kids' cataract surgery often favors single-focus IOLs, ideal for young children. These IOLs are simple, safe, and maintain post-op contrast sensitivity, fitting most children with cataracts and amblyopia. Their predictability aids in post-op refractive adjustments, vital for children's changing vision.  In young kids' visual development, single-focus IOLs set for distance let them use near-vision correction as needed, keeping some hyperopia. This is manageable as their eye axis grows. Multifocal IOLs, offering various focal points, might reduce contrast and cause visual issues, less suitable for post-cataract amblyopia in kids. They fit adults better with stable visual needs.  So, single-focus IOLs are preferred for pediatric cataract surgery. | 38.96 | 11.43 | 10.48 |
|  | 14 | The severity of congenital cataracts in both of children's eyes is not consistent, should we do cataract surgery in both eyes, or do the severe eye first? | When addressing congenital cataracts of varying severities in both eyes, the timing of surgery and the eye to operate on first typically require careful consideration of the following factors:  Prioritize the eye with the more severe cataract. If one eye has a significantly opaque lens causing greater visual impairment, it often requires surgery first to prevent further damage to visual development. Early intervention in the more severely affected eye can help reduce the risk of developing amblyopia.  After addressing the more severely affected cataract, the next step is to assess how to manage the less affected eye. The timing of the second eye's surgery depends on factors such as the state of visual development and whether the less affected eye is beginning to exhibit more visual issues. The child's age and their stage of visual development are crucial. Early surgery is vital for promoting normal visual development. However, the risks associated with pediatric cataract surgery are higher than in adults, so managing these risks is also essential. Choosing an optimal surgery time for the less affected eye with cataract is important. Whether to perform surgery on both eyes simultaneously or in stages, a personalized plan suitable for pediatric bilateral cataracts should be determined.  In conclusion: In most cases, the more severely affected eye is prioritized for surgery to address significant visual impairment and reduce the risk of amblyopia. Then, close monitoring and appropriate management measures are taken for the less affected cataract eye, which helps to manage risks and optimize visual outcomes. | 24.34 | 14.68 | 10.24 |
|  | 15 | The congenital cataract surgery has been completed, and now amblyopia training is required. However, all amblyopia training programs increase the duration and intensity of eye use, which could lead to a worsening of myopia. Is this a concern? | Amblyopia therapy is key after congenital cataract surgery. It's a common worry that this therapy could increase myopia, but it's manageable. The goal is to boost vision in the weaker eye and support visual growth. Activities focus on visual engagement and eye coordination.    Increased eye use might affect refractive errors, but the benefits of treatment are significant. Eye care professionals track refractive changes. If myopia progresses after surgery, strategies like lens adjustments, special lenses, atropine eye drops, or lifestyle changes can help.    Treatment should be personalized. Training intensity varies with the severity of amblyopia to avoid eye strain.    In summary, while amblyopia training could potentially lead to myopia, this risk is controlled with monitoring and individualized plans. The benefits of improved vision and development from amblyopia training usually outweigh the myopia risks. | 26.74 | 12.51 | 11.53 |
| **DG2** | 1 | What are congenital cataracts? | Congenital cataracts are a unique kind we're talking about. They pop up right after a baby's born or within their first year. It's possible that they're linked to genetic factors, infections that happen in the womb, or even not getting enough nutrients while the baby's growing inside the mom. | 64.22 | 8.36 | 7.99 |
|  | 2 | What is the most common type of congenital cataract? | When we're talking about the different looks of congenital cataracts, the most common one we see is called a lamellar cataract. It's the most, making up about 34% of the cases. | 59.07 | 8.75 | 7.01 |
|  | 3 | Why was I born with cataracts? | This could be due to a few things happening during pregnancy. It might be about our genes, little changes in them we call mutations. Or maybe infections that happen in the womb, like from viruses named rubella or cytomegalovirus. Also, not getting the right nutrients can play a part too. | 65.56 | 7.22 | 7.1 |
|  | 4 | What percent of people have congenital cataracts? | The incidence rate of congenital cataracts is estimated to be between 12 to 136 per 100,000 newborns. | 35.51 | 11.78 | 11.1 |
|  | 5 | What is the difference between congenital cataract and developmental cataract? | (a) Different reasons: Developmental cataracts often come from genes, the world around us, or not eating enough good food messing with how the lens in our eye works. Congenital cataracts usually happen because something went wrong as we were growing in the womb, mom getting sick, or how our body processes things. (b) Different signs: People with developmental cataracts might see less clear, things might look blurry, or their eyes might not look straight ahead. Those with congenital cataracts might see very poorly, have shaky eyes, or their eyes might not line up right. (c) Different outcomes: Developmental cataracts usually don't mess with our sight too much, and if we get help soon, we can see fine later on. But congenital cataracts, if we don't get help quick, can make it hard to see and sometimes even lead to lazy eyes or not seeing at all. | 52.34 | 8.79 | 7.66 |
|  | 6 | How serious are congenital cataracts? | If not treated promptly, congenital cataracts can affect vision and may even lead to amblyopia and blindness in severe cases. | 34.26 | 13.45 | 9.37 |
|  | 7 | What are the long term effects of congenital cataracts? | The long-term impact of congenital cataracts is mainly about the drop in vision or the lack of improvement in sight, which is all about catching it early, getting it treated right away, and following up with visual training. If we can get the surgery done in time, patients need to keep an eye on their vision, the growth of their eye axis, and the development of their visual functions over the long haul, and they might need to do some amblyopia training too. But if we don't catch it and treat it early, it can lead to a decline in vision, a halt in eye growth, and in the long run, it might cause amblyopia or even blindness. | 48.56 | 16.34 | 8.26 |
|  | 8 | Does congenital cataract cause amblyopia? | Congenital cataracts can easily lead to amblyopia, but if we catch it early and get the surgery done, along with follow-up amblyopia training, some kids can regain normal vision. But if it's not treated promptly and correctly, there's a good chance it could develop into amblyopia. | 40.78 | 13.41 | 9.17 |
|  | 9 | What is the minimum age for congenital cataract surgery? | The timing for surgery depends on how severe the cataracts are and the overall health of the child. For unilateral, dense cataracts, we can operate around 4 to 6 weeks after birth. For bilateral, dense cataracts, we might wait until 6 to 8 weeks after birth. But for the less severe cases, we can adjust the surgery time according to the child's specific situation and the degree of the cataracts. | 52.93 | 9.98 | 8.28 |
|  | 10 | What happens if congenital cataracts are left untreated? | If not treated promptly, it will lead to a decrease in vision, cessation of eyeball development, and in the long run, may result in amblyopia or even blindness. | 42.45 | 14.29 | 9.54 |
|  | 11 | How successful is congenital cataract surgery? | The success of cataract surgery really hinges on a few key things: the surgeon's experience, the right timing for the operation, how bad the cataracts are, the kid's age, and their overall health. Fortunately, we've got a high success rate with congenital cataract surgeries these days. We make sure to do a complete work-up before the surgery to give it the best shot at going well. | 54.11 | 11.26 | 8.04 |
|  | 12 | What is the most common complication of congenital cataract surgery? | In congenital cataract surgery, the most common hitches we see are clouding up of the visual axis, which includes what we call posterior capsule opacification (PCO) and secondary membrane formation. It's pretty much a given with kids born with cataracts, especially the little ones. When we operate and leave the back part of the lens capsule whole, this can happen. To handle it, we often wait until the kid is at a certain age, then we might do a laser treatment or sometimes we've got to go in there and do a second surgery. | 51.58 | 11.9 | 8.5 |
|  | 13 | What is the surgery of choice for congenital cataract? | Depending on the age of the child, we tailor the surgical approach. For kids under 2, we go with a straightforward cataract removal, and then later on, we do a secondary IOL implantation. For those older than 2, the plan is to do phacoemulsification to remove the cataract, pop in an intraocular lens, and also take out the posterior capsule and the anterior vitreous. For kids aged 5 to 8, we look at how well they can cooperate during the pre-op exams and how likely they are to stick with follow-up care, and based on that, we make a call on whether to do a posterior capsulotomy and anterior vitrectomy. | 47.83 | 13.3 | 8.78 |
|  | 14 | How are congenital cataracts optically corrected? | RGP lenses and glasses can both be used. But because RGP lenses do a great job of minimizing optical aberrations and distortions, which means better image quality, and because they promote the development of single binocular vision and fusion functions, and can improve visual quality for kids with nystagmus, RGP is really the preferred method for correcting vision after congenital cataract surgery. | 21.18 | 18.01 | 11.8 |
|  | 15 | Is congenital cataract progressive? | Usually, congenital cataracts are there right from birth and don't get worse as a child grows. But, if treatment is delayed, the vision problems from congenital cataracts can get more serious over time, and they might cause other issues like strabismus. | 53.97 | 10.82 | 9.27 |
| **DD2** | 1 | Will the vision improve after congenital cataract surgery? | After the surgery, most kids, with the help of some vision exercises or wearing glasses right on time, can see a lot better. | 62.11 | 10.31 | 6.84 |
|  | 2 | If an IOL is implanted now, will it need to be replaced when the individual reaches adulthood? | Right now, the majority of kids don't need a lens implant again. Before we go for an IOL, we take into account factors like potential impact on vision, eye axis development, and refractive shifts to choose the right one. In some cases, if there's a significant mismatch in refractive power that's hard to correct, a replacement might be necessary. | 53.02 | 10.67 | 8.95 |
|  | 3 | Is the congenital cataract surgery risky? | The risks of surgery for congenital cataracts really hinge on the severity of the child's condition, their overall health, how they handle anesthesia, the skill level of the surgeon, and the timing of the surgery. | 23.86 | 18.63 | 9.88 |
|  | 4 | Do we have to proceed with the congenital cataract surgery now, or can we wait? | The timing for surgery really depends on how severe the cataracts are and the child's overall health. For single eye, dense cataracts, we can operate around 4 to 6 weeks after birth; for both eyes with dense cataracts, we typically do the surgery between 6 to 8 weeks after birth. With less severe cataracts, we can adjust the surgery time based on the child's specific condition and how serious it is. Plus, whether we go for surgery or not, we recommend that patients get prompt visual training, which can help improve their best possible vision. On the flip side, if we delay surgery, the affected eye can develop amblyopia due to deprivation of visual input, and the later we treat it, the harder it is to boost the vision. | 49.48 | 12.57 | 9.3 |
|  | 5 | Will congenital cataract redevelop after the surgery? | Some kids have leftover lens epithelial cells on the capsule that can grow and move, causing a secondary cataract and leading to a drop in vision. For cooperative kids, we can do a YAG laser capsulotomy right in the clinic; for those who have a hard time cooperating, we might opt for a surgical removal of the posterior capsule along with anterior vitrectomy. | 36.55 | 15.99 | 9.96 |
|  | 6 | Can we continue with amblyopia training and delay the congenital cataract surgery for a few years? | The main reason children with congenital cataract get amblyopia is because their eyes don't get the right kind of stimulation they need to grow properly. The cataract blocks the view, so the eye doesn't develop like it should. To really give these kids a chance to see better, we've got to fix this lack of stimulation at the root. | 64.99 | 9.08 | 7.29 |
|  | 7 | Our eldest child has bilateral congenital cataracts, and we are considering having a second child. What is the likelihood of the second child developing cataracts? | Congenital cataracts stem from a mix of genetic and environmental factors. Studies suggest that about 10% to 25% of congenital cataracts are inherited. If a couple's first child has congenital cataracts due to genetic causes, there's a higher likelihood that a second child could develop them too. Also, if the parents themselves have congenital cataracts, their offspring are at an increased risk of having it. | 39.11 | 11.72 | 9.93 |
|  | 8 | After congenital cataract surgery, is it better to wear rigid gas-permeable (RGP) lenses or regular spectacles? | So, both have their pros and cons. RGP lenses do a great job of minimizing optical aberrations and distortions, giving us crisp image quality; they also promote the development of binocular vision and fusion functions; and they can improve visual quality for kids with nystagmus. The downside is they can be pricey and a bit of a hassle to handle. If not worn or cared for properly, there's a risk of corneal or conjunctival damage or infection. On the other hand, glasses are easy to wear and cost-effective, and they're hygienic. But they've got their drawbacks too, like peripheral image distortion, a reduction in retinal image size, and a narrower peripheral field of view. For kids without an intraocular lens: if it's a single eye, we'd go for contact lenses as the first choice; if it's both eyes, we can consider either contacts or glasses. For those with an intraocular lens, glasses can do the trick. And if glasses aren't suitable, contact lenses might be the way to go. | 56.78 | 10.03 | 9.12 |
|  | 9 | The child currently has nystagmus. Will the nystagmus resolve after congenital cataract cataract surgery? | Nystagmus can have a variety of causes. The kind that's due to cataracts messing with the development of fixation can improve to some extent after cataract surgery when visual function gets better. Some kids might even see it disappear completely. | 45.25 | 10.26 | 9.03 |
|  | 10 | We have unilateral congenital cataract in our child. It's said that the surgical outcomes for unilateral cases are not as good. Is this true? | Unilateral congenital cataracts tend to have a higher risk of amblyopia post-surgery, and the improvement in vision from treatment isn't always as marked as with bilateral cases. However, identifying it early, getting it taken care of with surgery, and sticking with amblyopia training can really optimize the surgical results for these kids. | 20.31 | 17.01 | 10.02 |
|  | 11 | After congenital cataract surgery in a child, is it necessary to regularly monitor intraocular pressure and axial length? | After surgery, it's important to regularly measure the axial length and intraocular pressure. On one hand, there's a higher chance of increased eye pressure after congenital cataract surgery, and keeping an eye on it helps prevent post-op complications. On the other hand, the growth of the axial length is a pretty objective measure of how the eye is developing, and it needs to be monitored regularly to keep track of ocular growth. This way, we can make timely adjustments to the kid's refractive correction plan. | 45.32 | 12.28 | 9.11 |
|  | 12 | How long after congenital cataract surgery should the child avoid physical education classes? | About three monthes. | 90.99 | 1.31 | 0.15 |
|  | 13 | Can a child have a multifocal intraocular lens implanted during congenital cataract surgery, or is a monofocal lens the only option? | 1.Most kids with congenital cataracts have amblyopia, so they're not really good candidates for multifocal IOLs; 2.The axial length of a child's eye can still change before they turn 18, and post-surgery refractive changes can significantly reduce the effectiveness of multifocal IOLs, and might even increase the risk of amblyopia. So, we generally don't recommend implanting multifocal IOLs in children's eyes. | 30.87 | 14.01 | 10.08 |
|  | 14 | The severity of congenital cataracts in both of children's eyes is not consistent, should we do cataract surgery in both eyes, or do the severe eye first? | Our surgical planning takes a variety of factors into account. If the child is young and has bilateral cataracts that are relatively severe, we'd recommend simultaneous cataract removal in both eyes, followed by secondary IOL implantation at a later stage. If the child is older and the cataracts vary in severity between the eyes, we'd opt to operate on the more severely affected eye first. | 31.26 | 14.28 | 9.33 |
|  | 15 | The congenital cataract surgery has been completed, and now amblyopia training is required. However, all amblyopia training programs increase the duration and intensity of eye use, which could lead to a worsening of myopia. Is this a concern? | Theoretically, vision training, because it enhances visual stimulation, could lead to an increase in myopia in children. But without amblyopia training, the child's corrected vision won't improve, which could negatively impact their long-term visual development. It's recommended to pay attention to eye posture during amblyopia training, maintain a good amount of outdoor activity, and regularly check the refractive status to try to prevent the progression of myopia. | 17.12 | 16.5 | 10.33 |
| **RE-CG** | 1 | What are congenital cataracts? | Congenital cataracts happen when a baby is born with a cloudy lens in the eye. This cloudiness can make it hard to see clearly. The lens is the part of the eye that helps focus light so we can see well. When the lens is cloudy, light can’t pass through easily, which makes vision blurry. | 82.13 | 5.22 | 7.19 |
|  | 2 | What is the most common type of congenital cataract? | The most common type of congenital cataract is called a nuclear cataract. This type affects the middle part of the lens in the eye. Nuclear cataracts make the center of the lens cloudy, which can cause blurry vision.  Key Points About Nuclear Cataracts: Appearance: Nuclear cataracts look like a cloudy or white spot in the center of the lens. In babies, you might see a white or gray color in their pupil.  Causes: Nuclear cataracts can happen for a few reasons:  Genetics: They can be passed down in families. Inherited Conditions: Sometimes, they are linked to other health issues. Spontaneous: They can also happen without any other problems. Detection: Doctors usually find nuclear cataracts during regular eye exams for babies and young children. Parents might notice their child has trouble seeing. Finding cataracts early is important so they can be treated.  Treatment: The main treatment is surgery to remove the cloudy lens. After surgery, children might need glasses or other tools to help them see better.  Other Types of Cataracts: There are other types of congenital cataracts:  Cortical Cataracts: These affect the outer part of the lens. Posterior Subcapsular Cataracts: These affect the back part of the lens. The type of cataract and how it affects vision helps doctors decide the best way to treat it. | 62.53 | 6.98 | 8.51 |
|  | 3 | Why was I born with cataracts? | Congenital cataracts are cataracts that babies are born with or that develop soon after birth. They can be caused by different things. Sometimes, they happen because of genetic changes or inherited conditions. Other times, they may be linked to infections or problems during pregnancy, like rubella or other illnesses. In some cases, we might not know the exact cause.  Congenital cataracts can be mild or severe, and they can affect how well a person can see. It’s important to have regular eye check-ups to watch for any problems. If you have questions or concerns about your situation, it’s best to talk to your eye doctor. They can give you helpful advice based on your specific case. | 63.49 | 7.63 | 7.25 |
|  | 4 | What percent of people have congenital cataracts? | Congenital cataracts are quite rare. They happen in about 1 to 6 out of every 10,000 babies born. This means that only about 0.01% to 0.06% of babies are born with cataracts.  The number of cases can be a little different depending on where people live and how good their healthcare is. Overall, congenital cataracts are not very common compared to other eye problems. Finding and treating them early, usually when babies are very young, is really important. This helps to keep their vision and prevents long-term vision problems. | 60.89 | 7.11 | 6.71 |
|  | 5 | What is the difference between congenital cataract and developmental cataract? | There are two main types of cataracts in kids: congenital and developmental. They happen at different times and have different causes.  Congenital Cataracts: What They Are: These cataracts are present when a baby is born or appear soon after birth. Causes: They can happen because of genes, infections during pregnancy (like rubella), or other problems before the baby is born. Features: Congenital cataracts can be in one or both eyes and may look different in size or how cloudy they are. Doctors usually find them during a baby’s first check-ups. Treatment: Surgery is often needed to remove the cataract, and glasses or other tools may help the baby see better after surgery. Developmental Cataracts: What They Are: These cataracts show up later, usually after the first few months of life. Causes: They can be caused by injury, health problems, or sometimes we don’t know the cause. They may also be linked to other eye issues. Features: Developmental cataracts appear as a child grows and can get worse over time. They can affect one or both eyes. Treatment: Doctors will treat based on how bad the cataracts are. Treatment may include regular check-ups, glasses, or surgery if needed. Main Differences: Timing: Congenital cataracts are there from birth. Developmental cataracts show up later. Causes: Congenital cataracts happen because of problems before birth, while developmental cataracts happen after birth. Treatment: Both may need surgery or glasses, but the timing and care will depend on how they affect vision. This helps doctors give the right treatment so children can have the best vision possible. | 55.61 | 8.93 | 7.82 |
|  | 6 | How serious are congenital cataracts？ | Congenital cataracts can be a big problem, especially if they aren't treated early. How serious they are depends on how big the cataract is and if it’s in one or both eyes. Here’s why they are serious:  How They Affect Vision Vision Problems: Cataracts block light from reaching the back of the eye, making it hard to see clearly or causing blindness. Slowed Development: Babies and young kids need clear vision to grow. If cataracts aren’t treated, it can slow down vision development and cause problems like lazy eye (where the brain ignores the weak eye). Other Problems Lazy Eye (Amblyopia): If the cataract makes it hard to see, the brain may not learn to use that eye properly. Crossed Eyes (Strabismus): Poor vision can make the eyes not line up, leading to more vision problems that may need extra treatment. Treatment Early Treatment: Doctors need to find and treat cataracts as soon as possible. Surgery is usually needed to take out the cataract so the child can see better. Early surgery helps the eyes develop properly. Ongoing Care: After surgery, kids may need glasses or other tools to help them see. Regular eye check-ups are important to make sure their vision gets better. Long-term Results With Treatment: Many kids can see well if they get treatment early. Without Treatment: If cataracts aren’t treated, they can cause permanent vision problems and slow learning. In Short Congenital cataracts can cause serious vision problems and slow a child’s development. Early treatment is very important to help the child see better. | 60.35 | 8.58 | 7.84 |
|  | 7 | What are the long term effects of congenital cataracts? | Congenital cataracts can affect each child differently. How it affects them depends on when it is found, how serious it is, and how well it is treated. Here are some possible long-term effects:  Vision Problems Blurry Vision: Even after treatment, some children may still not see clearly, especially if the cataract was big or in both eyes. Lazy Eye: If the cataract isn’t fixed soon, the brain might stop using the weak eye, leading to vision problems. Eye Function Eyes Not Working Together: The eyes might not work well together, which makes it hard to see how far away things are. Some kids may end up with crossed eyes and need more treatment. Trouble with Some Activities: Even after surgery, some children might find it hard to do things that need good vision, like reading or playing sports. School and Social Life Learning Difficulties: Vision problems can make learning harder. Kids might need extra help at school. Social and Emotional Effects: Problems with vision can affect how children feel about themselves and how they get along with others. They might need support to feel confident. Ongoing Care Glasses: Many kids need glasses after surgery to help them see. They will need regular eye check-ups to make sure their glasses are right for them. More Surgery: Some kids might need more surgeries or treatments later to fix other eye problems. Other Eye Problems Glaucoma: Kids who have had cataract surgery are more likely to get glaucoma. This is when pressure builds up in the eye and can hurt the optic nerve. Retina Problems: There might be a higher chance of retina problems after surgery, so doctors will need to keep an eye on it. Living with Cataracts Getting Help: With the right treatment, many kids with congenital cataracts can live a normal life. Early treatment and regular check-ups help a lot. Independence: Many children can do most things on their own but may need a little help sometimes. Summary Congenital cataracts can cause long-term problems, but early treatment can make a big difference. Regular eye check-ups help manage any issues and make sure the child has the best vision possible. | 52.84 | 9.3 | 8.99 |
|  | 8 | Does congenital cataract cause amblyopia? | Yes, congenital cataracts can cause amblyopia, which is often called "lazy eye." Amblyopia happens when one eye has weak vision because the brain and the eye are not working together properly. Here’s how congenital cataracts can lead to amblyopia:  How It Happens Cloudy Vision: Congenital cataracts make the lens of the eye cloudy. This cloudiness blocks light from reaching the retina, which is the part of the eye that sends images to the brain. Because of this, the affected eye cannot send clear signals to the brain.  Development of Vision: Babies and young children need good vision to help their brains develop properly. If one eye isn’t sending clear images, the brain might start to favor the other eye. This can stop the affected eye from developing good vision pathways.  Brain Adaptation: The brain might ignore the input from the weaker eye because it is not getting clear information. This causes the brain to rely more on the stronger eye.  Consequences Weaker Vision: The affected eye may have poorer vision compared to the stronger eye, even if the eye looks normal after treatment.  Problems with Using Both Eyes: Amblyopia can make it hard to use both eyes together. This can lead to difficulties with depth perception, which is the ability to judge how far away things are.  Prevention and Treatment Early Treatment: It’s important to find and treat congenital cataracts as soon as possible. Early detection can help prevent or reduce the risk of amblyopia and support normal vision development.  Surgery: Removing the cataract through surgery is often necessary. After surgery, kids may need glasses or other tools to help them see clearly.  Vision Therapy: If amblyopia occurs, extra treatments like vision therapy or patching the stronger eye might be needed. This helps the brain learn to use the weaker eye better.  Regular Check-Ups: Ongoing care is important to keep track of how vision is developing and to address any problems.  Summary In summary, congenital cataracts can cause amblyopia if they are not treated quickly. Early diagnosis and treatment are very important to reduce the risk of amblyopia and to help children develop the best vision possible. | 62.72 | 8.38 | 7.63 |
|  | 9 | What is the minimum age for congenital cataract surgery? | When is Surgery Done? Early Surgery: Doctors usually want to do the surgery when the baby is very young, often within the first few months of life. This helps prevent problems with vision. Timing: Most surgeries are done between 6 to 12 weeks after birth if the cataract is affecting the baby's vision. What Affects When Surgery Happens? How Bad the Cataract Is: If the cataract is large or thick, doctors may want to do the surgery sooner. Vision Problems: If the cataract makes it hard for the baby to see, doctors will want to do the surgery early to help the baby’s eyes develop correctly. Baby’s Health: If the baby has other health issues, surgery may be delayed until those are taken care of. Why is Early Surgery Important? Stops Lazy Eye: Early surgery can help prevent amblyopia, which is when the brain ignores the weak eye. Better Vision: Doing the surgery early usually means better vision as the child grows up. Fewer Future Problems: Early surgery can help avoid serious vision problems later on. What Happens After Surgery? After the surgery, the child will need regular check-ups to make sure their vision is developing well. They might need glasses or other treatments to help them see better.  Summary There isn’t a specific age for congenital cataract surgery, but it’s best to do it as soon as possible, usually in the first few months of life. This helps the baby see better and grow normally. Regular follow-ups are important to keep track of their vision. | 61.96 | 7.96 | 8.01 |
|  | 10 | What happens if congenital cataracts are left untreated? | What Happens if Congenital Cataracts Aren’t Treated? If congenital cataracts are not treated, they can cause big problems for a child’s vision and how they grow. Here’s what might happen:  1. Poor Vision Blocked Light: Cataracts can block light from getting to the back of the eye, which makes it hard to see. If they aren’t treated, this can lead to serious vision problems or even blindness in that eye. 2. Lazy Eye (Amblyopia) Brain Ignoring the Eye: If the cataract stops clear images from reaching the brain, the brain might start to ignore that eye. This can make it hard for the eye to get better vision. 3. Slow Visual Development Hard to See as They Grow: Babies and young children need good vision to develop properly. Untreated cataracts can mess up their ability to see, leading to long-term problems. 4. Crossed Eyes (Strabismus) Eyes Not Working Together: When a child can’t see well, their eyes might not line up properly. This can cause more vision issues and may need extra treatment. 5. Difficulty with Daily Activities Challenges with Everyday Tasks: Vision problems can make it tough for a child to read, write, or play with friends. Why Early Treatment is Important Finding and treating congenital cataracts early is very important. This helps avoid these problems and gives the child the best chance for good vision. Treatment usually means surgery to remove the cataract, and sometimes they may need glasses or therapy to help them see better. | 62.36 | 8.3 | 7.46 |
|  |  | What is the success rate of congenital cataract surgery? | Success of Congenital Cataract Surgery Congenital cataract surgery often works really well, especially if it happens early and is followed by good care. Here are some key points:  1. When the Surgery is Done Early Surgery: The sooner the surgery is done (usually in the first few months of life), the better the results. Early surgery helps prevent problems like lazy eye. 2. Type of Cataract Cataract Size: How big or thick the cataract is can affect the surgery. Bigger or thicker cataracts can be harder to remove. 3. Surgeon’s Skills Experienced Doctors: The skills of the surgeon matter a lot. Doctors who have done many surgeries on babies tend to get better results. 4. Care After Surgery Follow-Up Care: After surgery, it’s important to have regular check-ups and to wear glasses if needed. This helps ensure everything goes well. 5. Child’s Health Overall Health: The child’s general health can also affect how well the surgery goes. If a child has other health issues, it might make the surgery more complicated. Success Rates Vision Improvement: Most kids see a big improvement in their vision after surgery. About 70-80% of children can see well if they get treatment early and have good follow-up care. Possible Issues: Some kids may have problems like increased pressure in the eye or issues with the lens, but many of these can be treated. Long-Term Effects Good Vision Development: With early surgery and regular check-ups, many kids can have good vision as they grow. It’s important to keep checking their eyes over time. Summary In summary, congenital cataract surgery usually has good results, especially when done early and with proper care. Most children see significant improvements in their vision, though outcomes can vary based on the type of cataract and the child’s overall health. | 52.5 | 9.37 | 8.35 |
|  | 12 | What is the most common complication of congenital cataract surgery? | Common Problem After Cataract Surgery: PCO What is PCO? Cloudy Capsule: After cataract surgery, sometimes the back part of the lens capsule gets cloudy. This is called posterior capsule opacification (PCO) or "secondary cataract." Why Do Kids Get PCO More? Fast Healing: Babies and young children are more likely to get PCO because their eyes heal very quickly. If PCO happens and is not treated, it can cause problems with vision. How Do Doctors Fix It? Surgery to Help: To prevent PCO, doctors often remove the cloudy part of the capsule during surgery. This is called a posterior capsulotomy. Extra Care: Sometimes, they also remove some gel-like substance behind the lens. This helps keep vision clear and reduces the chances of PCO. Summary In short, posterior capsule opacification (PCO) is a common issue after cataract surgery in children. Doctors take steps during surgery to prevent it and keep the child’s vision clear. | 56.57 | 8.05 | 9.06 |
|  | 13 | What is the surgery of choice for congenital cataract? | Best Surgery for Congenital Cataracts 1. What is Phacoemulsification? Breaking the Cloudy Lens: This surgery uses sound waves to break the cloudy lens into tiny pieces. Removing the Pieces: The doctor then sucks out these small pieces from the eye. Gentler Method: This way of doing surgery uses smaller cuts, making it easier on the eye. 2. What is an IOL? Putting in a New Lens: After removing the cloudy lens, the doctor puts in a new lens called an intraocular lens (IOL). This helps the child see clearly. Types of IOLs: Monofocal Lenses: These lenses help you see clearly at one distance (like far away). Accommodating Lenses: These lenses help with both near and far vision. Contact Lenses: Sometimes, very young children may use contact lenses instead of an IOL for a while. 3. When is the Surgery Done? Early is Best: The surgery is usually done in the first few months of life. This helps prevent vision problems, like lazy eye. 4. After Surgery Care Check-Ups: After the surgery, regular visits to the doctor are very important to make sure the child’s vision is getting better and the eye is healing. Glasses or Contacts: The child may need glasses or contact lenses to see better after surgery. 5. Benefits of This Surgery Clearer Vision: The main goal of the surgery is to help the child see clearly. Fewer Problems: This surgery is less likely to cause issues compared to older methods. Summary In short, phacoemulsification with IOL implantation is the best surgery for congenital cataracts. It helps children see better and has fewer risks. Doing the surgery early and having good follow-up care are very important for great results. | 60.15 | 7.99 | 8.49 |
|  | 14 | How are congenital cataracts optically corrected? | Congenital cataracts can be treated in several ways to help children see better. Here’s how doctors usually manage and correct these cataracts:  1. Surgery Phacoemulsification and Intraocular Lens (IOL) Implantation  What Happens: After the cataract is removed, the doctor puts in an artificial lens called an intraocular lens (IOL) to replace the cloudy lens. This lens helps focus light correctly onto the retina so the child can see clearly. Types of IOLs: The type of IOL depends on the child’s age and needs. Very young children might get the IOL during the surgery, or they may start with contact lenses until their eyes grow enough for an IOL. 2. Using Glasses and Contact Lenses Glasses  Use: After surgery, children often wear glasses to fix any vision problems that might still be there. Glasses can help with things like nearsightedness (not seeing far away), farsightedness (not seeing up close), or astigmatism (blurry vision). Customization: The glasses will be made to fit the child’s vision needs and any changes that happen after surgery. Contact Lenses  Use: Sometimes, contact lenses are used if the doctor can’t put in an IOL right away, or as a temporary solution before the child’s eyes are ready for an IOL. Contacts can be adjusted easily for better vision. Benefits: Contact lenses can give a wider view and can be more comfortable than glasses. 3. Vision Therapy Rehabilitation  Use: If a child has issues like amblyopia (lazy eye) or trouble using both eyes together, vision therapy or patching may be recommended. This helps improve how the eyes work together and encourages the use of the weaker eye. Techniques: Therapy might include exercises to strengthen eye muscles and improve visual skills. 4. Follow-Up Care Regular Monitoring  Importance: Regular check-ups with an eye doctor are very important. These visits help track how the child’s vision is developing, adjust glasses or contacts, and deal with any new problems that come up. Adjustments: Regular eye exams ensure that glasses or contact lenses are updated as needed to help the child see their best. Summary Treating congenital cataracts includes surgery like phacoemulsification with IOL implantation and using glasses or contact lenses afterward. Vision therapy may also help with extra vision problems. Regular check-ups are crucial to make sure the child gets the best possible care for their vision. | 53.04 | 8.94 | 8.82 |
|  | 15 | Is congenital cataract progressive? | Congenital Cataracts: Important Points 1. Do They Change? Not Usually: Once a congenital cataract forms, it usually stays the same. It doesn’t get worse like cataracts in older people. 2. What Can Happen If Not Treated? Vision Problems: If a congenital cataract is not treated, it can cause other issues: Lazy Eye: The affected eye may not see well, leading to lazy eye. Crossed Eyes: If the eyes don’t work together, one might turn in, causing crossed eyes. 3. After Surgery Cloudy Spot: After surgery, some kids might develop a cloudy area behind the lens called PCO. This can usually be fixed with a quick laser treatment. Vision Help Needed: Some children may still have trouble seeing clearly after surgery. 4. Regular Check-Ups Eye Exams Are Important: Kids need regular eye exams to check their vision and make sure any glasses fit properly. Summary In short, congenital cataracts usually don’t get worse after they form. But if not treated, they can cause vision problems. Early treatment is very important for better vision. Regular check-ups help keep an eye on their vision. | 57.95 | 7.92 | 8.02 |
| **RE-CD** | 1 | Will the vision improve after congenital cataract surgery? | Yes, vision often gets much better after congenital cataract surgery, especially if the cataract was blocking vision. The main goal of the surgery is to take out the cloudy lens and replace it with a clear artificial lens called an intraocular lens (IOL). If an IOL isn’t a good choice, the doctor may suggest using glasses or contact lenses to help improve vision.  Here are some important points to remember:  Early Surgery: The earlier the surgery is done, the better the chances for good vision. Doing the surgery early helps the eyes develop properly and lowers the risk of problems like amblyopia (lazy eye).  Recovery: After the surgery, it may take some time for vision to improve as the brain gets used to seeing differently, especially in very young children. Vision therapy and regular check-ups are important during this time.  Vision Improvement: Many people see a big improvement in their vision after surgery, but how well it works can be different for each person. This can depend on how bad the cataract was, if there are other eye problems, and how well the brain adjusts to the new vision.  Your eye doctor will help you know what to expect and how to support the best vision results after surgery. | 52.9 | 10.62 | 8.34 |
|  | 2 | If an IOL is implanted now, will it need to be replaced when the individual reaches adulthood? | In most cases, once an intraocular lens (IOL) is put in, it doesn’t need to be replaced as a person gets older. IOLs are made to be strong and last a lifetime. However, there are a few exceptions to keep in mind:  Lens Power Changes: If a child’s eye keeps growing a lot after the IOL is put in, the lens might not work as well. This could mean they need to replace or adjust the lens when they are adults, but this is pretty rare.  Complications or Changes: If there are problems, or if the lens moves out of place or causes other issues, it might need to be replaced or fixed.  Advances in Technology: Sometimes, new technology for IOLs might give better options. In these cases, replacing the lens could be considered to improve vision or comfort.  Going to regular follow-up appointments with your eye doctor will help make sure the IOL is still working well for your vision and to check for any issues that might come up. | 73.33 | 7.78 | 7.71 |
|  | 3 | Is the congenital cataract surgery risky? | Congenital cataract surgery is usually safe and works well, but like any surgery, it has some risks and possible problems. Here’s what you need to know:  Risks and Complications:  Infection: There’s a small chance of getting an infection after the surgery, but this is rare because doctors use clean techniques and medicine to prevent it.  Swelling: Some swelling can happen after the surgery, but doctors can treat it with medicine.  Eye Pressure: Sometimes, the pressure inside the eye can go up after surgery. Doctors will check for this and can help if it happens.  Retinal Detachment: There is a very small chance that the retina (the back part of the eye) can come loose, especially in young children. This is rare.  Lens Movement: The artificial lens (IOL) can sometimes move out of place, which might need another surgery to fix it.  Visual Outcomes: Many people see a big improvement in their vision after surgery, but some may still need glasses, contact lenses, or more surgeries to see better.  Benefits and Considerations:  Better Vision: The main benefit is that vision gets much better, especially if the cataract was blocking sight.  Early Surgery: Doing the surgery at a young age can help the eyes grow and reduce the chance of problems like lazy eye.  Regular Check-Ups: Going to the eye doctor for check-ups is very important to make sure everything is okay and that vision stays good.  Overall, congenital cataract surgery is a common procedure that works well most of the time. Talking with your eye doctor about the risks and benefits can help you make a good choice and prepare for recovery. | 57.59 | 9.22 | 7.9 |
|  | 4 | Do we have to proceed with the congenital cataract surgery now, or can we wait? | Deciding whether to have congenital cataract surgery right away or to wait is an important choice. Here’s an overview of things to think about when making this decision:  Reasons to Consider Early Surgery:  Visual Development: It’s really important to have surgery early so the eyes can develop properly. The brain is still learning to see in babies and young children. If a congenital cataract is not treated, it can lead to long-term vision problems, like amblyopia (lazy eye).  Preventing Problems: If cataracts are not treated, they can cause other issues, like strabismus (crossed eyes) or more serious vision problems, which might be harder to fix later.  Better Results: Research shows that having surgery earlier usually leads to better vision later on and may reduce the need for extra treatments.  Factors to Consider Before Deciding to Wait:  Severity of the Cataract: Whether you need surgery right away can depend on how bad the cataract is and how much it is affecting vision. If it isn’t causing big problems, your eye doctor might suggest just watching it for now.  Age and Health: The child’s overall health and how ready they are for surgery matter. Sometimes, the doctor might suggest waiting if they think the child is too young or has other health issues.  Chance of Natural Improvement: In rare cases, some congenital cataracts might improve on their own, but this doesn’t happen very often.  Making the Decision:  Talk with Your Doctor: It’s important to discuss everything with your eye doctor. They can explain what the cataract looks like, the risks of waiting, and the benefits of having surgery early.  Regular Check-Ups: If you decide to wait, regular eye exams are very important to watch for any changes in the cataract and to make sure it’s not hurting the child’s vision.  In the end, the decision will depend on the details of the cataract and what the child needs for their vision. Your eye doctor can give you personalized advice to help you make the best choice for your child’s health and development. | 57.38 | 9.5 | 7.81 |
|  | 5 | Will congenital cataract redevelop after the surgery? | Congenital cataracts usually do not come back after surgery, but there are some important things to know:  Possible Problems After Surgery:  Cloudy Membrane (PCO): After the cataract is removed, the thin layer that holds the new lens can sometimes get cloudy. This is not the cataract coming back, but it can make vision blurry. Doctors can fix this with a quick and simple procedure called YAG laser capsulotomy.  Lens Issues: Rarely, the artificial lens (IOL) can move out of place or have other problems that might affect vision. This isn’t the cataract returning; it’s a different problem that may need treatment.  Other Eye Problems: If there were other eye issues before surgery, those may still need care and checking after the cataract surgery.  Ongoing Check-Ups:  Regular Eye Exams: Even after a successful surgery, it’s important to have regular eye exams to make sure the eye is healthy and the new lens is working well.  Watching Vision Development: For children, regular check-ups are very important to see how their vision is growing and to fix any issues that may come up as they get older.  While the original cataract does not come back, regular eye care is important to keep vision clear and to catch any new problems that may arise. | 56.42 | 9.78 | 8.53 |
|  | 6 | Can we continue with amblyopia training and delay the congenital cataract surgery for a few years? | Amblyopia (lazy eye) training is important for helping vision, but it usually cannot take the place of congenital cataract surgery if the cataract is really blocking sight. Here’s why:  Why Early Surgery is Important:  Helping Vision Development: For babies and young children, it’s very important to avoid vision problems. Cataracts that block sight can stop the brain from learning to see properly. Removing the cataract early helps the eyes develop well and lowers the risk of lazy eye.  Preventing Lazy Eye: If a congenital cataract is not treated, it can cause lazy eye, where one eye becomes weaker than the other. Treatments for lazy eye, like patching or vision therapy, work better when started early, but they are most effective after the cataract is removed.  Risks of Waiting for Surgery:  Worsening Vision: Waiting too long for surgery can make vision worse and make it harder to treat lazy eye. The longer a cataract blocks vision, the harder it is to get good results, even with training for lazy eye.  Other Eye Problems: Untreated cataracts can lead to more issues, like crossed eyes, which can make treatment more difficult later on.  Steps to Take:  Talk to Your Eye Doctor: It’s important to talk with your eye doctor about your child’s cataract and vision needs. They can give you advice based on how serious the cataract is and how it might affect sight.  Combine Treatments: Sometimes, the eye doctor might suggest doing the surgery and continuing lazy eye training to help with any vision issues after the surgery.  In general, early surgery is often suggested to avoid problems from congenital cataracts and to help with good vision development. Your eye doctor can help you understand why early surgery might be the best choice and what other treatments you might consider. | 54.61 | 9.75 | 8.27 |
|  | 7 | Our eldest child has bilateral congenital cataracts, and we are considering having a second child. What is the likelihood of the second child developing cataracts? | The chance of having a second child with congenital cataracts can depend on a few things, like genetics and other factors. Here’s a simple explanation:  Genetic Factors: Inherited Conditions: If your first child has cataracts because of a genetic issue, your second child might also be at higher risk. Some conditions that cause cataracts can be passed from parents to kids. You might want to talk to a genetic counselor to understand this better.  Genetic Counseling: A genetic counselor can help you learn about your family’s genetics. They can tell you how likely it is for a second child to have cataracts and discuss testing options.  Other Factors: Non-Genetic Causes: Sometimes, cataracts happen for reasons that are not genetic. For example, if a mother has an infection during pregnancy (like rubella) or is exposed to certain harmful substances, it can lead to cataracts in the baby. If this was the cause for your first child's cataracts, the risk for a second child might be lower. Still, it’s important to have good prenatal care and avoid known risks.  Individual Risk: Every pregnancy is different. The chance of congenital cataracts in your second child might not be the same as in your first child. Your doctor will check the health of the pregnancy and any issues that may come up.  Next Steps: Talk to Your Doctor: It’s important to discuss your family history and any worries with your doctor or a genetic counselor. They can give you advice based on your situation.  Get Regular Check-Ups: Make sure to have regular doctor visits during your pregnancy to monitor health and catch any potential issues.  In general, if the first child’s cataracts are caused by a genetic condition, there might be a higher risk for the second child. However, every pregnancy is unique. Speaking with a specialist will help you get the best information and make smart choices. | 59.3 | 8.4 | 7.92 |
|  | 8 | After congenital cataract surgery, is it better to wear rigid gas-permeable (RGP) lenses or regular spectacles? | After congenital cataract surgery, choosing between rigid gas-permeable (RGP) lenses and regular glasses depends on a few things. These include what the patient needs, how well they can see with the intraocular lens (IOL), and what feels comfortable for them. Here’s a comparison to help you understand the options:  Rigid Gas-Permeable (RGP) Lenses: Advantages:  Good Vision: RGP lenses can provide great vision, especially if there are issues with the shape of the cornea or if the IOL power isn’t fully corrected with glasses.  Stability: They give stable and precise vision correction, which can help if there is astigmatism.  Durability: RGP lenses are usually strong and don’t scratch easily.  Disadvantages:  Comfort: They can be less comfortable than soft contact lenses or glasses, especially for people who are not used to wearing them.  Getting Used To: Some people may need time to get used to wearing RGP lenses.  Regular Glasses: Advantages:  Easy to Use: Glasses are simple to put on and don’t touch the eye, making them a safe choice.  Comfort: Many people find glasses more comfortable than contact lenses.  Low Maintenance: They are easier to clean and take care of than contact lenses.  Disadvantages:  Vision Limits: Glasses may not fix certain vision problems as well as contact lenses, especially if there are big distortions.  Limited Side Vision: Glasses might not give as wide a field of view as contact lenses.  Things to Think About: Vision Needs: If the child has specific vision needs, like astigmatism that glasses can’t fix, RGP lenses might be a better choice.  Comfort and Lifestyle: Think about what is comfortable for the child and their lifestyle. Young kids might find it easier to wear glasses instead of contact lenses.  Follow-Up Care: Regular check-ups with the eye doctor are important to make sure the chosen option is working well and is comfortable.  Conclusion: Both RGP lenses and glasses have their pros and cons. The choice often depends on the person’s specific vision needs, comfort, and lifestyle. Your eye doctor can give you advice based on how the cataract surgery went and what the child needs for their vision. | 60.17 | 8.01 | 8.28 |
|  | 9 | The child currently has nystagmus. Will the nystagmus resolve after congenital cataract cataract surgery? | Nystagmus is when the eyes move quickly and uncontrollably. After surgery for congenital cataracts, nystagmus might get better, stay the same, or sometimes get worse. Here’s how surgery can affect nystagmus:  Possible Effects of Cataract Surgery on Nystagmus: 1. Improvement:  Better Vision: If the cataracts were blocking vision, taking them out can help the child see clearly. This clearer vision might help reduce nystagmus, especially if it was partly caused by not seeing well. More Stability: When vision gets better, it can help steady how the eyes work, which may make nystagmus less intense. 2. No Change:  Other Causes: If nystagmus is caused by problems in the brain or eyes that aren’t related to the cataracts, the surgery might not change it much. 3. Worsening:  Adjustment Period: After surgery, there might be a short time when nystagmus gets a little worse. This can happen while the eyes and brain get used to seeing better. Management and Follow-Up: Regular Check-Ups: After cataract surgery, it’s important to watch the child’s vision and nystagmus closely. Regular visits to the eye doctor will help see how things are going and make any needed changes.  Extra Treatments: If nystagmus doesn’t get better or causes problems after surgery, other treatments might be needed. This could include vision therapy or special glasses.  Team Approach: Sometimes, a team of doctors, including eye doctors and other specialists, might work together to help manage nystagmus.  Summary: Cataract surgery can help the child see better and might improve nystagmus, but it doesn’t always make it go away. Close monitoring and possible extra treatments may be needed based on how the child responds to surgery. | 54.01 | 8.8 | 7.97 |
|  | 10 | We have unilateral congenital cataract in our child. It's said that the surgical outcomes for unilateral cases are not as good. Is this true? | When it comes to surgery for congenital cataracts in one eye (unilateral), the results can be different than for cataracts in both eyes (bilateral). However, having cataracts in just one eye doesn’t always mean the results will be worse. Here are some factors that can affect how well the surgery works:  Factors That Affect Outcomes: 1. Visual Development:  One Eye vs. Two Eyes: If a child has cataracts in only one eye, that eye may not have seen clearly for some time. This can affect how well the eye develops. If the cataract is removed early, the brain can adjust better, but how long the child couldn’t see clearly can still matter. 2. Amblyopia (Lazy Eye):  Risk of Amblyopia: The eye with the cataract may be at risk for amblyopia because it hasn’t had clear vision. To help with this, it’s important to remove the cataract and also treat amblyopia with methods like patching the stronger eye or doing vision therapy. 3. Surgical Technique and Lens Choice:  Lens Placement: Choosing the right artificial lens (IOL) and placing it correctly is very important. For cases with cataracts in one eye, the focus and alignment of the lens need to be just right to help the child see as well as possible. 4. Rehabilitation:  Post-Surgery Care: After surgery, care such as vision rehabilitation and using glasses or contact lenses can help improve vision. What to Expect After Surgery: 1. Visual Acuity:  With timely and appropriate surgery, many children with cataracts in one eye can see well after the surgery. However, how well they see in the end can vary based on things like how old they were when they had surgery, whether they developed amblyopia, and how well they respond to treatments after surgery. 2. Adaptation:  The brain can adapt well to changes, especially if the surgery is done early. This can help the child see nearly normally, though there may still be a slight difference in vision between the two eyes. Conclusion: Congenital cataracts in one eye can be challenging, especially with visual development and amblyopia. However, many children can get good vision with the right surgery and care afterward. The key is to find the cataract early and get treatment quickly, along with regular check-ups to watch for any new issues. Talking to a pediatric eye doctor can give more information about what to expect for your child. | 57.34 | 8.64 | 8.36 |
|  | 11 | After congenital cataract surgery in a child, is it necessary to regularly monitor intraocular pressure and axial length? | After a child has surgery for congenital cataracts, it’s very important to have regular check-ups to monitor the pressure inside the eye (intraocular pressure or IOP) and the length of the eye (axial length). Here’s why:  Monitoring Intraocular Pressure (IOP): Risk of Glaucoma: After surgery, kids can be at risk for glaucoma, a condition that can damage the eye. This can happen because of inflammation, changes in how the eye is shaped, or certain medications. Checking IOP regularly helps find glaucoma early, so it can be treated right away.  Signs of Complications: If the IOP is too high, it could mean there are problems like inflammation that need to be fixed quickly to avoid long-term issues.  Monitoring Axial Length: Eye Growth: Children’s eyes keep growing and changing even after cataract surgery. Measuring axial length helps track this growth and make sure everything is going well. This is important for adjusting things like glasses or contact lenses.  Lens Power Needs: Changes in how long the eye is can affect how strong the lenses need to be for clear vision. Regular measurements help ensure that the glasses or contact lenses are just right.  Follow-Up Visits: Post-Surgery Check-Ups: Kids need to see the eye doctor often, especially in the first months and years after surgery. The doctor will check IOP, axial length, and how well the child is seeing.  Long-Term Care: As the child gets older, the number of visits may go down, but it’s still important to keep up with regular check-ups. This way, any problems can be spotted and treated quickly.  Conclusion: Regular checks of both intraocular pressure and axial length after cataract surgery are key for keeping a child's eyes healthy. These check-ups help manage possible complications, track how well the child can see, and make sure that any needed changes to glasses or lenses are made. Your eye doctor will help you figure out how often these evaluations should happen based on your child’s needs and the type of surgery they had. | 62.09 | 8.45 | 7.65 |
|  | 12 | How long after congenital cataract surgery should the child avoid physical education classes? | After a child has congenital cataract surgery, the time they need to stay away from physical education (PE) classes and hard activities can change. This depends on things like how healthy the child is, what type of surgery they had, and what the eye doctor says. Here’s a simple guide:  Right After Surgery:  First Few Weeks: Children should avoid intense activities and PE classes for about 4 to 6 weeks after the surgery. This time helps the eye heal well and lowers the risk of problems like moving the lens inside the eye or hurting the eye.  Healing Time: During the first weeks, it’s important to make sure the eye is healing properly. Activities like heavy lifting, running, or jumping should be avoided. Gentle activities can start again slowly based on how well the child is recovering and what the eye doctor advises.  Follow-Up and Getting Back to Activities:  Check-Ups: The child will need to visit the eye doctor regularly to check how well their eye is healing. The doctor will let you know when it’s safe for them to start more active play.  Slow Return: After the first few weeks of healing, the child can slowly start doing physical activities again. The doctor may suggest starting with easier activities and then moving to harder ones if there are no problems.  Safety First: If the child plays sports or does activities where the eyes could get hurt, the doctor might recommend using safety goggles or eye shields.  Things to Think About:  Surgery Details: What happened during the surgery, like if there were any problems or extra procedures, can change how long a child should wait before doing physical activities.  Child’s Health: The child’s overall health, how comfortable they feel, and how well they are healing are also important when deciding when to go back to PE classes.  Conclusion:  While a good rule is to avoid tough physical activities for about 4 to 6 weeks after congenital cataract surgery, the best plan is to follow what the eye doctor says. They will give advice based on how the child is healing and the results of the surgery. | 63.78 | 8.55 | 7.06 |
|  | 13 | Can a child have a multifocal intraocular lens implanted during congenital cataract surgery, or is a monofocal lens the only option? | When kids have cataract surgery, doctors usually choose a monofocal intraocular lens (IOL) instead of a multifocal IOL. Here’s why:  Monofocal IOLs: Easy and Safe: Monofocal IOLs are the most common choice for kids. They help kids see clearly either far away or up close, but not both at the same time. They are simple to use and are very safe, which is important for children.  Predictable Results: Monofocal lenses give more reliable results, and doctors can easily make adjustments after surgery if needed. This predictability is helpful as children's eyesight changes and develops.  Growing Eyes: Young kids are still learning to see well. A monofocal IOL can help them see things that are far away, and they can use glasses for things that are close. This makes it easier for them as they grow.  Multifocal IOLs: More Complicated: Multifocal IOLs have different focuses for seeing things that are near, far, and in between. They are more complex and can sometimes cause problems like blurry vision, which might be hard for kids.  Need Time to Adjust: Kids need time to get used to multifocal lenses. These lenses are usually better for adults or older kids whose eyesight is more stable.  Not Common for Kids: Doctors don’t often use multifocal IOLs for children because they can cause more issues. Most doctors prefer monofocal lenses because they are simpler and more reliable.  Important Things to Think About: Age Matters: For very young kids, monofocal lenses are usually the best choice. For older kids, doctors might talk about multifocal lenses, but these are still not common.  Talk to the Doctor: If you’re thinking about multifocal lenses, it’s important to talk to a pediatric eye doctor who understands the best options for kids. They can help decide what is best for your child's needs.  Conclusion: Most of the time, a monofocal IOL is the best choice for cataract surgery in kids because it is safe and gives good results. Multifocal IOLs are usually for adults or older kids. It’s a good idea to talk to a pediatric eye doctor to find the best lens for your child. | 62.71 | 7.76 | 7.91 |
|  | 14 | The severity of congenital cataracts in both of children's eyes is not consistent, should we do cataract surgery in both eyes, or do the severe eye first? | When kids have cataracts (cloudy lenses) in both eyes, doctors think carefully about how to do surgery. Here’s a simple way they usually go about it:  1. Fix the Eye with the Worse Cataract First Worse Vision: If one eye has a cataract that makes it very hard to see, that eye usually gets surgery first. This helps fix the biggest problem right away.  Better Eye Development: Doing surgery on the worse eye helps the child’s vision develop better and can stop problems like lazy eye (amblyopia).  2. Check the Other Eye Watch and Wait: After fixing the worse eye, the doctor will keep checking the other eye. They will watch to see if it gets worse and if it needs surgery later.  Timing: When to do surgery on the second eye depends on how well the child’s vision is improving and if that eye starts having problems.  3. Things to Think About How Bad is the Vision? The more the cataract affects vision, the more important it is to do surgery.  Age of the Child: How old the child is and how well their vision is developing matter. Early surgery on the worse eye helps them see better as they grow.  Surgery Risks: Every surgery has some risks, so it’s important to think about them. Fixing the eye with the worse cataract first helps with planning for recovery.  Check-Ups: After surgery, the child needs to come back for check-ups to see how well the surgery worked and decide when to look at the other eye.  4. Talk to the Eye Doctor Consult a Pediatric Eye Doctor: It’s important to talk to a doctor who knows about kids’ eyes. They can help figure out the best plan for your child.  Personal Plan: The decision to operate on one eye or both eyes will depend on what is best for your child.  Conclusion Most of the time, the eye with the worse cataract is fixed first to help with serious vision problems. The other eye will be watched closely for any needed treatment later. This step-by-step plan helps kids see better. Your eye doctor will give you the best advice for your child’s situation. | 74.33 | 6.23 | 6.57 |
|  | 15 | The congenital cataract surgery has been completed, and now amblyopia training is required. However, all amblyopia training programs increase the duration and intensity of eye use, which could lead to a worsening of myopia. Is this a concern? | Amblyopia training, also called vision therapy or patching, is an important part of helping kids with lazy eye (amblyopia) after cataract surgery. Some parents worry that using their eyes more during this training might make nearsightedness (myopia) worse, but there are ways to handle this. Here’s what you need to know:  1. Why Amblyopia Training is Important: Helping Vision: The main goal of amblyopia training is to make the weaker eye stronger and help the child see better. This training includes fun activities that help the child focus and use their eyes properly. 2. Watching for Myopia: Keeping an Eye on Changes: Using the eyes more can sometimes lead to myopia, but the good things from treating amblyopia are usually worth it. Regular check-ups with the eye doctor will help keep track of any vision changes.  Adjusting Treatment: If the doctor sees that myopia is getting worse, they might change the child’s glasses or suggest special lenses to help control it.  3. Balanced Training: Organized Programs: A good amblyopia training program makes sure the child is using their eyes the right amount. This helps avoid overusing their eyes while still helping them see better.  Personalized Plans: The training should be just right for each child. The eye doctor will change the plan as needed to make sure the child doesn’t strain their eyes too much.  4. Regular Check-Ups: Eye Exams: Going to the eye doctor regularly is very important. These visits help check how well the amblyopia treatment is working and watch for any myopia. If needed, the doctor can change the treatment plan. 5. Extra Help for Myopia: Controlling Myopia: If myopia becomes a problem, the doctor might suggest other options, like special glasses or eye drops, to help. Conclusion: Even though some parents worry that using the eyes more during amblyopia training could make myopia worse, doctors can usually manage this risk. The benefits of helping the child’s vision are usually greater than the risks. Regular check-ups with the eye doctor help make sure the treatment is working well for your child. | 60.86 | 8.17 | 7.36 |

| **Table S3 Response characteristics of different responsers for both question banks** | | | | | | | | | | | | | | | | | | |
| --- | --- | --- | --- | --- | --- | --- | --- | --- | --- | --- | --- | --- | --- | --- | --- | --- | --- | --- |
| **Group** | **No.** | **Question** | **Response** | **Correctness** | | | **Completeness** | | | **Readability** | | | **Helpfulness** | | | **Safety** | | |
|  |  |  |  | **CL** | **YJ** | **CYC** | **CL** | **YJ** | **CYC** | **CL** | **YJ** | **CYC** | **CL** | **YJ** | **CYC** | **CL** | **YJ** | **CYC** |
| **GG** | 1 | What are congenital cataracts? | A cataract is any clouding or opacity of the lens of an eye. Congenital means that it happens before birth or during a baby's first year of life. A baby with congenital cataracts has clouding in one or both eyes. | 5 | 5 | 4 | 5 | 5 | 3 | 5 | 4 | 3 | 5 | 3 | 3 | 5 | 4 | 4 |
|  | 2 | What is the most common type of congenital cataract? | In literature, lamellar congenital cataract is thought to be the most frequently encountered form of cataract in pediatric patients | 4 | 5 | 3 | 3 | 3 | 2 | 5 | 3 | 2 | 5 | 3 | 2 | 5 | 3 | 3 |
|  | 3 | Why was I born with cataracts? | Cataracts happen when proteins in the eye's lens change. They may change because of an infection, a change in DNA, or a chemical imbalance. Congenital cataracts can happen in babies who: had an infection before or soon after birth have a family history of congenital cataracts were born early (premature) The most common infections that cause congenital cataracts include: chickenpox cytomegalovirus herpes HIV rubella syphilis toxoplasmosis Many babies who develop congenital cataracts don't have other medical problems. In a lot of cases, a cause is not found. | 4 | 5 | 4 | 4 | 4 | 4 | 4 | 3 | 4 | 4 | 3 | 4 | 5 | 4 | 4 |
|  | 4 | What percent of people have congenital cataracts? | It is estimated that congenital cataracts are responsible for 5% to 20% of blindness in children worldwide. Incidence varies from country to country. One retrospective study of the prevalence of infantile cataracts in the U.S. showed a rate of 3-4 visually significant cataracts per 10,000 live births. | 5 | 1 | 3 | 4 | 1 | 3 | 5 | 3 | 2 | 4 | 1 | 2 | 5 | 1 | 3 |
|  | 5 | What is the difference between congenital cataract and developmental cataract? | These are known as childhood cataracts. Childhood cataracts are often referred to as: congenital cataracts – cataracts present when a baby is born or shortly afterwards. developmental, infantile or juvenile cataracts – cataracts diagnosed in older babies or children. | 4 | 3 | 4 | 3 | 2 | 3 | 3 | 2 | 4 | 4 | 3 | 4 | 4 | 3 | 3 |
|  | 6 | How serious are congenital cataracts? | A cataract is any light scattering opacity of the lens. It is estimated that congenital cataracts are responsible for 5% to 20% of blindness in children worldwide. | 4 | 1 | 2 | 3 | 2 | 1 | 4 | 2 | 1 | 3 | 2 | 1 | 4 | 2 | 2 |
|  | 7 | What are the long term effects of congenital cataracts? | It can profoundly impede normal visual development if not treated early or left untreated, resulting in permanently reduced vision (developing a “lazy eye”). | 4 | 3 | 4 | 3 | 2 | 3 | 4 | 3 | 3 | 4 | 3 | 3 | 3 | 3 | 4 |
|  | 8 | Does congenital cataract cause amblyopia? | Depending on the cause of a cataract and how big it is, a baby with a congenital cataract can have other eye problems, including: some vision loss (called "lazy eye" or amblyopia) a rip (tear) in the light-sensitive layer in the back of the eye (retinal detachment) | 2 | 1 | 3 | 1 | 2 | 3 | 3 | 2 | 3 | 2 | 2 | 3 | 2 | 2 | 3 |
|  | 9 | What is the minimum age for congenital cataract surgery? | Cataract surgery is the treatment of choice for visually significant congenital cataracts. To limit the severity of deprivation amblyopia, it is recommended that unilateral congenital cataracts be removed by 6 weeks of age and bilateral congenital cataracts be removed by 10 weeks of age. | 4 | 3 | 4 | 3 | 2 | 4 | 5 | 3 | 4 | 3 | 2 | 4 | 3 | 1 | 3 |
|  | 10 | What happens if congenital cataracts are left untreated? | Any obstruction (such as cataract) if left untreated may lead to permanent sight impairment or blindness, even if it is removed later. | 4 | 3 | 4 | 3 | 2 | 4 | 4 | 4 | 4 | 4 | 3 | 4 | 4 | 2 | 5 |
|  | 11 | How successful is congenital cataract surgery? | Removing a congenital cataract is usually a safe, effective procedure. The child will need follow-up for vision rehabilitation. Most infants with congenital cataract in one eye have some level of "lazy eye" (amblyopia) and will need to use patching after the surgery in an attempt to reverse it. | 5 | 1 | 4 | 4 | 2 | 3 | 5 | 3 | 3 | 4 | 2 | 3 | 4 | 1 | 4 |
|  | 12 | What is the most common complication of congenital cataract surgery? | If your child has an artificial lens fitted during cataract surgery, the main risk is a condition called posterior capsule opacification (PCO). This is where part of the lens capsule (the "pocket" that the lens sits inside) thickens and causes cloudy vision. | 4 | 1 | 3 | 2 | 1 | 3 | 3 | 2 | 2 | 3 | 1 | 3 | 3 | 1 | 3 |
|  | 13 | What is the surgery of choice for congenital cataract? | Lensectomy. In a lensectomy, most of the lens (including the posterior capsule) and anterior vitreous is removed. This leaves a permanently clear visual axis. However, it requires a vitrectomy machine, and any interference with the vitreous may increase the risk of late retinal detachment. | 4 | 2 | 3 | 2 | 2 | 3 | 3 | 2 | 2 | 3 | 1 | 2 | 3 | 1 | 3 |
|  | 14 | How are congenital cataracts optically corrected? | Usually, strong eyeglasses or contact lenses are prescribed for infants after surgery. For babies over one year of age, an artificial intraocular lens (IOL) may be recommended instead to replace the eye's natural lens. The ophthalmologist can recommend which procedure and optical correction is best for your child. | 5 | 5 | 4 | 5 | 5 | 3 | 5 | 5 | 3 | 5 | 5 | 4 | 5 | 4 | 4 |
|  | 15 | Is congenital cataract progressive? | Many more children suffer from partial cataracts that may slowly progress over time, increasing the visual difficulties as the child grows | 5 | 4 | 4 | 5 | 5 | 3 | 5 | 5 | 4 | 5 | 4 | 4 | 5 | 4 | 4 |
| **GD** | 1 | Will the vision improve after congenital cataract surgery? | The management of congenital cataract is very different to the treatment of a routine age-related cataract. In adults, surgery may be delayed for years without affecting the visual outcome. In infants, if the cataract is not removed during the first year of life, the vision will never be fully regained after surgery. | 3 | 3 | 4 | 3 | 2 | 4 | 4 | 4 | 4 | 3 | 3 | 3 | 3 | 3 | 3 |
|  | 2 | If an IOL is implanted now, will it need to be replaced when the individual reaches adulthood? | Unlike the natural lenses of the eye, IOLs do not break down and never need to be replaced. To reap these long-lasting benefits, patients should follow any aftercare instructions as given by their doctor following surgery. Complete recovery can be expected over 1 – 2 days, but this depends on your original eyesight and any underlying health conditions. | 2 | 3 | 3 | 1 | 3 | 3 | 4 | 3 | 3 | 2 | 2 | 3 | 2 | 2 | 3 |
|  | 3 | Is the congenital cataract surgery risky? | Removing a congenital cataract is usually a safe, effective procedure. The child will need follow-up for vision rehabilitation. Most infants with congenital cataract in one eye have some level of "lazy eye" (amblyopia) and will need to use patching after the surgery in an attempt to reverse it. | 3 | 3 | 3 | 2 | 2 | 3 | 4 | 3 | 3 | 3 | 1 | 3 | 2 | 2 | 2 |
|  | 4 | Do we have to proceed with the congenital cataract surgery now, or can we wait? | If the cataracts are present from birth, the operation will be carried out as soon as possible, usually 1 to 2 months after your baby is born. | 3 | 2 | 2 | 2 | 1 | 2 | 4 | 3 | 2 | 3 | 1 | 2 | 3 | 2 | 2 |
|  | 5 | Will congenital cataract redevelop after the surgery? | While your cataracts cannot come back after surgery, you can get what is called posterior capsular opacification (or PCO), also referred to as a secondary cataract. | 3 | 3 | 3 | 2 | 1 | 3 | 4 | 2 | 3 | 3 | 2 | 3 | 3 | 3 | 3 |
|  | 6 | Can we continue with amblyopia training and delay the congenital cataract surgery for a few years? | Deprivation amblyopia may be minimized with early surgery for dense congenital bilateral cataracts. During the first 14 weeks of life, there is a linear relationship between delay in surgery and longterm visual acuity outcome, with an average loss of one line of visual acuity for every three weeks of delay. After 14 weeks of age, up to 31 weeks of age, there is little additional adverse effect of further delay in surgery. | 3 | 2 | 3 | 3 | 1 | 3 | 4 | 3 | 3 | 3 | 2 | 3 | 3 | 2 | 2 |
|  | 7 | Our eldest child has bilateral congenital cataracts, and we are considering having a second child. What is the likelihood of the second child developing cataracts? | The causes of infantile cataracts have been the source of much speculation and research. Making a distinction between unilateral and bilateral cataracts may be useful when considering etiology.  The majority of bilateral congenital or infantile cataracts not associated with a syndrome have no identifiable cause. Genetic mutation is likely the most common cause. Over fifteen genes involved in cataract formation have been identified, and the inheritance is most often autosomal dominant although it can be X-linked or autosomal recessive.Within the same pedigree, there can be considerable morphologic variation.  Systemic associations include metabolic disorders such as galactosemia, Wilson disease, hypocalcemia and diabetes. Cataracts may be a part of a number of syndromes, the most common being trisomy 21. Intrauterine infections including rubella, herpes simplex, toxoplasmosis, varicella and syphilis are another cause.  In contrast, most unilateral cataracts are not inherited or associated with a systemic disease and are of unknown etiology although they do not rule out the possibility of an associated systemic disease. They are usually the result of local dysgenesis and may be associated with other ocular dysgenesis such as persistent fetal vasculature (PFV), posterior lenticonus or lentiglobus.  Trauma is a known cause of pediatric cataracts. If there is no known history of trauma to explain an acquired cataract in this age group, investigation must be considered in children who present with other signs suggestive of child abuse.  Regardless of the etiology, prompt treatment of visually significant cataracts is necessary to allow proper development of vision. | 1 | 2 | 4 | 1 | 3 | 4 | 1 | 2 | 4 | 1 | 1 | 4 | 1 | 3 | 4 |
|  | 8 | After congenital cataract surgery, is it better to wear rigid gas-permeable (RGP) lenses or regular spectacles? | RGPCL is a well-tolerated, safe, and effective treatment for infants and young children. The visual acuity and degree of nystagmus were significantly improved in children who wore RGPCL for aphakia refractive correction after congenital cataract surgery compared with spectacle correction. | 3 | 4 | 4 | 2 | 2 | 3 | 4 | 3 | 4 | 3 | 2 | 3 | 3 | 3 | 3 |
|  | 9 | The child currently has nystagmus. Will the nystagmus resolve after congenital cataract cataract surgery? | Amblyopia, strabismus, and nystagmus which may have developed prior to cataract surgery may continue despite removal of the cataract(s) and must also be addressed. In the postoperative period, it is important to prevent and keep under check any significant inflammatory reaction. | 2 | 2 | 4 | 1 | 2 | 3 | 3 | 3 | 4 | 1 | 1 | 3 | 1 | 2 | 3 |
|  | 10 | We have unilateral congenital cataract in our child. It's said that the surgical outcomes for unilateral cases are not as good. Is this true? | Pseudophakic eyes achieved better final visual acuity than aphakic eyes, and bilateral cataract patients had more favorable visual acuity outcomes than unilateral cataract patients. | 1 | 2 | 4 | 1 | 1 | 3 | 1 | 2 | 3 | 1 | 1 | 3 | 1 | 3 | 3 |
|  | 11 | After congenital cataract surgery in a child, is it necessary to regularly monitor intraocular pressure and axial length? | Elevation in intraocular pressure (IOP) is an early sign of postoperative glaucoma. Glaucoma does not always follow a slow course, as it can develop quickly. However, if affected children are not followed up regularly, the diagnosis may be delayed. | 2 | 3 | 3 | 2 | 1 | 3 | 3 | 2 | 3 | 3 | 2 | 3 | 2 | 3 | 3 |
|  | 12 | How long after congenital cataract surgery should the child avoid physical education classes? | The best guidance regarding when you can exercise after cataract surgery comes from your eye specialist. Be sure to stand by their recommendations since they may have knowledge of your specific situation.  The following timeline provides a basic guideline for when you can resume certain types of physical activity:  Week 1 following surgery  You can engage in low-impact physical activities the week following surgery, such as:  Walking outside;   Light stretching;  Mild treadmill exercise;  Simple household work;  Avoid bending over or lifting anything that weighs more than 10 to 15 pounds, including groceries and laundry. Your eyes may become more stressed as a result, which might prevent appropriate recovery.    Week 2 following surgery  Your doctor might give you the go-ahead for moderate-intensity exercises after two weeks, including:  Yoga;  Jogging;  Brisk walking;  Slow dancing;  4 to 6 weeks following surgery;  You should be able to start doing more strenuous physical activities again. These may consist of:  Lifting weights;  Running;  Swimming;  Hard cycling;  Once more, consult your ophthalmologist before starting this intensity of exercise. | 3 | 3 | 3 | 3 | 3 | 3 | 4 | 3 | 3 | 3 | 4 | 3 | 3 | 4 | 3 |
|  | 13 | Can a child have a multifocal intraocular lens implanted during congenital cataract surgery, or is a monofocal lens the only option? | Monofocal Lens is generally the preferred lens type for babies with congenital cataracts. | 3 | 4 | 4 | 3 | 1 | 3 | 4 | 3 | 3 | 4 | 2 | 3 | 4 | 3 | 4 |
|  | 14 | The severity of congenital cataracts in both of children's eyes is not consistent, should we do cataract surgery in both eyes, or do the severe eye first? | Dense cataracts that are likely to interfere with your baby's vision are usually removed very early, during the first few months of life. If one eye has a denser cataract and the other has a smaller or less dense cataract, then the eye with the denser cataract would usually be treated first. | 3 | 4 | 4 | 3 | 3 | 3 | 4 | 3 | 4 | 3 | 3 | 4 | 3 | 4 | 4 |
|  | 15 | The congenital cataract surgery has been completed, and now amblyopia training is required. However, all amblyopia training programs increase the duration and intensity of eye use, which could lead to a worsening of myopia. Is this a concern? | In congenital/developmental cataract patients who underwent cataract extraction and primary IOL implantation at age 2–3 years, eyes with longer pre-operative ALs had a slower myopic shift than those with average pre-operative ALs 3 years after surgery. This finding could have implications for the target refraction decision in congenital/developmental cataract surgery. | 2 | 2 | 2 | 2 | 1 | 2 | 2 | 2 | 2 | 2 | 1 | 1 | 2 | 2 | 2 |
| **CG** | 1 | What are congenital cataracts? | Congenital cataracts are a condition where a baby is born with clouding of the eye's lens, which can interfere with vision. The lens is a transparent structure inside the eye that helps focus light onto the retina, allowing us to see clearly. When the lens becomes cloudy, light cannot pass through properly, leading to blurred or impaired vision. | 3 | 3 | 4 | 3 | 3 | 3 | 4 | 4 | 4 | 4 | 3 | 4 | 4 | 4 | 4 |
|  | 2 | What is the most common type of congenital cataract? | The most common type of congenital cataract is the "nuclear cataract." This type of cataract affects the central part of the lens, known as the nucleus. Nuclear cataracts often have a characteristic appearance where the central part of the lens becomes cloudy or opaque, which can impact the clarity of vision.  Here are a few key points about nuclear cataracts:  Appearance: Nuclear cataracts appear as a cloudiness or opacity in the center of the lens. In infants, this may be observed as a white or grayish discoloration in the pupil.  Causes: Nuclear cataracts can be caused by genetic factors, and they may be associated with certain inherited conditions or metabolic disorders. They can also occur in isolation without any other health issues.  Detection: In infants and young children, nuclear cataracts are often detected during routine eye exams or when parents notice abnormalities in the child’s vision. Early detection is important to manage the condition effectively.  Treatment: Treatment typically involves surgical removal of the cataractous lens, followed by the use of corrective lenses or other visual aids to support vision development.  While nuclear cataracts are common, there are also other types of congenital cataracts, such as cortical cataracts (which affect the outer edges of the lens) and posterior subcapsular cataracts (which affect the back of the lens). The specific type of cataract and its impact on vision will guide the treatment approach. | 3 | 2 | 4 | 3 | 2 | 4 | 4 | 4 | 4 | 4 | 4 | 4 | 4 | 4 | 4 |
|  | 3 | Why was I born with cataracts? | Congenital cataracts, which are cataracts present at birth or develop shortly after, can be caused by a variety of factors. Sometimes, they occur due to genetic mutations or inherited conditions. Other times, they might be associated with certain infections or exposures that occurred during pregnancy, such as rubella or other illnesses. In some cases, the exact cause isn’t clear.Congenital cataracts can vary in severity and impact on vision. It's important to have regular check-ups to monitor and manage the condition. If you have any concerns or questions about your specific situation, it’s best to discuss them with your ophthalmologist, who can provide personalized information and guidance based on your case. | 5 | 5 | 4 | 5 | 3 | 4 | 5 | 4 | 4 | 5 | 5 | 4 | 5 | 5 | 4 |
|  | 4 | What percent of people have congenital cataracts? | Congenital cataracts are relatively rare, occurring in about 1 to 6 cases per 10,000 live births. This equates to 0.01% to 0.06% of people being born with cataracts. While the exact incidence can vary slightly based on region and access to healthcare, congenital cataracts are considered uncommon compared to other eye conditions. Early detection and treatment, typically in infancy or early childhood, are crucial for preserving vision and preventing long-term visual impairment. | 5 | 5 | 5 | 5 | 4 | 4 | 5 | 5 | 5 | 5 | 5 | 5 | 5 | 5 | 5 |
|  | 5 | What is the difference between congenital cataract and developmental cataract? | Congenital cataracts and developmental cataracts refer to cataracts that occur at different stages of life, and they have distinct characteristics and causes:  Congenital Cataract: Definition: Congenital cataracts are present at birth or develop shortly after birth. Causes: They can result from genetic factors, infections during pregnancy (such as rubella or toxoplasmosis), metabolic disorders, or other conditions affecting the fetus. Characteristics: Congenital cataracts may be present in one or both eyes and can vary in size, opacity, and impact on vision. They are often detected during newborn screening or early in infancy. Management: Treatment typically involves early surgical intervention to remove the cataract and restore clear vision, along with corrective lenses or other aids to support visual development. Developmental Cataract: Definition: Developmental cataracts occur during childhood, usually after the first few months of life, as the child grows and their vision develops. Causes: These cataracts can develop due to various factors, including trauma, certain systemic diseases, or can be idiopathic (with no known cause). They might also be associated with other eye conditions or systemic disorders. Characteristics: Developmental cataracts might not be present at birth but can develop over time as the child grows. They may be more progressive and can affect one or both eyes. Management: The approach to treating developmental cataracts depends on their severity, impact on vision, and underlying cause. Treatment options might include monitoring, corrective lenses, or surgery, depending on how the cataracts affect the child’s vision and overall development. Summary of Differences: Timing: Congenital cataracts are present at or near birth, while developmental cataracts develop later during childhood. Causes: Congenital cataracts are often due to prenatal factors or genetic conditions, whereas developmental cataracts may result from postnatal factors or progress over time. Management: Both types may require surgical intervention and corrective measures, but the timing and specific approach can differ based on when the cataracts are detected and their impact on vision. Understanding these differences helps tailor the approach to diagnosis and treatment, aiming for the best possible visual outcomes for the individual. | 5 | 5 | 4 | 5 | 5 | 4 | 5 | 4 | 4 | 5 | 5 | 4 | 5 | 5 | 4 |
|  | 6 | How serious are congenital cataracts？ | Congenital cataracts can be quite serious, particularly if they are left untreated or not managed appropriately. Their impact largely depends on several factors, including the size and location of the cataract, whether one or both eyes are affected, and the timing of diagnosis and intervention. Here’s an overview of why they can be serious:  Impact on Vision Visual Impairment: Congenital cataracts obstruct light from reaching the retina, which can severely impair vision. If the cataract is large or dense, it can significantly reduce visual acuity or even cause blindness in the affected eye.  Developmental Delays: For infants and young children, vision development is crucial during early years. Untreated cataracts can interfere with normal visual development, leading to issues such as amblyopia (lazy eye), where the brain begins to ignore input from the affected eye.  Potential Complications Amblyopia: This is a common complication of congenital cataracts. If the cataract prevents clear vision, the brain may not develop proper visual pathways, leading to a condition where the affected eye has poor vision despite the presence of a normal eye.  Strabismus: Misalignment of the eyes can occur if the vision is impaired, further complicating visual function and requiring additional treatment.  Visual Development: Congenital cataracts can disrupt normal visual development, which may affect the child’s ability to perform tasks that require good vision, such as reading and writing.  Treatment Considerations Early Intervention: Early diagnosis and treatment are crucial. Surgical removal of the cataract is often necessary to restore vision and prevent developmental issues. The timing of the surgery is critical to support normal visual and cognitive development.  Ongoing Care: After surgery, the child may require corrective lenses or other visual aids, and regular follow-up care is needed to monitor visual development and manage any complications.  Long-term Outcomes With timely and effective treatment, many children with congenital cataracts can achieve good visual outcomes. However, untreated congenital cataracts can lead to permanent vision impairment and developmental delays. The severity of these outcomes can vary based on the individual circumstances and the success of the interventions.  In summary, congenital cataracts can be serious due to their potential to significantly impact vision and visual development. Early detection and appropriate treatment are essential to minimize these risks and support the best possible visual outcomes for the child. | 5 | 5 | 5 | 5 | 5 | 5 | 5 | 4 | 5 | 5 | 5 | 5 | 5 | 5 | 5 |
|  | 7 | What are the long term effects of congenital cataracts? | The long-term effects of congenital cataracts can vary depending on several factors, including the timing of diagnosis, the type and severity of the cataract, and the effectiveness of treatment. Here are some potential long-term effects:  Visual Impairment Reduced Visual Acuity: Even with treatment, some children may experience reduced visual acuity in the affected eye. This can affect their ability to see clearly, especially if the cataract was dense or involved both eyes.  Amblyopia (Lazy Eye): If the cataract was not treated promptly, the affected eye might develop amblyopia, where the brain starts to ignore visual input from that eye. This condition can lead to long-term vision impairment if not addressed with additional therapies.  Visual Development and Function Binocular Vision Issues: Proper alignment and coordination of both eyes can be disrupted, leading to problems with depth perception and binocular vision. Strabismus (eye misalignment) may develop, which could require further treatment.  Difficulty with Low Vision Tasks: Even after cataract surgery, some children may have difficulty with tasks requiring fine visual discrimination, such as reading or participating in sports.  Psychosocial Impact Educational Challenges: Vision issues can impact learning and academic performance. Children might need special accommodations or support in educational settings to help them succeed.  Social and Emotional Effects: Vision problems can affect self-esteem and social interactions. Children with visual impairments may face challenges in social settings and may need additional support to build confidence and social skills.  Need for Ongoing Care Refractive Errors: Post-surgery, children might need corrective lenses to manage refractive errors such as nearsightedness or farsightedness. Regular eye exams are essential to monitor these changes and update prescriptions as needed.  Potential for Further Surgery: In some cases, additional surgeries or treatments may be needed over time to address complications or manage residual vision problems.  Risk of Other Eye Conditions Glaucoma: Children who have had cataract surgery might be at an increased risk of developing glaucoma, a condition where increased pressure in the eye can damage the optic nerve.  Retinal Issues: Depending on the severity of the cataract and the success of surgery, there may be an increased risk of retinal issues that require monitoring.  Quality of Life Adaptation and Support: With appropriate treatment and support, many children adapt well and lead normal lives. Early intervention and ongoing care play a critical role in ensuring the best possible outcomes.  Functional Independence: Many children with congenital cataracts can achieve functional independence and participate in a wide range of activities, though they may need accommodations and support.  Overall, while congenital cataracts can have significant long-term effects, early diagnosis and effective treatment greatly improve the prognosis. Regular follow-up care is essential to manage any ongoing issues and support optimal visual development and quality of life. | 5 | 5 | 5 | 5 | 5 | 4 | 5 | 4 | 5 | 5 | 5 | 4 | 5 | 5 | 4 |
|  | 8 | Does congenital cataract cause amblyopia? | Yes, congenital cataracts can cause amblyopia, often referred to as "lazy eye." Amblyopia is a condition where one eye has poor vision because the brain and eye are not working together properly. Here's how congenital cataracts can lead to amblyopia:  Mechanism Obstruction of Clear Vision: Congenital cataracts cause a clouding of the lens, which obstructs light from reaching the retina. This prevents the affected eye from sending clear visual signals to the brain.  Visual Development: In infants and young children, visual development is crucial during early years. If one eye is not providing clear visual input, the brain may start to favor the other eye. This can lead to a situation where the affected eye does not develop proper visual pathways.  Brain's Adaptation: The brain may begin to ignore or suppress the input from the affected eye because it is receiving less clear or no visual information. This adaptation results in the brain relying predominantly on the stronger eye.  Consequences Reduced Vision: The affected eye develops poorer vision compared to the unaffected eye, even though the eye itself may be structurally normal after treatment.  Difficulty in Binocular Vision: Amblyopia can impact the ability to use both eyes together effectively. This can lead to issues with depth perception and binocular vision.  Prevention and Treatment Early Intervention: Early diagnosis and treatment of congenital cataracts are crucial in preventing or minimizing amblyopia. If a cataract is detected and treated early, there is a better chance of supporting normal visual development and reducing the risk of amblyopia.  Surgical Treatment: Surgery to remove the cataract is often necessary. After surgery, corrective lenses or other visual aids are typically used to help the child achieve clear vision.  Vision Therapy: In cases where amblyopia develops, additional treatments such as vision therapy or patching the stronger eye may be needed to encourage the brain to use the affected eye more effectively.  Ongoing Monitoring: Regular follow-up care is important to monitor visual development and address any issues that may arise.  In summary, congenital cataracts can indeed cause amblyopia if not treated promptly. Early diagnosis and intervention are key to minimizing the risk of amblyopia and supporting optimal visual development. | 5 | 5 | 5 | 5 | 5 | 5 | 5 | 3 | 5 | 5 | 5 | 5 | 5 | 5 | 5 |
|  | 9 | What is the minimum age for congenital cataract surgery? | The minimum age for congenital cataract surgery is not strictly defined by a specific age but rather depends on the individual child's condition and the timing of diagnosis. However, in general practice:  Typical Timing Early Intervention: Ideally, congenital cataract surgery is performed as early as possible to minimize the risk of vision problems. Surgery is often considered within the first few months of life.  Timing for Surgery: Most ophthalmologists aim to perform cataract surgery within the first 6 to 12 weeks of life if the cataract is causing significant visual impairment or is likely to affect visual development.  Factors Influencing Timing Severity of Cataract: The size, density, and location of the cataract can influence the timing of surgery. More severe cataracts may require earlier intervention.  Visual Impact: The potential impact on vision and visual development is a critical factor. If the cataract significantly obstructs vision and affects normal visual development, earlier surgery is prioritized.  Health and Stability of the Infant: The overall health and stability of the infant are also considered. Surgery may be postponed if there are other medical issues that need to be addressed first.  Ophthalmologist’s Recommendations: The decision on timing is guided by the ophthalmologist based on the individual child’s condition and needs.  Benefits of Early Surgery Prevention of Amblyopia: Early surgery helps to prevent or minimize the risk of amblyopia (lazy eye) and supports proper visual development.  Normal Visual Development: Early intervention helps in achieving better visual outcomes and supports normal visual and cognitive development.  Reduction in Long-Term Complications: Performing surgery early can reduce the risk of complications associated with untreated congenital cataracts, such as severe visual impairment and developmental delays.  Follow-Up and Care After surgery, ongoing follow-up care is crucial to monitor the child’s vision and ensure proper visual development. This may include the use of corrective lenses, vision therapy, and regular eye exams to track progress and address any additional issues.  In summary, while there is no fixed minimum age for congenital cataract surgery, it is generally recommended to perform the surgery as early as possible, often within the first few months of life, to optimize visual outcomes and support normal development. | 5 | 5 | 4 | 5 | 4 | 4 | 5 | 4 | 4 | 5 | 4 | 4 | 5 | 5 | 4 |
|  | 10 | What happens if congenital cataracts are left untreated? | If congenital cataracts are left untreated, they can lead to several serious issues, particularly concerning vision development in children. Here’s what might happen:  Visual Impairment: Congenital cataracts can obstruct light from reaching the retina, which is crucial for clear vision. If the cataract is not addressed, it can lead to significant visual impairment or even blindness in the affected eye.  Amblyopia (Lazy Eye): The obstruction caused by the cataract can prevent the brain from receiving clear images from the affected eye. This can result in amblyopia, where the brain starts to ignore input from that eye, leading to poor vision development.  Poor Visual Development: For infants and young children, vision development is a critical process that needs to occur during certain developmental stages. Untreated cataracts can disrupt this process, potentially leading to long-term visual deficits.  Strabismus (Eye Misalignment): The lack of clear vision can lead to eye misalignment or strabismus, where the eyes do not work together properly. This can further complicate vision issues and may require additional treatment.  Impact on Daily Activities: Vision problems resulting from untreated cataracts can affect a child's ability to perform everyday tasks, including reading, writing, and other activities that require good vision.  It’s important to diagnose and treat congenital cataracts as early as possible to minimize these risks and help ensure the best possible visual outcomes for the child. Treatment typically involves surgery to remove the cataract and may include additional measures such as corrective lenses or vision therapy to support optimal visual development. | 5 | 5 | 5 | 5 | 4 | 4 | 5 | 4 | 5 | 5 | 5 | 5 | 5 | 5 | 5 |
|  |  | What is the success rate of congenital cataract surgery? | The success rate of congenital cataract surgery is generally high, especially when the procedure is performed early and followed by appropriate post-operative care. However, the exact success rate can vary based on several factors, including:  1. Timing of Surgery Early Intervention: When congenital cataract surgery is performed early, typically within the first few months of life, the success rate is generally higher. Early surgery helps prevent or minimize issues such as amblyopia and supports normal visual development. 2. Type and Severity of Cataract Cataract Characteristics: The success of the surgery can depend on the size, density, and location of the cataract. More complex or dense cataracts may pose additional challenges. 3. Surgical Technique and Expertise Experienced Surgeons: The skill and experience of the ophthalmic surgeon play a crucial role in the success of the surgery. Surgeons with expertise in pediatric cataract surgery are more likely to achieve favorable outcomes. 4. Post-Operative Care Rehabilitation and Follow-Up: Successful outcomes also depend on proper post-operative care, including the use of corrective lenses, adherence to follow-up appointments, and monitoring for any complications. 5. Individual Factors Overall Health of the Child: The overall health and stability of the child, as well as any other existing health conditions, can influence the success of the surgery. General Success Rates Visual Outcomes: Studies generally show that the majority of children who undergo congenital cataract surgery achieve significant improvement in vision. Reports suggest that approximately 70-80% of children can achieve a functional level of vision with early intervention and appropriate follow-up care.  Complication Rates: While the success rate for improving vision is high, there can be complications, such as secondary glaucoma, lens displacement, or retinal issues. However, with proper management, many of these complications can be addressed effectively.  Long-Term Outcomes Visual Development: Early surgery and follow-up care can lead to good long-term visual outcomes, including the development of normal vision and visual skills. Regular monitoring and management are essential to address any issues that may arise as the child grows. In summary, congenital cataract surgery has a high success rate, particularly when performed early and supported by thorough post-operative care. The majority of children achieve significant improvements in vision, although individual outcomes can vary based on specific factors related to the cataract and the child’s overall health. | 5 | 5 | 5 | 5 | 5 | 5 | 5 | 4 | 5 | 5 | 5 | 5 | 5 | 5 | 5 |
|  | 12 | What is the most common complication of congenital cataract surgery? | The most common complication of congenital cataract surgery is posterior capsule opacification (PCO), also known as "secondary cataract." This occurs when the posterior part of the lens capsule, which is left intact during surgery to support an intraocular lens (IOL), becomes cloudy after cataract removal. In pediatric patients, particularly infants, the risk of PCO is higher because of the aggressive wound-healing response in their eyes. If left untreated, PCO can lead to significant visual impairment. To address this, surgeons often perform a posterior capsulotomy (removal of part of the posterior capsule) during the initial surgery, and sometimes an anterior vitrectomy (removal of some of the gel-like vitreous behind the lens). This helps to reduce the chance of PCO formation and maintain clear visual pathways. | 5 | 5 | 5 | 4 | 4 | 5 | 5 | 3 | 5 | 5 | 4 | 5 | 5 | 5 | 5 |
|  | 13 | What is the surgery of choice for congenital cataract? | The surgery of choice for congenital cataracts is phacoemulsification with intraocular lens (IOL) implantation. This procedure is commonly used due to its effectiveness and precision. Here’s an overview of the process and key aspects:  1. Phacoemulsification Procedure: Phacoemulsification is a technique where the cloudy lens is broken into small fragments using ultrasound waves. These fragments are then suctioned out of the eye. Advantages: This method is minimally invasive and allows for precise removal of the cataract with smaller incisions compared to traditional methods. 2. Intraocular Lens (IOL) Implantation Procedure: After the cataract is removed, an artificial lens, known as an intraocular lens (IOL), is implanted into the eye. This lens helps to restore focus and vision. Types of IOLs: The choice of IOL depends on the child's needs and the surgeon’s recommendations. Options include monofocal lenses, which provide clear vision at one distance, or accommodating lenses that adjust focus for near and far vision. In some cases, if the child is very young or has certain conditions, a contact lens may be used temporarily instead of an IOL. 3. Timing of Surgery Early Intervention: It’s generally recommended to perform the surgery as early as possible, often within the first few months of life, to minimize the risk of visual development issues such as amblyopia (lazy eye) and to support proper visual development. 4. Post-Operative Care Follow-Up: After surgery, regular follow-up visits are essential to monitor the child’s vision, ensure proper healing, and manage any complications. Corrective Measures: The child may need corrective lenses (glasses or contact lenses) to optimize visual acuity after the surgery. 5. Benefits Improved Vision: The primary goal of the surgery is to restore clear vision and support normal visual development. Reduced Complications: Phacoemulsification with IOL implantation is associated with a lower risk of complications compared to older surgical techniques and provides effective visual rehabilitation. In summary, phacoemulsification with intraocular lens implantation is the preferred surgical approach for congenital cataracts due to its effectiveness in removing the cloudy lens and restoring vision. Early intervention and careful post-operative care are crucial to achieving the best outcomes. | 4 | 4 | 4 | 3 | 3 | 4 | 5 | 4 | 4 | 4 | 4 | 4 | 4 | 4 | 4 |
|  | 14 | How are congenital cataracts optically corrected? | Congenital cataracts can be optically corrected through several methods, depending on the specifics of the cataract and the child’s visual needs. Here’s how congenital cataracts are typically managed and corrected optically: 1. Surgical Correction Phacoemulsification and Intraocular Lens (IOL) Implantation Procedure: After cataract removal, an intraocular lens (IOL) is implanted to replace the cloudy lens. This lens helps restore clear vision by focusing light properly onto the retina. Types of IOLs: The choice of IOL depends on the child's age and specific needs. For very young children, an IOL may be placed at the time of cataract surgery, or the child may initially use contact lenses until the eye grows enough to accommodate an IOL. 2. Optical Correction with Lenses Glasses Use: Post-surgery, glasses are often used to correct refractive errors and provide the best possible vision. Glasses can help manage any residual refractive issues such as nearsightedness (myopia), farsightedness (hyperopia), or astigmatism. Customization: The prescription for glasses will be tailored based on the child’s specific vision needs and any changes in vision following surgery. Contact Lenses Use: In some cases, particularly if IOL implantation isn’t possible or as a temporary measure before the eye grows enough for an IOL, contact lenses may be used. They provide a more flexible solution for vision correction and can be adjusted as needed. Benefits: Contact lenses can provide a wider field of vision and reduce issues related to the fit of glasses. 3. Vision Therapy Rehabilitation Use: In cases where there are issues such as amblyopia (lazy eye) or difficulties with binocular vision, vision therapy or patching may be recommended. This helps to improve the coordination between the eyes and encourages the use of the affected eye. Techniques: Therapy may include exercises to strengthen eye muscles and improve visual skills. 4. Follow-Up Care Regular Monitoring Importance: Ongoing follow-up visits with an ophthalmologist are essential to monitor visual development, adjust optical prescriptions, and address any complications or changes in vision. Adjustments: Regular eye exams help ensure that any adjustments to glasses or contact lenses are made as needed to support optimal vision. Summary Optical correction for congenital cataracts involves a combination of surgical intervention, such as phacoemulsification with IOL implantation, and post-surgical correction with glasses or contact lenses. Vision therapy may be used to address additional visual development issues. Regular follow-up and monitoring are crucial to ensure the best visual outcomes and adjust treatments as needed. | 5 | 5 | 5 | 5 | 4 | 4 | 5 | 4 | 5 | 5 | 5 | 5 | 5 | 5 | 5 |
|  | 15 | Is congenital cataract progressive? | Congenital cataracts are typically not progressive in the sense that they do not usually worsen over time once they are present. However, there are some important nuances to consider: 1. Nature of Congenital Cataracts Stable After Birth: Once a congenital cataract is formed, it generally remains stable in terms of its opacification. Unlike age-related cataracts, which tend to worsen progressively over time, congenital cataracts don’t usually change significantly in density or size after birth. 2. Potential for Complications Progressive Visual Issues: While the cataract itself may not progress, untreated congenital cataracts can lead to progressive visual issues due to their impact on vision development. For instance: Amblyopia (Lazy Eye): If a congenital cataract obstructs vision, it can cause amblyopia, where the affected eye's vision does not develop normally. This can lead to worsening visual function if not addressed. Strabismus (Eye Misalignment): Ongoing visual impairment can lead to strabismus, which may worsen if the visual issues are not corrected. 3. Surgical and Post-Surgical Considerations Surgical Outcomes: After cataract surgery, while the cataract itself is removed, some children may develop other issues such as posterior capsule opacification (PCO), where the membrane behind the lens becomes cloudy. This condition can affect vision but is typically treatable with a minor laser procedure. Vision Development: Even after successful cataract removal and optical correction, some children may face challenges related to visual development. Ongoing monitoring and adjustments are necessary to manage these issues effectively. 4. Management and Follow-Up Regular Monitoring: Regular eye exams are essential to monitor for any changes in vision and to ensure that optical corrections (glasses, contact lenses) are appropriate. This helps address any new or arising visual issues promptly. In summary, while congenital cataracts themselves are not typically progressive once formed, their impact on vision can lead to secondary issues that may progress if not treated. Early diagnosis and timely surgical intervention are crucial to preventing these complications and supporting normal visual development. Regular follow-up care is important to manage any potential post-surgical or developmental issues. | 4 | 4 | 5 | 5 | 4 | 5 | 5 | 5 | 5 | 5 | 5 | 5 | 4 | 4 | 5 |
| **CD** | 1 | Will the vision improve after congenital cataract surgery? | Yes, vision often improves significantly after congenital cataract surgery, especially if the cataract was obstructing vision. The primary goal of the surgery is to remove the cloudy lens and replace it with a clear artificial intraocular lens (IOL) or to use contact lenses or glasses to correct vision if an IOL isn't suitable.  Here are a few key points to consider:  Early Intervention: The earlier the surgery is performed, the better the visual outcomes tend to be. Early intervention helps in the proper development of visual pathways and reduces the risk of amblyopia (lazy eye) and other complications.  Rehabilitation: Post-surgery, vision improvement may take some time as the brain adjusts to the new visual input, especially in very young children. Vision therapy and regular follow-up care are important.  Visual Outcomes: While many individuals experience significant improvement in vision after surgery, the final outcome can vary based on factors such as the severity of the cataract, the presence of any other eye conditions, and how well the brain adapts to the corrected vision.  Your ophthalmologist will provide guidance on what to expect and how to support the best possible visual outcomes following surgery. | 3 | 4 | 4 | 4 | 4 | 4 | 5 | 4 | 4 | 5 | 4 | 4 | 4 | 4 | 4 |
|  | 2 | If an IOL is implanted now, will it need to be replaced when the individual reaches adulthood? | In most cases, once an intraocular lens (IOL) is implanted, it doesn’t need to be replaced as the person grows older. IOLs are designed to be durable and to last a lifetime. However, there are a few exceptions and considerations:  Lens Power Changes: If the child’s eye continues to grow significantly after the IOL is implanted, the power of the lens might no longer be optimal. This might necessitate a lens replacement or adjustment in adulthood, but it's relatively rare.  Complications or Changes: If there are complications or if the lens becomes dislocated or causes other issues, it might need to be replaced or adjusted.  Advances in Technology: In some cases, advancements in IOL technology might offer improved options, and a replacement could be considered for enhanced vision or comfort.  Regular follow-up appointments with your ophthalmologist will help ensure that the IOL continues to meet your vision needs and address any issues that might arise. | 5 | 5 | 5 | 5 | 4 | 5 | 5 | 4 | 5 | 5 | 4 | 5 | 5 | 5 | 5 |
|  | 3 | Is the congenital cataract surgery risky? | Congenital cataract surgery is generally safe and effective, but, like any surgical procedure, it does come with some risks and potential complications. Here's a summary of what you might expect:  Risks and Complications: Infection: As with any surgery, there's a risk of infection, though this is relatively rare due to modern sterile techniques and antibiotics.  Inflammation: Post-operative inflammation can occur, but it's usually manageable with medication.  Glaucoma: Increased eye pressure can sometimes develop after surgery, but this is usually monitored and treated if necessary.  Retinal Detachment: Though rare, there's a slight risk of retinal detachment, especially in young children whose eyes are still developing.  Lens Dislocation: The intraocular lens (IOL) could become dislocated, which may require additional surgery.  Visual Outcomes: While many patients achieve excellent vision post-surgery, some may still have visual issues or require additional interventions such as glasses, contact lenses, or further surgeries.  Benefits and Considerations: Improved Vision: The primary benefit is a significant improvement in vision, especially if the cataract was impairing sight.  Early Intervention: Performing surgery at an early age can help prevent or minimize the impact on visual development and reduce the risk of complications such as amblyopia.  Long-term Monitoring: Regular follow-up appointments are crucial to monitor for any issues and to ensure the best possible visual outcomes.  Overall, congenital cataract surgery is a well-established procedure with a high success rate. Discussing the potential risks and benefits with your ophthalmologist can help you make an informed decision and prepare for the recovery process. | 4 | 4 | 4 | 5 | 4 | 4 | 4 | 4 | 4 | 5 | 3 | 3 | 4 | 4 | 4 |
|  | 4 | Do we have to proceed with the congenital cataract surgery now, or can we wait? | Deciding whether to proceed with congenital cataract surgery immediately or to wait involves several important considerations. Here’s a general overview to help you understand the factors that might influence this decision:  Reasons to Consider Early Surgery: Visual Development: Early intervention is crucial for proper visual development. The visual pathways in the brain are still developing in infants and young children. If a congenital cataract is left untreated, it can lead to long-term vision problems, such as amblyopia (lazy eye) or poor visual acuity.  Preventing Complications: Untreated cataracts can cause additional complications, such as strabismus (crossed eyes) or further visual impairment, which might be harder to manage later.  Improved Outcomes: Studies have shown that earlier surgery often leads to better visual outcomes and reduces the need for additional corrective measures later on.  Factors to Consider Before Deciding to Wait: Severity of the Cataract: The urgency of surgery can depend on the severity of the cataract and how much it is affecting vision. If the cataract is not significantly impairing vision or causing other issues, your ophthalmologist might recommend monitoring the situation.  Age and Overall Health: The child’s overall health and readiness for surgery are important. In some cases, your ophthalmologist might recommend waiting if they believe that the child is too young or if there are other health considerations.  Potential for Natural Resolution: In rare cases, congenital cataracts might have the potential to improve on their own, although this is not common.  Making the Decision: Consultation: It’s important to have a detailed discussion with your ophthalmologist about the specific characteristics of the cataract, the potential risks of waiting, and the benefits of early surgery.  Regular Monitoring: If the decision is made to wait, regular eye exams are crucial to monitor any changes in the cataract and to ensure that it is not adversely affecting the child's vision.  Ultimately, the decision will be based on the specific details of the cataract and the child's visual needs. Your ophthalmologist can provide personalized advice and help you make the best choice for your child’s health and development. | 5 | 5 | 4 | 5 | 5 | 4 | 4 | 3 | 3 | 5 | 4 | 3 | 5 | 4 | 3 |
|  | 5 | Will congenital cataract redevelop after the surgery? | Congenital cataracts typically do not redevelop after surgery, but there are some important considerations to be aware of:  Possible Post-Surgical Complications: Posterior Capsule Opacification (PCO): Sometimes, after the removal of a congenital cataract, the thin membrane (capsule) that holds the artificial lens in place can become cloudy or opacified. This is not a reoccurrence of the original cataract but a new issue that can affect vision. PCO can be treated with a relatively simple outpatient procedure called YAG laser capsulotomy.  Lens Dislocation or Opacification: In rare cases, the intraocular lens (IOL) that is implanted during surgery can become dislocated or develop problems, which might affect vision. This is not the cataract returning but a different issue that might require intervention.  Other Eye Conditions: If there were underlying or associated eye conditions, they may still require attention and monitoring after the cataract surgery.  Long-Term Monitoring: Regular Follow-Up: Even after successful cataract surgery, regular eye exams are important to monitor the health of the eye and the performance of the IOL.  Visual Development: Especially in children, continuous follow-up is crucial for assessing visual development and addressing any issues that may arise as the child grows.  While the risk of the original congenital cataract redeveloping is not present, ongoing eye care and monitoring are essential to ensure the best visual outcomes and to address any new concerns that might arise. | 4 | 4 | 4 | 3 | 3 | 4 | 4 | 3 | 3 | 5 | 3 | 3 | 5 | 5 | 4 |
|  | 6 | Can we continue with amblyopia training and delay the congenital cataract surgery for a few years? | While amblyopia (lazy eye) training can be a crucial part of managing visual development, it generally cannot replace the need for congenital cataract surgery if the cataract is significantly impairing vision. Here’s why:  Importance of Early Surgery: Visual Development: For infants and young children, the primary goal is to prevent or minimize visual impairment. Cataracts that obstruct vision can interfere with the development of the visual pathways in the brain. Early removal of the cataract helps ensure proper visual development and reduces the risk of amblyopia.  Amblyopia Prevention: If a congenital cataract is left untreated, it can lead to amblyopia, where one eye becomes weaker than the other. Amblyopia treatment, such as patching or vision therapy, is more effective when initiated early, but it works best when the cataract is already removed.  Risks of Delaying Surgery: Progressive Visual Impairment: Delaying surgery can lead to progressive vision loss and make the treatment of amblyopia more challenging. The longer a cataract obstructs vision, the harder it may be to achieve optimal visual outcomes, even with amblyopia training.  Complications: Untreated cataracts can lead to additional complications, such as strabismus (crossed eyes) or further visual impairment, which might complicate treatment and management later on.  Possible Approach: Consult Your Ophthalmologist: It’s essential to discuss the specific details of the cataract and your child’s visual needs with your ophthalmologist. They can provide personalized advice based on the severity of the cataract and the potential impact on vision.  Combine Strategies: In some cases, an ophthalmologist might recommend proceeding with surgery while also continuing amblyopia training to address any visual development issues that arise post-surgery.  In general, early surgery is often recommended to prevent the complications associated with congenital cataracts and to provide the best foundation for visual development. Your ophthalmologist can help you weigh the benefits of early surgery against the potential advantages of any other treatments or delays. | 3 | 4 | 4 | 3 | 3 | 4 | 3 | 3 | 3 | 3 | 3 | 3 | 4 | 4 | 4 |
|  | 7 | Our eldest child has bilateral congenital cataracts, and we are considering having a second child. What is the likelihood of the second child developing cataracts? | The likelihood of a second child developing congenital cataracts can be influenced by several factors, including genetic and environmental considerations. Here’s a general overview:  Genetic Factors: Inherited Conditions: If the congenital cataracts in your eldest child are due to a genetic condition, there may be an increased risk for the second child. Some genetic disorders associated with congenital cataracts can be passed from parents to children. In such cases, genetic counseling might be recommended to assess the risk more accurately.  Genetic Counseling: Consulting with a genetic counselor can help you understand the risk based on your family's specific genetic background and any known hereditary conditions. They can provide information on the likelihood of recurrence and the available options for screening or early intervention.  Environmental and Other Factors: Non-Genetic Causes: Congenital cataracts can also result from non-genetic factors, such as prenatal infections (e.g., rubella) or exposure to certain substances during pregnancy. If your eldest child’s cataracts were due to such factors, the risk to a second child might be lower, but it’s still important to maintain good prenatal care and avoid known risks.  Individual Risk Assessment: Each pregnancy is unique, and the risk of congenital cataracts in a second child may be different from that in the first child. Factors such as the health of the pregnancy and any prenatal issues should be monitored by your healthcare provider.  Next Steps: Consult Your Healthcare Provider: Speak with your obstetrician or a genetic counselor about your family’s history and any concerns you may have. They can provide personalized advice based on your specific situation.  Prenatal Care: Ensure that you receive regular prenatal care to monitor the health of the pregnancy and to address any potential risks.  Overall, while there may be a higher risk if the congenital cataracts were due to a genetic condition, each pregnancy is unique. Consulting with a specialist will provide the most accurate information and help you make informed decisions. | 5 | 5 | 5 | 5 | 5 | 5 | 4 | 2 | 4 | 5 | 3 | 4 | 5 | 5 | 5 |
|  | 8 | After congenital cataract surgery, is it better to wear rigid gas-permeable (RGP) lenses or regular spectacles? | After congenital cataract surgery, the choice between rigid gas-permeable (RGP) lenses and regular spectacles depends on several factors, including the specific needs of the patient, the quality of vision achieved with the intraocular lens (IOL), and personal comfort. Here’s a comparison to help understand the options:  Rigid Gas-Permeable (RGP) Lenses: Advantages:  Visual Acuity: RGP lenses can provide excellent visual acuity, especially if there are irregularities in the cornea or if the IOL power is not perfectly corrected with spectacles. Stability: They offer a stable and precise optical correction, which can be beneficial if there is any irregular astigmatism. Durability: RGP lenses are generally durable and resistant to scratching. Disadvantages:  Comfort: They can be less comfortable than soft contact lenses or spectacles, particularly for people who are not accustomed to wearing them. Adaptation: Some people may require a period of adaptation to get used to wearing RGP lenses. Regular Spectacles: Advantages:  Ease of Use: Spectacles are easy to use and don’t require direct contact with the eye, making them a non-invasive option. Comfort: Many people find spectacles more comfortable than contact lenses. Less Maintenance: They are easier to clean and maintain compared to contact lenses. Disadvantages:  Vision Correction Limits: Spectacles may not correct certain types of vision issues as effectively as contact lenses, especially if there are significant visual distortions or irregularities. Peripheral Vision: Spectacles might not provide as wide a field of view as contact lenses. Factors to Consider: Visual Needs: If the child has specific visual needs or issues like astigmatism that are not fully corrected with spectacles, RGP lenses might be a better option.  Comfort and Lifestyle: Consider the comfort and lifestyle of the child. Young children, for example, might find it easier to adapt to spectacles rather than contact lenses.  Follow-Up Care: Regular follow-up appointments with the ophthalmologist or optometrist are crucial to ensure that the chosen corrective method is providing optimal vision and comfort.  Conclusion: Both RGP lenses and spectacles have their benefits and drawbacks. The decision often depends on the individual’s specific visual needs, comfort, and lifestyle preferences. Your ophthalmologist or optometrist can provide personalized recommendations based on the results of the cataract surgery and the child’s visual requirements. | 4 | 4 | 5 | 4 | 4 | 4 | 5 | 4 | 5 | 5 | 5 | 5 | 5 | 5 | 5 |
|  | 9 | The child currently has nystagmus. Will the nystagmus resolve after congenital cataract cataract surgery? | Nystagmus, which is characterized by involuntary, rapid eye movements, may improve, remain the same, or in some cases worsen after congenital cataract surgery. The impact of the surgery on nystagmus depends on various factors:  Potential Outcomes of Congenital Cataract Surgery on Nystagmus: Improvement:  Visual Clarity: If the cataracts were significantly obstructing vision, removing them can improve visual clarity. Enhanced visual input may lead to some reduction in nystagmus, particularly if the nystagmus was partly due to poor visual input. Reduced Visual Deficits: Improved vision can help in stabilizing the visual system, which might lead to a reduction in the intensity of nystagmus. No Change:  Underlying Conditions: If the nystagmus is due to underlying neurological or structural issues that are not related to the cataract, the surgery may not have a significant impact on it. Worsening:  Adaptation Phase: Occasionally, after surgery, there might be a period of visual adaptation where nystagmus could temporarily worsen before improving, especially if the visual system is adjusting to the new input from the corrected vision. Management and Follow-Up: Post-Surgical Monitoring: After cataract surgery, it's essential to monitor the child's vision and nystagmus closely. Regular follow-up appointments with the ophthalmologist are necessary to assess the outcomes and make any required adjustments.  Additional Therapies: If nystagmus persists or remains problematic after surgery, additional therapies may be considered. These can include vision therapy, corrective lenses, or even surgical options depending on the specific case.  Multidisciplinary Approach: In some cases, a multidisciplinary approach involving pediatric ophthalmologists, neurologists, and vision therapists may be needed to address nystagmus comprehensively.  In summary, while cataract surgery can improve visual acuity and potentially have a positive effect on nystagmus, it does not guarantee that nystagmus will resolve. Close monitoring and additional interventions might be required based on the child's specific condition and response to the surgery. | 5 | 5 | 5 | 5 | 5 | 5 | 4 | 3 | 4 | 5 | 4 | 4 | 5 | 4 | 5 |
|  | 10 | We have unilateral congenital cataract in our child. It's said that the surgical outcomes for unilateral cases are not as good. Is this true? | The surgical outcomes for unilateral congenital cataracts can indeed be different from those for bilateral cases, but they are not necessarily worse. Several factors influence the success of surgery and the final visual outcomes:  Factors Affecting Outcomes: Visual Development:  Visual Dominance: In unilateral cases, the eye with the cataract may have been deprived of clear vision for a period, which can affect visual development. If the cataract is removed early, the brain may adapt better, but outcomes can still be influenced by the duration of visual deprivation before surgery. Amblyopia:  Risk of Amblyopia: The eye with the cataract is at risk of developing amblyopia (lazy eye) due to the lack of clear visual input. Successful treatment requires not only cataract removal but also careful management of amblyopia with therapies such as patching or vision therapy. Surgical Technique and Lens Choice:  Intraocular Lens (IOL) Placement: The choice of IOL and the precision of its placement are crucial. In unilateral cases, the focus and alignment of the IOL must be carefully managed to provide the best visual outcome. Rehabilitation:  Post-Surgery Rehabilitation: Post-operative care, including visual rehabilitation and correction with glasses or contact lenses, plays a significant role in achieving the best visual outcome. Outcomes and Expectations: Visual Acuity: With timely and appropriate surgery, many children with unilateral congenital cataracts achieve good visual acuity. However, the final visual outcome can vary based on factors like the age at surgery, presence of amblyopia, and effectiveness of post-surgical therapies.  Adaptation: The brain and visual system can adapt well, especially if surgery is performed early. This adaptation can help in achieving near-normal vision, though some degree of visual disparity between the two eyes may persist.  Conclusion: While unilateral congenital cataracts can present unique challenges, especially concerning visual development and amblyopia, many children achieve excellent outcomes with proper surgical intervention and post-surgical care. The key to success is early diagnosis and treatment, as well as comprehensive follow-up to address any arising issues and support optimal visual development. Consulting with a pediatric ophthalmologist can provide a more personalized outlook based on your child’s specific situation and needs. | 5 | 4 | 5 | 5 | 3 | 4 | 5 | 4 | 4 | 5 | 3 | 4 | 5 | 4 | 4 |
|  |  | After congenital cataract surgery in a child, is it necessary to regularly monitor intraocular pressure and axial length? | Yes, after congenital cataract surgery in a child, regular monitoring of intraocular pressure (IOP) and axial length is important for several reasons:  Monitoring Intraocular Pressure (IOP): Glaucoma Risk: Post-surgical patients, including children, are at risk of developing glaucoma. This condition can be caused by various factors, including inflammation, changes in eye anatomy, or the use of certain medications. Regular IOP checks help in the early detection and management of glaucoma.  Post-Surgical Complications: Elevated IOP can also be a sign of complications such as inflammation or steroid response, which need to be addressed promptly to prevent long-term damage.  Monitoring Axial Length: Growth and Development: In children, the eye continues to grow and develop, even after cataract surgery. Monitoring axial length helps track this growth and ensures that any changes are managed appropriately. This is crucial for adjusting vision correction strategies, such as lenses or glasses.  Lens Power Adjustment: Changes in axial length can affect the power needed for optimal vision correction. Regular measurements help in fine-tuning corrective lenses or determining the need for additional interventions.  Follow-Up Schedule: Post-Surgery Visits: Frequent follow-up visits are essential, especially in the early months and years after surgery. Your ophthalmologist will typically schedule these visits to monitor IOP, axial length, and overall visual development.  Long-Term Care: Ongoing monitoring is crucial as the child grows. The frequency of visits may decrease over time but should be maintained to ensure that any issues are identified and addressed promptly.  Conclusion: Regular monitoring of both intraocular pressure and axial length after congenital cataract surgery is a key part of comprehensive care. It helps manage potential complications, track the child's visual development, and ensure that any necessary adjustments to vision correction are made. Your ophthalmologist will guide you on the appropriate schedule for these evaluations based on your child's specific needs and the details of the surgery. | 5 | 5 | 5 | 5 | 5 | 5 | 4 | 4 | 5 | 5 | 5 | 5 | 5 | 5 | 5 |
|  | 12 | How long after congenital cataract surgery should the child avoid physical education classes? | The duration for which a child should avoid physical education (PE) classes or strenuous activities after congenital cataract surgery can vary based on several factors, including the child’s overall health, the specifics of the surgery, and the ophthalmologist's recommendations. Here’s a general guideline:  Immediate Post-Surgery Period: First Few Weeks: It is typically advised that children avoid vigorous activities and physical education classes for at least 4 to 6 weeks after congenital cataract surgery. This period allows the eye to heal properly and minimizes the risk of complications such as dislodging the intraocular lens (IOL) or causing trauma to the eye.  Initial Healing: During the first few weeks, the focus is on ensuring that the eye heals well. Activities that involve heavy lifting, running, or jumping should be avoided. Gentle activities and a gradual return to physical activity can be considered based on the child’s recovery and the ophthalmologist’s advice.  Follow-Up and Gradual Return: Follow-Up Appointments: Regular follow-up visits with the ophthalmologist will assess the healing progress and determine when it is safe for the child to resume more vigorous activities. The ophthalmologist will provide specific guidelines based on the individual case.  Gradual Resumption: After the initial healing period, physical activities can typically be resumed gradually. The ophthalmologist might recommend starting with less strenuous activities and progressively increasing the intensity as long as there are no signs of complications or issues.  Protective Measures: If the child is involved in sports or activities that pose a risk of eye injury, additional protective measures such as wearing safety goggles or eye shields may be recommended.  Individual Considerations: Surgical Details: The specifics of the surgery, such as whether there were any complications or additional procedures, may affect the recommended period for avoiding physical activities.  Child’s Condition: The child’s overall health, comfort level, and healing progress should also be considered when determining when to resume physical education classes.  Conclusion: While a general guideline suggests avoiding strenuous physical activities for about 4 to 6 weeks after congenital cataract surgery, the best approach is to follow the specific recommendations of your child’s ophthalmologist. They will provide tailored advice based on the child’s surgical outcome and overall recovery. | 4 | 4 | 5 | 4 | 4 | 5 | 5 | 4 | 5 | 5 | 4 | 5 | 5 | 5 | 5 |
|  | 13 | Can a child have a multifocal intraocular lens implanted during congenital cataract surgery, or is a monofocal lens the only option? | The choice between a multifocal intraocular lens (IOL) and a monofocal IOL for congenital cataract surgery in children generally leans towards monofocal lenses, especially in very young children. Here’s why:  Monofocal IOLs: Simplicity and Safety: Monofocal IOLs are the standard choice for children undergoing congenital cataract surgery. They provide a single, fixed focus for either near or distance vision. They are simpler and have a well-established safety profile, which is crucial in the context of pediatric cataract surgery.  Predictability: Monofocal lenses allow for more predictable outcomes and easier post-surgical adjustments. This predictability is important when dealing with the dynamic visual development of children.  Visual Development: In young children, visual development is still ongoing. A monofocal IOL can be set to focus at a distance, and the child can use corrective lenses for near vision as needed. This approach can be more manageable and adaptable as the child grows.  Multifocal IOLs: Complexity: Multifocal IOLs, which provide multiple focal points for near, intermediate, and distance vision, are more complex. They may introduce issues such as reduced contrast sensitivity and visual disturbances, which might be challenging for a child.  Adaptation: Multifocal lenses require a degree of adaptation, and their benefits are generally more suited to adults or older children who have more mature visual systems and a stable visual demand.  Less Common in Pediatrics: Multifocal IOLs are less commonly used in pediatric cases due to the potential for complications and the need for precise visual outcomes. Most pediatric ophthalmologists prefer monofocal lenses for the simplicity and reliability they offer.  Special Considerations: Age and Development: For very young children, who are still undergoing significant visual development, monofocal lenses are usually preferred. For older children, who are closer to full visual maturity, discussions about multifocal lenses might be more relevant, but they are still less common.  Consultation with Specialists: If considering multifocal lenses, it's crucial to consult with a pediatric ophthalmologist who has experience with advanced lens options in children. They can provide guidance based on the child’s specific needs, visual requirements, and overall eye health.  Conclusion: In most cases, a monofocal IOL is the preferred choice for congenital cataract surgery in children due to its predictability and safety. Multifocal IOLs are typically reserved for adult patients or older children with stable visual needs. Discussing your child's specific situation with a pediatric ophthalmologist will help determine the most appropriate lens option for their needs. | 5 | 4 | 4 | 3 | 5 | 4 | 4 | 3 | 4 | 5 | 4 | 4 | 5 | 4 | 4 |
|  | 14 | The severity of congenital cataracts in both of children's eyes is not consistent, should we do cataract surgery in both eyes, or do the severe eye first? | When dealing with congenital cataracts of varying severity in both eyes, the approach to surgery generally involves careful consideration of several factors. Here’s a detailed look at the typical approach:  General Approach to Surgery: Prioritize the Severe Eye:  Immediate Impact: If one eye has a more severe cataract that significantly impairs vision, it is often prioritized for surgery. The rationale is to address the more severe visual impairment first to prevent further damage to visual development. Visual Development: Early intervention in the more severely affected eye can help reduce the risk of developing amblyopia (lazy eye) and improve overall visual outcomes. Assess and Plan for the Second Eye:  Staging: After addressing the more severely affected eye, the next step is to evaluate the less affected eye. This typically involves regular monitoring to assess whether and when intervention might be needed. Timing: The timing of surgery for the second eye can depend on factors such as how the visual development is progressing and whether the less affected eye begins to experience more issues. Factors to Consider: Visual Impairment: The degree to which each cataract affects vision is a critical factor. The more significant the visual impairment in one eye, the higher the priority for surgery.  Age and Development: The age of the child and their visual development stage are crucial. Early surgery in the more severely affected eye is essential to promote normal visual development.  Surgical Risks and Benefits: Each surgery carries risks, and managing these risks is crucial. Performing surgery on the most severely affected eye first allows for better planning and management of the child’s recovery and rehabilitation.  Post-Surgical Monitoring: Close follow-up is necessary to monitor the outcomes of the initial surgery and to determine the best time for addressing the less affected eye. The visual and overall health of the eye should be closely watched.  Consultation and Decision-Making: Pediatric Ophthalmologist: Consult with a pediatric ophthalmologist who can assess the specific situation of your children’s cataracts and recommend a personalized treatment plan. They will consider the severity of each cataract, the potential impact on visual development, and the best approach to achieve optimal outcomes.  Individualized Plan: The decision on whether to operate on both eyes simultaneously or in stages will be based on an individualized plan tailored to the child's needs, taking into account the severity of the cataracts and the overall health of the eyes.  Conclusion: In most cases, the more severely affected eye is prioritized for surgery to address significant visual impairment and reduce the risk of amblyopia. The less affected eye is then monitored and managed accordingly. This staged approach helps in managing risks and optimizing visual outcomes. Your pediatric ophthalmologist will provide the best guidance based on the specific details of your children’s conditions. | 5 | 4 | 5 | 5 | 5 | 4 | 5 | 4 | 4 | 5 | 5 | 4 | 5 | 5 | 4 |
|  | 15 | The congenital cataract surgery has been completed, and now amblyopia training is required. However, all amblyopia training programs increase the duration and intensity of eye use, which could lead to a worsening of myopia. Is this a concern? | Amblyopia training, often referred to as vision therapy or patching, is an essential part of treating amblyopia (lazy eye) after congenital cataract surgery. The concern about worsening myopia (nearsightedness) due to increased eye use during amblyopia training is understandable, but here’s how it can be managed:  Key Points Regarding Amblyopia Training and Myopia: Purpose of Amblyopia Training:  Visual Development: The primary goal of amblyopia training is to improve the visual acuity of the weaker eye and encourage proper visual development. This training often involves activities that promote visual engagement and strengthen the eye's focus and coordination. Myopia Considerations:  Monitoring and Management: While increased use of the eyes can potentially affect refractive errors like myopia, the benefits of amblyopia treatment generally outweigh these risks. Regular monitoring by your ophthalmologist or optometrist can help manage any changes in refractive error. Adjustments: If myopia progression is noted, corrective measures such as adjusting glasses prescriptions or incorporating specialized lenses (e.g., orthokeratology or myopia control lenses) can be considered. Balanced Approach:  Structured Programs: A structured and supervised amblyopia training program helps ensure that the duration and intensity of eye use are appropriate for the child’s condition. This reduces the risk of overuse and helps balance the need for visual stimulation with overall eye health. Individualized Treatment: The amblyopia training plan should be tailored to the child’s specific needs and capabilities. Your healthcare provider will adjust the training as necessary to avoid excessive strain on the eyes. Regular Follow-Up:  Eye Exams: Regular follow-up visits with the ophthalmologist or optometrist are crucial to monitor the progress of amblyopia treatment and any changes in myopia. This allows for timely adjustments to the treatment plan and corrective measures if needed. Additional Measures:  Myopia Control Strategies: If myopia progression becomes a concern, additional strategies might be employed to control its progression, such as myopia management lenses, atropine eye drops, or lifestyle modifications. Conclusion: While there is a potential concern that increased eye use during amblyopia training could impact myopia, this risk is typically managed through careful monitoring and individualized treatment plans. The benefits of amblyopia training in improving visual acuity and development usually outweigh the potential for myopia progression. Regular follow-up with your child’s eye care provider ensures that any issues are promptly addressed and that the treatment plan remains effective and appropriate for your child’s needs. | 5 | 5 | 5 | 5 | 5 | 5 | 5 | 4 | 5 | 5 | 4 | 5 | 5 | 5 | 5 |
| **DG1** | 1 | What are congenital cataracts? | Congenital cataracts refer to cataracts that are either present at birth or develop shortly after due to genetic or developmental abnormalities. This condition is relatively common in children and is one of the leading causes of childhood blindness and vision impairment. | 4 | 4 | 5 | 4 | 4 | 4 | 5 | 4 | 4 | 4 | 4 | 4 | 4 | 5 | 5 |
|  | 2 | What is the most common type of congenital cataract? | The classification of congenital cataracts includes many types based on severity, location of onset, and morphology. Among them, the most common type is perinuclear congenital cataract. | 5 | 4 | 4 | 5 | 4 | 4 | 5 | 3 | 4 | 5 | 4 | 3 | 5 | 5 | 4 |
|  | 3 | Why was I born with cataracts? | The causes of congenital cataracts can be grouped into three main categories: genetic factors, environmental factors, and unknown causes.  1.Genetic Factors: Approximately one-third of congenital cataract cases are due to genetic causes. There are three primary patterns of inheritance: autosomal dominant (AD), autosomal recessive (AR), and X-linked recessive (XR), with autosomal dominant inheritance being the most common. Due to the complexity of genetics, mutations in the same gene can lead to different clinical presentations, and the same clinical presentation can result from mutations in different genes. Currently, congenital cataracts are associated with four main categories of genetic mutations:  (1) Structural abnormalities in lens proteins, leading to improper arrangement of lens fibers (e.g., CRYAA, CRYBA, CRYG).  (2) Changes in membrane proteins within the lens, affecting normal communication between cells and leading to cloudiness (e.g., gap junction protein alpha).  (3) Abnormal expression of regulatory and metabolic proteins involved in lens development (e.g., homeobox genes 3 and 6).  (4) Mutations in heat shock protein 4 (HSF4), which are also linked to cataract formation.  2.Environmental Factors: Congenital cataracts can also be caused by environmental factors, such as:  (1) Viral infections in the mother during the first trimester of pregnancy, including rubella, measles, chickenpox, and mumps, as well as exposure to certain chemicals, X-rays, or poor maternal nutrition during pregnancy.  (2) Premature birth, low birth weight, and prolonged oxygen therapy after birth, all of which can disrupt normal lens development and lead to cataracts.  3.Unknown Causes: In many cases, the exact cause cannot be identified, and these cases are considered sporadic. They are not clearly linked to genetic or environmental factors. | 5 | 5 | 5 | 5 | 5 | 5 | 4 | 2 | 5 | 5 | 3 | 5 | 5 | 5 | 5 |
|  | 4 | What percent of people have congenital cataracts? | Currently, the global prevalence of congenital cataracts is around 4%, while in China it is approximately 0.5%. Among newborns, the incidence is 2.2 to 3 cases per 10,000 births. Worldwide, about 1.5 million children have low vision or are blind due to congenital cataracts. | 5 | 5 | 5 | 5 | 3 | 4 | 5 | 4 | 5 | 5 | 4 | 5 | 5 | 5 | 5 |
|  | 5 | What is the difference between congenital cataract and developmental cataract? | Onset: Congenital cataracts are present at birth or shortly after, whereas developmental cataracts occur later in childhood. Causes: Congenital cataracts are typically caused by prenatal factors or genetic conditions, while developmental cataracts may result from factors occurring after birth or progress over time. Treatment: Both types of cataracts may require surgical intervention and corrective measures, but the timing and approach may vary depending on when the cataract is detected and how much it affects vision. | 4 | 4 | 5 | 5 | 4 | 4 | 5 | 4 | 4 | 5 | 4 | 4 | 5 | 5 | 5 |
|  | 6 | How serious are congenital cataracts? | The severity of congenital cataracts often depends on factors such as which eye is affected, the timing of onset, the type of cataract, the course of the disease, and when treatment is initiated. Mild clouding outside the visual axis typically has little impact on vision. However, if the cloudiness fills the central part of the pupil, it can block light from entering the eye, leading to impaired visual development. Without timely treatment, this can result in amblyopia and may lead to other complications like nystagmus or strabismus. | 5 | 5 | 5 | 5 | 4 | 4 | 5 | 3 | 4 | 5 | 4 | 4 | 5 | 5 | 5 |
|  | 7 | What are the long term effects of congenital cataracts? | The long-term effects of congenital cataracts depend on the severity of the condition and the timing of treatment. In general, mild cataracts have minimal long-term impact, with children typically maintaining good vision and being less likely to develop complications. If the cataracts are more severe but detected and treated early, the long-term effects are usually manageable, and most children can achieve normal visual development through corrective measures and visual training. However, if treatment is delayed, it can lead to significant visual impairment that may be difficult to correct, and complications such as strabismus or nystagmus may occur. | 5 | 5 | 5 | 4 | 4 | 4 | 5 | 4 | 4 | 5 | 5 | 4 | 5 | 5 | 5 |
|  | 8 | Does congenital cataract cause amblyopia? | Congenital cataracts can indeed lead to amblyopia. The clouding of the lens interferes with light entering the eye, which disrupts normal visual development in children. If both eyes are severely affected and treatment is delayed, the risk of developing amblyopia increases, and the child may experience worsening vision along with complications like strabismus or nystagmus . In cases where only one eye is affected and the other eye has a clear lens, even with timely surgery and no complications, the affected eye can still develop amblyopia if post-surgical treatment is not aggressive enough. This is because the brain tends to rely on the eye with better vision, leading to visual development problems in the weaker eye, a condition known as deprivation amblyopia. | 5 | 5 | 5 | 5 | 5 | 4 | 5 | 3 | 5 | 5 | 4 | 4 | 5 | 5 | 5 |
|  | 9 | What is the minimum age for congenital cataract surgery? | There is no strict minimum age for congenital cataract surgery, as the timing depends on the child’s overall health, the surgeon’s expertise, and the anesthesia requirements. Doctors typically aim to perform surgery during the critical period of visual development to maximize the potential for vision recovery. For one eye congenital cataracts, surgery is generally recommended within 4 to 6 weeks after birth. For bilateral cataracts, surgery can be delayed slightly. The timing is based on factors such as the type and location of the lens clouding, how much it obstructs light from entering the eye, and the development of the eye’s axial length and corneal curvature. | 5 | 5 | 5 | 5 | 5 | 5 | 5 | 4 | 5 | 5 | 5 | 5 | 5 | 5 | 5 |
|  | 10 | What happens if congenital cataracts are left untreated? | If congenital cataracts go untreated, they can lead to a range of serious consequences. First, the cloudiness of the lens obstructs light from entering the eye and focusing on the retina, which can significantly impair visual clarity and result in severe vision loss. Additionally, because the visual system develops rapidly in the first few years of life, the lack of clear visual input can cause the brain to favor the eye with better vision. This can hinder the visual development of the affected eye, leading to amblyopia. In more severe cases, untreated cataracts may also result in complications such as strabismus and nystagmus. | 5 | 5 | 5 | 5 | 5 | 4 | 5 | 3 | 5 | 5 | 5 | 4 | 5 | 5 | 5 |
|  | 11 | How successful is congenital cataract surgery? | The success rate of congenital cataract surgery depends on various factors, including the timing of the procedure, the child's age, the type and severity of the cataract, and whether the child has any other congenital abnormalities affecting the eyes. The surgeon's clinical experience and the quality of postoperative care and visual rehabilitation training also play crucial roles. The primary goal of the surgery is to restore the child's vision and prevent the development of amblyopia. However, a successful surgery does not guarantee that the child's postoperative vision and visual development will be the same as that of children without cataracts. Professional visual rehabilitation training and careful postoperative care are essential to maximize the surgical outcome. | 5 | 5 | 5 | 4 | 5 | 4 | 5 | 4 | 4 | 5 | 5 | 4 | 5 | 5 | 5 |
|  | 12 | What is the most common complication of congenital cataract surgery? | Currently, the most common complications following congenital cataract surgery are: Visual Axis Opacification (VAO): Younger patients are at a greater risk for developing VAO, which results from the regeneration of lens cortical material and leads to secondary opacification of the visual axis. Research indicates that VAO occurs in over 95% of children. Postoperative Elevated Intraocular Pressure (IOP): This complication is more common in children who have surgery without an intraocular lens, with younger patients facing a higher risk. | 5 | 5 | 5 | 3 | 4 | 5 | 5 | 4 | 4 | 5 | 4 | 4 | 5 | 4 | 4 |
|  | 13 | What is the surgery of choice for congenital cataract? | Surgeons will choose different surgical approaches based on the child's age:  For children under 2 years old: The preferred method is simple cataract extraction, followed by a secondary intraocular lens (IOL) implantation at a later stage.  For children over 2 years old: The typical procedure involves phacoemulsification cataract extraction, IOL implantation, posterior capsule removal, and anterior vitrectomy.  For children aged 5 to 8 years: The decision to perform posterior capsule removal and anterior vitrectomy will be made based on preoperative evaluations, the child's ability to cooperate during examinations, and their adherence to follow-up care. | 5 | 5 | 4 | 4 | 3 | 3 | 5 | 3 | 4 | 5 | 4 | 3 | 5 | 4 | 4 |
|  | 14 | How are congenital cataracts optically corrected? | After congenital cataract removal surgery, optical correction methods primarily include:  Glasses: They are relatively inexpensive, easy to fit, and can be changed as needed. However, children without a lens in one eye may experience discomfort due to differences in vision between the two eyes.  Contact Lenses: These include soft contact lenses and rigid gas-permeable (RGP) lenses. Contact lenses provide better visual quality and a more natural appearance, making them particularly suitable for patients with unilateral cataracts or those who cannot receive an intraocular lens. However, they tend to be more costly and require strict adherence to usage and care guidelines. | 5 | 5 | 5 | 5 | 3 | 5 | 5 | 4 | 4 | 5 | 4 | 4 | 5 | 5 | 5 |
|  | 15 | Is congenital cataract progressive? | Most congenital cataracts are present at birth and do not progress as the child grows. However, some developmental cataracts may not initially affect vision despite early lens opacification. In these cases, doctors often recommend regular follow-up and observation. As the child matures, metabolic changes may lead to increased lens clouding, resulting in what is known as adult "developmental cataracts". | 5 | 5 | 5 | 5 | 4 | 4 | 5 | 4 | 4 | 5 | 4 | 4 | 5 | 4 | 5 |
| **DD1** | 1 | Will the vision improve after congenital cataract surgery? | Many parents of children with congenital cataracts are understandably concerned about whether their child's vision will significantly improve after surgery. Today, I want to give you a detailed rundown on the surgical outcomes for congenital cataracts. As you know, the main goal of cataract surgery is to remove the cloudy lens and then implant a clear IOL. For children who aren't candidates for IOL, we correct vision with contact lenses or glasses. But here are a few key points to keep in mind for the best postoperative results.  Early Intervention: The earlier we perform the surgery, the better the outcome for vision improvement. Early intervention is crucial for the normal development of the visual pathway and reduces the risk of amblyopia. However, performing surgery too early can increase the risk of secondary glaucoma and visual axis opacification.  Postoperative Visual Rehabilitation: After cataract surgery, vision improvement in children is different from that in adults. It requires a dedicated visual rehabilitation program to effectively enhance vision. The brain needs time to adapt to the new visual input, especially in very young children, making regular rehabilitation and follow-ups essential.  Visual Prognosis Influenced by Various Factors: While vision generally improves to some extent after surgery for all children with congenital cataracts, the final visual outcome can vary due to several factors. These include the morphology of the cataract, the severity of obstruction in the visual axis, the presence of other congenital eye diseases, the timing of the surgery, potential surgical complications, the frequency and duration of postoperative rehabilitation, and whether it's unilateral or bilateral cataracts. Therefore, long-term ophthalmic follow-ups are vital for children with congenital cataracts. | 4 | 5 | 5 | 4 | 5 | 5 | 4 | 3 | 5 | 4 | 5 | 5 | 4 | 5 | 5 |
|  | 2 | If an IOL is implanted now, will it need to be replaced when the individual reaches adulthood? | In most cases, the IOLimplanted during the first cataract surgery for babies with congenital cataracts are designed to last a lifetime. They're made to be durable and serve the child's vision needs as they grow. However, there are a few considerations to keep in mind:  Changes in IOL Power: If a child's eye axis continues to grow significantly after the IOL is implanted, the power of the IOL might not be optimal when they reach adulthood. This could potentially require a replacement or adjustment of the IOL later in life, but this is not very common.  Long-term Complications with the IOL: If there's displacement, opacification, or other issues with the implanted lens, it might need to be replaced or adjusted. These situations are quite rare, but they do happen.  So, regular check-ups are essential to ensure that the IOL continues to meet the child's visual needs and to address any potential issues that may arise. | 5 | 5 | 5 | 5 | 5 | 5 | 5 | 5 | 5 | 5 | 5 | 5 | 5 | 5 | 5 |
|  | 3 | Is the congenital cataract surgery risky? | Congenital cataract surgery is generally safe and effective, but like any surgery, it comes with some risks and potential complications. Let's go over them:  1.Infection: Just like with any surgery, there's a risk of infection. But don't worry, modern minimally invasive techniques and sterile procedures, along with antibiotics used before and after surgery, have really brought that risk down.  2.Sterile Inflammation: Kids can have a more noticeable inflammatory response after cataract surgery compared to adults. If the surgery takes a long time or if there's additional surgery on the iris, the reaction can be even stronger. That's why using steroids after surgery is a must. To avoid a severe inflammatory response, it's crucial to have the surgery done at a specialized eye hospital by a doctor with lots of experience in pediatric cataract surgery.  3.Secondary Glaucoma: Sometimes after surgery, there can be a temporary increase in eye pressure. That's why we check the eye pressure at every follow-up visit, and treat it if necessary, to prevent damage to the optic nerve from high pressure.  4.IOL Malposition or Dislocation: If the cataract surgery isn't perfect, it can lead to the intraocular lens (IOL) being stuck or misaligned. Long-term misalignment can even cause the IOL to shift out of place.  Now, let's talk about the benefits and things to keep in mind:  1.Vision Improvement: The biggest benefit of congenital cataract surgery is improving vision in children during their critical period of visual development, helping to prevent or reduce amblyopia caused by visual deprivation from the cataract.  2.Early Intervention: Getting the surgery early can help prevent or minimize the impact of the cataract on the development of the visual cortex, reducing the risk of complications like amblyopia.  3.Long-term Monitoring: Regular follow-ups are essential to ensure long-term vision improvement.  In summary, congenital cataract surgery is a proven and effective procedure with a high success rate. While the risks can't be completely avoided like they can with adult cataract surgery, parents of children with congenital cataracts should discuss the potential risks and benefits with their ophthalmologist before the surgery. This will help make an informed decision and prepare for the postoperative recovery process. | 5 | 5 | 5 | 4 | 5 | 5 | 4 | 4 | 5 | 5 | 5 | 5 | 5 | 5 | 5 |
|  | 4 | Do we have to proceed with the congenital cataract surgery now, or can we wait? | Parents of babies with congenital cataracts have to decide whether to go ahead with surgery right away or wait, and there are a few key things to think about: (1) The severity of the cataracts: The need for surgery is based on how bad the cataracts are and how much they affect vision. If it's a total white cataract or a posterior subcapsular cataract in the visual axis, not treating it in time could lead to amblyopia or low vision. If the cataracts aren't significantly harming vision or causing other issues, I'd suggest regular check-ups and elective surgery if needed. (2) Visual development: Early intervention is crucial for proper visual development. The visual pathways in a young child's brain are still developing, and timely surgery can reduce the risk of deprivation amblyopia and abnormal visual development caused by the cataracts. (3) Preventing complications: Delaying treatment for cataracts can lead to other complications, such as strabismus, nystagmus, and poor stereopsis, which are visual dysfunctions. (4) Age and overall health: The child's overall health and readiness for general anesthesia are important. If the baby is too young or has other health concerns, we might recommend waiting. In the end, I advise parents to carefully weigh the pros and cons when making the decision for surgery. If you decide to wait, regular eye exams to monitor the cataracts and ensure they're not negatively affecting your child's vision are essential. | 4 | 5 | 5 | 4 | 5 | 5 | 4 | 5 | 5 | 4 | 5 | 5 | 4 | 5 | 5 |
|  | 5 | Will congenital cataract redevelop after the surgery? | If congenital cataract surgery in children and infants doesn't involve removing the posterior capsule, there's a 100% chance of developing posterior capsule opacification (PCO), also known as Visual Axis Opacification (VOA). So, how can we prevent or avoid the occurrence of PCO or VOA? Here are some important considerations to keep in mind:  (1) The younger the child, the more likely they are to develop VOA. Surgeons should aim to remove as much peripheral cortex as possible during surgery for children under six and ensure that the posterior capsule and anterior vitreous are removed with a sufficient diameter.  (2) For children over six years old with congenital cataracts, the surgery can involve a simple posterior capsule tear during the procedure or, in the first few months after cataract removal when the child is more cooperative, a relatively straightforward outpatient procedure known as YAG laser posterior capsulotomy.  (3)Regular follow-ups: Even with a successful cataract surgery where the posterior capsule and anterior vitreous are adequately removed, some children may still develop PCO or VOA. It's essential to have regular eye exams post-surgery, and if VOA or PCO occurs, a secondary posterior capsulotomy may be necessary. | 5 | 5 | 5 | 5 | 5 | 5 | 5 | 3 | 5 | 5 | 4 | 5 | 5 | 5 | 5 |
|  | 6 | Can we continue with amblyopia training and delay the congenital cataract surgery for a few years? | Although amblyopia training is a crucial part of managing vision development, if cataracts have already caused opacification in the visual axis, leading to deprivation amblyopia, regular amblyopia training cannot replace the need for congenital cataract surgery. Why do we say that? Let's analyze the importance of early surgery for congenital cataracts:    (1) Visual development: For infants and young children, the main goal of cataract surgery is to prevent or reduce vision loss. Cloudy lenses can interfere with the development of the brain's visual pathways. Removing the cataracts early on helps ensure stable vision improvement and reduces the risk of severe amblyopia.    (2) Preventing amblyopia: Congenital cataracts, especially in one eye, can lead to amblyopia if left untreated. Amblyopia treatments, such as glasses, patching the sound eye, and other therapies, are more effective the earlier they are started. Training after cataract removal yields much better results than before removal.    (3) Risks of delayed surgery: Progressive congenital cataracts can lead to ongoing vision loss and increase the difficulty of amblyopia training if surgery is delayed. The longer the cataract affects vision, the harder it is to improve it, even with ongoing amblyopia training.    (4) Complications: Untreated cataracts can lead to other complications, such as strabismus, nystagmus, and poor stereopsis, which are visual dysfunctions that complicate later treatment and management.    It's clear that early surgery is the best way to prevent complications from congenital cataracts and provides the best foundation for vision recovery. | 5 | 5 | 5 | 4 | 5 | 5 | 5 | 4 | 5 | 5 | 5 | 5 | 5 | 5 | 5 |
|  | 7 | Our eldest child has bilateral congenital cataracts, and we are considering having a second child. What is the likelihood of the second child developing cataracts? | The likelihood of a child having congenital cataracts can be influenced by a variety of factors, including genetic and environmental factors.    (1) Let's talk about genetic factors first: If your first child has congenital cataracts due to genetic reasons, it might increase the risk for your second child to develop cataracts as well. Some hereditary conditions related to congenital cataracts can be passed down from parents to their children. In such cases, it's recommended to undergo genetic testing to more accurately assess the risk for your second child to have cataracts.    (2) Environmental and other factors: Non-genetic causes: Congenital cataracts can also be caused by non-genetic factors, such as prenatal infections (like rubella) or exposure to certain substances during pregnancy. If your eldest child's cataracts were due to these factors, the risk for your second child might be reduced. Maintaining good prenatal care and avoiding known risks is important. Make sure you receive regular prenatal care to monitor the health of your pregnancy and address any potential cataract risks.    Overall, if congenital cataracts are caused by genetic factors, then each pregnancy might carry a higher risk. Getting timely prenatal genetic testing, consulting with a genetic specialist, and obtaining the most accurate information can help parents make informed decisions. | 5 | 5 | 5 | 5 | 5 | 5 | 5 | 4 | 5 | 5 | 4 | 5 | 5 | 5 | 5 |
|  | 8 | After congenital cataract surgery, is it better to wear rigid gas-permeable (RGP) lenses or regular spectacles? | After a congenital cataract extraction, choosing the right type of eyewear for an aphakic eye can be a challenge. The decision between rigid gas-permeable (RGP) contact lenses and regular spectacles depends on several factors, including the patient's specific needs, the visual quality of an intraocular lens (IOL) if used, and the comfort of the baby's eyes. Let's compare the two to help you understand:  Advantages of RGP lenses: (1) They can provide undistorted good vision, especially if there is an irregular cornea or high hyperopia that cannot be fully corrected by spectacles. (2) Stability: RGP offers a stable and precise optical correction, even for irregular astigmatism.  Disadvantages: (1) Comfort: They may not be as comfortable as soft contact lenses or glasses, especially for babies who are not used to wearing them. (2) Adaptation: It might take some time for the child to get used to wearing RGP lenses.  Advantages of regular spectacles: (1) Ease of use: Glasses are easy to use, do not require direct contact with the eyes, and are universally applicable. (2) Comfort: Some babies may find glasses more comfortable than contact lenses. (3) Less maintenance: They are easier to clean and maintain than contact lenses.  Disadvantages: (1) Limited vision correction: Spectacles cannot correct high hyperopia in aphakic eyes as effectively as contact lenses, especially when combined with irregular astigmatism. (2) Peripheral vision: Glasses do not provide as wide a peripheral field of view as RGP lenses.  In summary, RGP lenses and regular spectacles each have their pros and cons. An ophthalmologist or optometrist can provide personalized advice based on the results of the cataract surgery and the child's visual requirements. If considering the child's comfort and a simple, harmless lifestyle, spectacles might be preferred over RGP. Regular follow-ups with an ophthalmologist or optometrist are crucial to ensure that the chosen correction method provides the best vision and comfort. | 5 | 5 | 5 | 5 | 5 | 4 | 5 | 3 | 5 | 5 | 4 | 4 | 5 | 5 | 5 |
|  | 9 | The child currently has nystagmus. Will the nystagmus resolve after congenital cataract cataract surgery? | Nystagmus refers to an involuntary rapid eye movement, and its presence after congenital cataract surgery may improve, remain the same, or even worsen. The impact of surgery on nystagmus depends on various factors:  (1) Improving visual clarity: If the cataract severely impedes vision, removing it can enhance visual clarity. Enhanced visual input may reduce nystagmus to some extent, especially if the primary cause of the nystagmus is poor visual input.  (2) Reducing visual disability: Improved vision can help stabilize the visual system, which may lead to a decrease in the intensity of nystagmus.  (3) Reasons for unchanged nystagmus: If the nystagmus is due to underlying neurological or structural issues unrelated to the cataract, the surgery may not affect it.  Close monitoring of the child's vision and nystagmus after cataract surgery is essential. If nystagmus persists, surgical treatment may be considered based on the specific circumstances. It requires a collaborative effort from pediatric ophthalmologists, neurologists, and strabismus specialists to address nystagmus.  In summary, while congenital cataract surgery can improve vision and potentially have a positive effect on nystagmus, it does not guarantee the complete resolution of nystagmus. Depending on the specific circumstances of the child with congenital cataracts and their response to surgery, other interventions may be necessary. | 5 | 5 | 5 | 5 | 5 | 5 | 4 | 3 | 5 | 5 | 4 | 5 | 5 | 5 | 5 |
|  | 10 | We have unilateral congenital cataract in our child. It's said that the surgical outcomes for unilateral cases are not as good. Is this true? | The surgical outcomes for congenital cataracts in one eye are indeed different from those in both eyes. The reason is that a cataract in one eye deprives it of clear vision for a certain period after birth, affecting the development of the visual nerves on that side. Meanwhile, the healthy eye on the opposite side develops normally, leading the child to rely primarily on the healthy eye for visual tasks and the transmission of visual information before cataract surgery and amblyopia training. As a result, the eye with the cataract is highly likely to develop amblyopia. Successful treatment requires not only the removal of the cataract but also the use of forced visual training by patching the healthy eye. This encourages the brain and visual system to adapt well, especially if the surgery is performed early. This adaptation can help achieve near-normal vision, although some degree of visual difference between the two eyes may persist.  Therefore, with timely cataract surgery and continuous, proper patching for amblyopia training, many children with unilateral congenital cataracts can achieve good vision. However, the final visual outcomes may vary depending on factors such as the age at the time of surgery, the presence of amblyopia, and the effectiveness of postoperative treatment. Unilateral congenital cataracts present unique challenges in terms of visual development and amblyopia. The key to success lies in early diagnosis and treatment, followed by persistent postoperative patching for amblyopia training. | 5 | 5 | 5 | 5 | 4 | 5 | 4 | 4 | 5 | 5 | 4 | 5 | 5 | 5 | 5 |
|  | 11 | After congenital cataract surgery in a child, is it necessary to regularly monitor intraocular pressure and axial length? | Regular postoperative checks of intraocular pressure (IOP) and eye axial length in children with congenital cataracts are crucial.  (1) Regular IOP monitoring is vital due to the 3-5% risk of secondary glaucoma after surgery. Factors like inflammation, anatomical changes, and corticosteroid use can play a role. These checks aid in early glaucoma detection and management.  (2) Axial length monitoring is important as it grows with the child, sometimes faster after cataract surgery. It helps track refractive error changes and adjust correction strategies like lens replacements or glasses. Follow-ups at 1 week, 1 month, 3 months, 6 months, and yearly are typical, aiding in managing long-term post-surgical complications and ensuring proper vision correction. | 5 | 5 | 5 | 5 | 4 | 5 | 5 | 5 | 5 | 5 | 5 | 5 | 5 | 5 | 5 |
|  | 12 | How long after congenital cataract surgery should the child avoid physical education classes? | The answer to this question depends on several factors, including the overall eye health of the child and the complexity of the cataract surgery. As a general guideline, it's recommended that children with congenital cataracts avoid strenuous exercise and physical education classes for at least 4 to 6 weeks after cataract surgery. This period allows the eye to heal properly and minimizes the risk of surgical complications, such as displacement of the intraocular lens, infection within the eye, dehiscence of the incision, or injury to the eye. Additionally, activities involving weightlifting, running, or jumping should be avoided.  Consider engaging in some gentle activities to gradually regain strength. Regular follow-ups with an eye doctor are essential to assess the progress of the surgical wound healing. If the child participates in sports or activities that could potentially cause eye injury, it is advisable to take extra protective measures, such as wearing safety goggles or eye shields. | 4 | 5 | 5 | 4 | 5 | 5 | 5 | 4 | 5 | 5 | 4 | 5 | 5 | 4 | 5 |
|  | 13 | Can a child have a multifocal intraocular lens implanted during congenital cataract surgery, or is a monofocal lens the only option? | Selecting an IOL for kids' cataract surgery often favors single-focus IOLs, ideal for young children. These IOLs are simple, safe, and maintain post-op contrast sensitivity, fitting most children with cataracts and amblyopia. Their predictability aids in post-op refractive adjustments, vital for children's changing vision.  In young kids' visual development, single-focus IOLs set for distance let them use near-vision correction as needed, keeping some hyperopia. This is manageable as their eye axis grows. Multifocal IOLs, offering various focal points, might reduce contrast and cause visual issues, less suitable for post-cataract amblyopia in kids. They fit adults better with stable visual needs.  So, single-focus IOLs are preferred for pediatric cataract surgery. | 5 | 5 | 5 | 5 | 4 | 4 | 5 | 4 | 5 | 5 | 5 | 4 | 5 | 5 | 5 |
|  | 14 | The severity of congenital cataracts in both of children's eyes is not consistent, should we do cataract surgery in both eyes, or do the severe eye first? | When addressing congenital cataracts of varying severities in both eyes, the timing of surgery and the eye to operate on first typically require careful consideration of the following factors:  Prioritize the eye with the more severe cataract. If one eye has a significantly opaque lens causing greater visual impairment, it often requires surgery first to prevent further damage to visual development. Early intervention in the more severely affected eye can help reduce the risk of developing amblyopia.  After addressing the more severely affected cataract, the next step is to assess how to manage the less affected eye. The timing of the second eye's surgery depends on factors such as the state of visual development and whether the less affected eye is beginning to exhibit more visual issues. The child's age and their stage of visual development are crucial. Early surgery is vital for promoting normal visual development. However, the risks associated with pediatric cataract surgery are higher than in adults, so managing these risks is also essential. Choosing an optimal surgery time for the less affected eye with cataract is important. Whether to perform surgery on both eyes simultaneously or in stages, a personalized plan suitable for pediatric bilateral cataracts should be determined.  In conclusion: In most cases, the more severely affected eye is prioritized for surgery to address significant visual impairment and reduce the risk of amblyopia. Then, close monitoring and appropriate management measures are taken for the less affected cataract eye, which helps to manage risks and optimize visual outcomes. | 5 | 5 | 5 | 5 | 5 | 5 | 5 | 3 | 5 | 5 | 4 | 5 | 5 | 5 | 5 |
|  | 15 | The congenital cataract surgery has been completed, and now amblyopia training is required. However, all amblyopia training programs increase the duration and intensity of eye use, which could lead to a worsening of myopia. Is this a concern? | Amblyopia therapy is key after congenital cataract surgery. It's a common worry that this therapy could increase myopia, but it's manageable. The goal is to boost vision in the weaker eye and support visual growth. Activities focus on visual engagement and eye coordination.    Increased eye use might affect refractive errors, but the benefits of treatment are significant. Eye care professionals track refractive changes. If myopia progresses after surgery, strategies like lens adjustments, special lenses, atropine eye drops, or lifestyle changes can help.    Treatment should be personalized. Training intensity varies with the severity of amblyopia to avoid eye strain.    In summary, while amblyopia training could potentially lead to myopia, this risk is controlled with monitoring and individualized plans. The benefits of improved vision and development from amblyopia training usually outweigh the myopia risks. | 5 | 5 | 5 | 4 | 4 | 5 | 5 | 5 | 5 | 5 | 5 | 5 | 5 | 5 | 5 |
| **DG2** | 1 | What are congenital cataracts? | Congenital cataracts are a unique kind we're talking about. They pop up right after a baby's born or within their first year. It's possible that they're linked to genetic factors, infections that happen in the womb, or even not getting enough nutrients while the baby's growing inside the mom. | 4 | 4 | 4 | 3 | 3 | 4 | 4 | 4 | 4 | 4 | 4 | 4 | 4 | 4 | 4 |
|  | 2 | What is the most common type of congenital cataract? | When we're talking about the different looks of congenital cataracts, the most common one we see is called a lamellar cataract. It's the most, making up about 34% of the cases. | 5 | 4 | 3 | 5 | 3 | 3 | 5 | 4 | 3 | 5 | 3 | 3 | 5 | 5 | 3 |
|  | 3 | Why was I born with cataracts? | This could be due to a few things happening during pregnancy. It might be about our genes, little changes in them we call mutations. Or maybe infections that happen in the womb, like from viruses named rubella or cytomegalovirus. Also, not getting the right nutrients can play a part too. | 5 | 5 | 4 | 5 | 4 | 4 | 5 | 4 | 4 | 5 | 4 | 4 | 5 | 5 | 4 |
|  | 4 | What percent of people have congenital cataracts? | The incidence rate of congenital cataracts is estimated to be between 12 to 136 per 100,000 newborns. | 5 | 4 | 4 | 4 | 3 | 4 | 5 | 5 | 5 | 5 | 4 | 4 | 5 | 4 | 4 |
|  | 5 | What is the difference between congenital cataract and developmental cataract? | (a) Different reasons: Developmental cataracts often come from genes, the world around us, or not eating enough good food messing with how the lens in our eye works. Congenital cataracts usually happen because something went wrong as we were growing in the womb, mom getting sick, or how our body processes things. (b) Different signs: People with developmental cataracts might see less clear, things might look blurry, or their eyes might not look straight ahead. Those with congenital cataracts might see very poorly, have shaky eyes, or their eyes might not line up right. (c) Different outcomes: Developmental cataracts usually don't mess with our sight too much, and if we get help soon, we can see fine later on. But congenital cataracts, if we don't get help quick, can make it hard to see and sometimes even lead to lazy eyes or not seeing at all. | 3 | 3 | 4 | 4 | 4 | 4 | 4 | 5 | 4 | 4 | 4 | 4 | 3 | 4 | 4 |
|  | 6 | How serious are congenital cataracts? | If not treated promptly, congenital cataracts can affect vision and may even lead to amblyopia and blindness in severe cases. | 4 | 5 | 4 | 3 | 2 | 3 | 4 | 5 | 3 | 4 | 2 | 3 | 3 | 3 | 3 |
|  | 7 | What are the long term effects of congenital cataracts? | The long-term impact of congenital cataracts is mainly about the drop in vision or the lack of improvement in sight, which is all about catching it early, getting it treated right away, and following up with visual training. If we can get the surgery done in time, patients need to keep an eye on their vision, the growth of their eye axis, and the development of their visual functions over the long haul, and they might need to do some amblyopia training too. But if we don't catch it and treat it early, it can lead to a decline in vision, a halt in eye growth, and in the long run, it might cause amblyopia or even blindness. | 4 | 4 | 4 | 4 | 3 | 4 | 5 | 4 | 4 | 5 | 3 | 4 | 5 | 4 | 4 |
|  | 8 | Does congenital cataract cause amblyopia? | Congenital cataracts can easily lead to amblyopia, but if we catch it early and get the surgery done, along with follow-up amblyopia training, some kids can regain normal vision. But if it's not treated promptly and correctly, there's a good chance it could develop into amblyopia. | 5 | 5 | 5 | 5 | 4 | 4 | 5 | 4 | 4 | 5 | 4 | 4 | 5 | 4 | 4 |
|  | 9 | What is the minimum age for congenital cataract surgery? | The timing for surgery depends on how severe the cataracts are and the overall health of the child. For unilateral, dense cataracts, we can operate around 4 to 6 weeks after birth. For bilateral, dense cataracts, we might wait until 6 to 8 weeks after birth. But for the less severe cases, we can adjust the surgery time according to the child's specific situation and the degree of the cataracts. | 5 | 5 | 5 | 5 | 4 | 4 | 5 | 5 | 5 | 5 | 5 | 5 | 5 | 5 | 5 |
|  | 10 | What happens if congenital cataracts are left untreated? | If not treated promptly, it will lead to a decrease in vision, cessation of eyeball development, and in the long run, may result in amblyopia or even blindness. | 5 | 4 | 4 | 4 | 4 | 4 | 5 | 4 | 4 | 5 | 3 | 4 | 5 | 4 | 4 |
|  | 11 | How successful is congenital cataract surgery? | The success of cataract surgery really hinges on a few key things: the surgeon's experience, the right timing for the operation, how bad the cataracts are, the kid's age, and their overall health. Fortunately, we've got a high success rate with congenital cataract surgeries these days. We make sure to do a complete work-up before the surgery to give it the best shot at going well. | 5 | 5 | 4 | 5 | 3 | 3 | 5 | 4 | 4 | 5 | 5 | 4 | 5 | 4 | 3 |
|  | 12 | What is the most common complication of congenital cataract surgery? | In congenital cataract surgery, the most common hitches we see are clouding up of the visual axis, which includes what we call posterior capsule opacification (PCO) and secondary membrane formation. It's pretty much a given with kids born with cataracts, especially the little ones. When we operate and leave the back part of the lens capsule whole, this can happen. To handle it, we often wait until the kid is at a certain age, then we might do a laser treatment or sometimes we've got to go in there and do a second surgery. | 4 | 4 | 4 | 4 | 4 | 4 | 5 | 3 | 3 | 4 | 3 | 3 | 4 | 4 | 4 |
|  | 13 | What is the surgery of choice for congenital cataract? | Depending on the age of the child, we tailor the surgical approach. For kids under 2, we go with a straightforward cataract removal, and then later on, we do a secondary IOL implantation. For those older than 2, the plan is to do phacoemulsification to remove the cataract, pop in an intraocular lens, and also take out the posterior capsule and the anterior vitreous. For kids aged 5 to 8, we look at how well they can cooperate during the pre-op exams and how likely they are to stick with follow-up care, and based on that, we make a call on whether to do a posterior capsulotomy and anterior vitrectomy. | 5 | 4 | 3 | 4 | 3 | 3 | 5 | 3 | 3 | 5 | 4 | 3 | 5 | 4 | 3 |
|  | 14 | How are congenital cataracts optically corrected? | RGP lenses and glasses can both be used. But because RGP lenses do a great job of minimizing optical aberrations and distortions, which means better image quality, and because they promote the development of single binocular vision and fusion functions, and can improve visual quality for kids with nystagmus, RGP is really the preferred method for correcting vision after congenital cataract surgery. | 5 | 4 | 4 | 4 | 3 | 4 | 5 | 4 | 4 | 4 | 3 | 3 | 4 | 4 | 3 |
|  | 15 | Is congenital cataract progressive? | Usually, congenital cataracts are there right from birth and don't get worse as a child grows. But, if treatment is delayed, the vision problems from congenital cataracts can get more serious over time, and they might cause other issues like strabismus. | 5 | 5 | 4 | 4 | 4 | 3 | 5 | 5 | 4 | 5 | 5 | 4 | 5 | 5 | 4 |
| **DD2** | 1 | Will the vision improve after congenital cataract surgery? | After the surgery, most kids, with the help of some vision exercises or wearing glasses right on time, can see a lot better. | 4 | 5 | 5 | 4 | 2 | 4 | 5 | 4 | 4 | 4 | 3 | 4 | 4 | 4 | 4 |
|  | 2 | If an IOL is implanted now, will it need to be replaced when the individual reaches adulthood? | Right now, the majority of kids don't need a lens implant again. Before we go for an IOL, we take into account factors like potential impact on vision, eye axis development, and refractive shifts to choose the right one. In some cases, if there's a significant mismatch in refractive power that's hard to correct, a replacement might be necessary. | 4 | 3 | 5 | 4 | 3 | 4 | 4 | 5 | 4 | 4 | 3 | 4 | 4 | 5 | 5 |
|  | 3 | Is the congenital cataract surgery risky? | The risks of surgery for congenital cataracts really hinge on the severity of the child's condition, their overall health, how they handle anesthesia, the skill level of the surgeon, and the timing of the surgery. | 4 | 4 | 5 | 4 | 3 | 4 | 5 | 5 | 4 | 4 | 5 | 5 | 4 | 5 | 5 |
|  | 4 | Do we have to proceed with the congenital cataract surgery now, or can we wait? | The timing for surgery really depends on how severe the cataracts are and the child's overall health. For single eye, dense cataracts, we can operate around 4 to 6 weeks after birth; for both eyes with dense cataracts, we typically do the surgery between 6 to 8 weeks after birth. With less severe cataracts, we can adjust the surgery time based on the child's specific condition and how serious it is. Plus, whether we go for surgery or not, we recommend that patients get prompt visual training, which can help improve their best possible vision. On the flip side, if we delay surgery, the affected eye can develop amblyopia due to deprivation of visual input, and the later we treat it, the harder it is to boost the vision. | 5 | 5 | 5 | 4 | 4 | 4 | 4 | 4 | 4 | 5 | 5 | 5 | 5 | 5 | 4 |
|  | 5 | Will congenital cataract redevelop after the surgery? | Some kids have leftover lens epithelial cells on the capsule that can grow and move, causing a secondary cataract and leading to a drop in vision. For cooperative kids, we can do a YAG laser capsulotomy right in the clinic; for those who have a hard time cooperating, we might opt for a surgical removal of the posterior capsule along with anterior vitrectomy. | 4 | 5 | 5 | 4 | 5 | 5 | 4 | 4 | 4 | 4 | 5 | 5 | 4 | 5 | 5 |
|  | 6 | Can we continue with amblyopia training and delay the congenital cataract surgery for a few years? | The main reason children with congenital cataract get amblyopia is because their eyes don't get the right kind of stimulation they need to grow properly. The cataract blocks the view, so the eye doesn't develop like it should. To really give these kids a chance to see better, we've got to fix this lack of stimulation at the root. | 3 | 3 | 4 | 3 | 2 | 4 | 4 | 3 | 3 | 3 | 3 | 4 | 3 | 3 | 4 |
|  | 7 | Our eldest child has bilateral congenital cataracts, and we are considering having a second child. What is the likelihood of the second child developing cataracts? | Congenital cataracts stem from a mix of genetic and environmental factors. Studies suggest that about 10% to 25% of congenital cataracts are inherited. If a couple's first child has congenital cataracts due to genetic causes, there's a higher likelihood that a second child could develop them too. Also, if the parents themselves have congenital cataracts, their offspring are at an increased risk of having it. | 4 | 4 | 4 | 4 | 4 | 3 | 4 | 4 | 4 | 4 | 4 | 4 | 4 | 4 | 4 |
|  | 8 | After congenital cataract surgery, is it better to wear rigid gas-permeable (RGP) lenses or regular spectacles? | So, both have their pros and cons. RGP lenses do a great job of minimizing optical aberrations and distortions, giving us crisp image quality; they also promote the development of binocular vision and fusion functions; and they can improve visual quality for kids with nystagmus. The downside is they can be pricey and a bit of a hassle to handle. If not worn or cared for properly, there's a risk of corneal or conjunctival damage or infection. On the other hand, glasses are easy to wear and cost-effective, and they're hygienic. But they've got their drawbacks too, like peripheral image distortion, a reduction in retinal image size, and a narrower peripheral field of view. For kids without an intraocular lens: if it's a single eye, we'd go for contact lenses as the first choice; if it's both eyes, we can consider either contacts or glasses. For those with an intraocular lens, glasses can do the trick. And if glasses aren't suitable, contact lenses might be the way to go. | 4 | 5 | 5 | 4 | 5 | 5 | 4 | 5 | 5 | 4 | 5 | 5 | 4 | 5 | 5 |
|  | 9 | The child currently has nystagmus. Will the nystagmus resolve after congenital cataract cataract surgery? | Nystagmus can have a variety of causes. The kind that's due to cataracts messing with the development of fixation can improve to some extent after cataract surgery when visual function gets better. Some kids might even see it disappear completely. | 4 | 3 | 4 | 4 | 3 | 4 | 5 | 4 | 4 | 4 | 4 | 4 | 4 | 4 | 4 |
|  | 10 | We have unilateral congenital cataract in our child. It's said that the surgical outcomes for unilateral cases are not as good. Is this true? | Unilateral congenital cataracts tend to have a higher risk of amblyopia post-surgery, and the improvement in vision from treatment isn't always as marked as with bilateral cases. However, identifying it early, getting it taken care of with surgery, and sticking with amblyopia training can really optimize the surgical results for these kids. | 5 | 4 | 5 | 4 | 2 | 4 | 5 | 4 | 4 | 5 | 4 | 4 | 5 | 3 | 4 |
|  | 11 | After congenital cataract surgery in a child, is it necessary to regularly monitor intraocular pressure and axial length? | After surgery, it's important to regularly measure the axial length and intraocular pressure. On one hand, there's a higher chance of increased eye pressure after congenital cataract surgery, and keeping an eye on it helps prevent post-op complications. On the other hand, the growth of the axial length is a pretty objective measure of how the eye is developing, and it needs to be monitored regularly to keep track of ocular growth. This way, we can make timely adjustments to the kid's refractive correction plan. | 5 | 5 | 5 | 4 | 5 | 5 | 5 | 5 | 5 | 5 | 5 | 5 | 5 | 5 | 5 |
|  | 12 | How long after congenital cataract surgery should the child avoid physical education classes? | About three monthes. | 3 | 3 | 3 | 3 | 2 | 3 | 5 | 5 | 4 | 3 | 3 | 3 | 3 | 2 | 4 |
|  | 13 | Can a child have a multifocal intraocular lens implanted during congenital cataract surgery, or is a monofocal lens the only option? | 1.Most kids with congenital cataracts have amblyopia, so they're not really good candidates for multifocal IOLs; 2.The axial length of a child's eye can still change before they turn 18, and post-surgery refractive changes can significantly reduce the effectiveness of multifocal IOLs, and might even increase the risk of amblyopia. So, we generally don't recommend implanting multifocal IOLs in children's eyes. | 5 | 4 | 5 | 4 | 4 | 4 | 5 | 5 | 4 | 5 | 4 | 5 | 5 | 5 | 5 |
|  | 14 | The severity of congenital cataracts in both of children's eyes is not consistent, should we do cataract surgery in both eyes, or do the severe eye first? | Our surgical planning takes a variety of factors into account. If the child is young and has bilateral cataracts that are relatively severe, we'd recommend simultaneous cataract removal in both eyes, followed by secondary IOL implantation at a later stage. If the child is older and the cataracts vary in severity between the eyes, we'd opt to operate on the more severely affected eye first. | 5 | 5 | 5 | 4 | 5 | 5 | 4 | 4 | 5 | 4 | 5 | 5 | 4 | 5 | 5 |
|  | 15 | The congenital cataract surgery has been completed, and now amblyopia training is required. However, all amblyopia training programs increase the duration and intensity of eye use, which could lead to a worsening of myopia. Is this a concern? | Theoretically, vision training, because it enhances visual stimulation, could lead to an increase in myopia in children. But without amblyopia training, the child's corrected vision won't improve, which could negatively impact their long-term visual development. It's recommended to pay attention to eye posture during amblyopia training, maintain a good amount of outdoor activity, and regularly check the refractive status to try to prevent the progression of myopia. | 5 | 5 | 5 | 5 | 4 | 5 | 5 | 5 | 5 | 5 | 5 | 5 | 5 | 5 | 5 |
| **RE-CG** | 1 | What are congenital cataracts? | Congenital cataracts happen when a baby is born with a cloudy lens in the eye. This cloudiness can make it hard to see clearly. The lens is the part of the eye that helps focus light so we can see well. When the lens is cloudy, light can’t pass through easily, which makes vision blurry. | 3 | 3 | 5 | 3 | 3 | 5 | 4 | 5 | 5 | 4 | 4 | 5 | 3 | 4 | 5 |
|  | 2 | What is the most common type of congenital cataract? | The most common type of congenital cataract is called a nuclear cataract. This type affects the middle part of the lens in the eye. Nuclear cataracts make the center of the lens cloudy, which can cause blurry vision.  Key Points About Nuclear Cataracts: Appearance: Nuclear cataracts look like a cloudy or white spot in the center of the lens. In babies, you might see a white or gray color in their pupil.  Causes: Nuclear cataracts can happen for a few reasons:  Genetics: They can be passed down in families. Inherited Conditions: Sometimes, they are linked to other health issues. Spontaneous: They can also happen without any other problems. Detection: Doctors usually find nuclear cataracts during regular eye exams for babies and young children. Parents might notice their child has trouble seeing. Finding cataracts early is important so they can be treated.  Treatment: The main treatment is surgery to remove the cloudy lens. After surgery, children might need glasses or other tools to help them see better.  Other Types of Cataracts: There are other types of congenital cataracts:  Cortical Cataracts: These affect the outer part of the lens. Posterior Subcapsular Cataracts: These affect the back part of the lens. The type of cataract and how it affects vision helps doctors decide the best way to treat it. | 5 | 2 | 5 | 4 | 4 | 5 | 4 | 4 | 5 | 5 | 3 | 5 | 5 | 3 | 5 |
|  | 3 | Why was I born with cataracts? | Congenital cataracts are cataracts that babies are born with or that develop soon after birth. They can be caused by different things. Sometimes, they happen because of genetic changes or inherited conditions. Other times, they may be linked to infections or problems during pregnancy, like rubella or other illnesses. In some cases, we might not know the exact cause.  Congenital cataracts can be mild or severe, and they can affect how well a person can see. It’s important to have regular eye check-ups to watch for any problems. If you have questions or concerns about your situation, it’s best to talk to your eye doctor. They can give you helpful advice based on your specific case. | 4 | 4 | 5 | 4 | 3 | 5 | 4 | 5 | 5 | 5 | 5 | 5 | 5 | 5 | 5 |
|  | 4 | What percent of people have congenital cataracts? | Congenital cataracts are quite rare. They happen in about 1 to 6 out of every 10,000 babies born. This means that only about 0.01% to 0.06% of babies are born with cataracts.  The number of cases can be a little different depending on where people live and how good their healthcare is. Overall, congenital cataracts are not very common compared to other eye problems. Finding and treating them early, usually when babies are very young, is really important. This helps to keep their vision and prevents long-term vision problems. | 4 | 4 | 5 | 4 | 3 | 5 | 5 | 5 | 5 | 5 | 5 | 5 | 5 | 5 | 5 |
|  | 5 | What is the difference between congenital cataract and developmental cataract? | There are two main types of cataracts in kids: congenital and developmental. They happen at different times and have different causes.  Congenital Cataracts: What They Are: These cataracts are present when a baby is born or appear soon after birth. Causes: They can happen because of genes, infections during pregnancy (like rubella), or other problems before the baby is born. Features: Congenital cataracts can be in one or both eyes and may look different in size or how cloudy they are. Doctors usually find them during a baby’s first check-ups. Treatment: Surgery is often needed to remove the cataract, and glasses or other tools may help the baby see better after surgery. Developmental Cataracts: What They Are: These cataracts show up later, usually after the first few months of life. Causes: They can be caused by injury, health problems, or sometimes we don’t know the cause. They may also be linked to other eye issues. Features: Developmental cataracts appear as a child grows and can get worse over time. They can affect one or both eyes. Treatment: Doctors will treat based on how bad the cataracts are. Treatment may include regular check-ups, glasses, or surgery if needed. Main Differences: Timing: Congenital cataracts are there from birth. Developmental cataracts show up later. Causes: Congenital cataracts happen because of problems before birth, while developmental cataracts happen after birth. Treatment: Both may need surgery or glasses, but the timing and care will depend on how they affect vision. This helps doctors give the right treatment so children can have the best vision possible. | 5 | 5 | 4 | 5 | 4 | 4 | 5 | 5 | 4 | 5 | 5 | 4 | 5 | 5 | 4 |
|  | 6 | How serious are congenital cataracts？ | Congenital cataracts can be a big problem, especially if they aren't treated early. How serious they are depends on how big the cataract is and if it’s in one or both eyes. Here’s why they are serious:  How They Affect Vision Vision Problems: Cataracts block light from reaching the back of the eye, making it hard to see clearly or causing blindness. Slowed Development: Babies and young kids need clear vision to grow. If cataracts aren’t treated, it can slow down vision development and cause problems like lazy eye (where the brain ignores the weak eye). Other Problems Lazy Eye (Amblyopia): If the cataract makes it hard to see, the brain may not learn to use that eye properly. Crossed Eyes (Strabismus): Poor vision can make the eyes not line up, leading to more vision problems that may need extra treatment. Treatment Early Treatment: Doctors need to find and treat cataracts as soon as possible. Surgery is usually needed to take out the cataract so the child can see better. Early surgery helps the eyes develop properly. Ongoing Care: After surgery, kids may need glasses or other tools to help them see. Regular eye check-ups are important to make sure their vision gets better. Long-term Results With Treatment: Many kids can see well if they get treatment early. Without Treatment: If cataracts aren’t treated, they can cause permanent vision problems and slow learning. In Short Congenital cataracts can cause serious vision problems and slow a child’s development. Early treatment is very important to help the child see better. | 5 | 5 | 5 | 5 | 5 | 5 | 5 | 5 | 5 | 5 | 5 | 5 | 5 | 5 | 5 |
|  | 7 | What are the long term effects of congenital cataracts? | Congenital cataracts can affect each child differently. How it affects them depends on when it is found, how serious it is, and how well it is treated. Here are some possible long-term effects:  Vision Problems Blurry Vision: Even after treatment, some children may still not see clearly, especially if the cataract was big or in both eyes. Lazy Eye: If the cataract isn’t fixed soon, the brain might stop using the weak eye, leading to vision problems. Eye Function Eyes Not Working Together: The eyes might not work well together, which makes it hard to see how far away things are. Some kids may end up with crossed eyes and need more treatment. Trouble with Some Activities: Even after surgery, some children might find it hard to do things that need good vision, like reading or playing sports. School and Social Life Learning Difficulties: Vision problems can make learning harder. Kids might need extra help at school. Social and Emotional Effects: Problems with vision can affect how children feel about themselves and how they get along with others. They might need support to feel confident. Ongoing Care Glasses: Many kids need glasses after surgery to help them see. They will need regular eye check-ups to make sure their glasses are right for them. More Surgery: Some kids might need more surgeries or treatments later to fix other eye problems. Other Eye Problems Glaucoma: Kids who have had cataract surgery are more likely to get glaucoma. This is when pressure builds up in the eye and can hurt the optic nerve. Retina Problems: There might be a higher chance of retina problems after surgery, so doctors will need to keep an eye on it. Living with Cataracts Getting Help: With the right treatment, many kids with congenital cataracts can live a normal life. Early treatment and regular check-ups help a lot. Independence: Many children can do most things on their own but may need a little help sometimes. Summary Congenital cataracts can cause long-term problems, but early treatment can make a big difference. Regular eye check-ups help manage any issues and make sure the child has the best vision possible. | 5 | 5 | 5 | 5 | 5 | 5 | 4 | 4 | 5 | 5 | 5 | 5 | 5 | 5 | 5 |
|  | 8 | Does congenital cataract cause amblyopia? | Yes, congenital cataracts can cause amblyopia, which is often called "lazy eye." Amblyopia happens when one eye has weak vision because the brain and the eye are not working together properly. Here’s how congenital cataracts can lead to amblyopia:  How It Happens Cloudy Vision: Congenital cataracts make the lens of the eye cloudy. This cloudiness blocks light from reaching the retina, which is the part of the eye that sends images to the brain. Because of this, the affected eye cannot send clear signals to the brain.  Development of Vision: Babies and young children need good vision to help their brains develop properly. If one eye isn’t sending clear images, the brain might start to favor the other eye. This can stop the affected eye from developing good vision pathways.  Brain Adaptation: The brain might ignore the input from the weaker eye because it is not getting clear information. This causes the brain to rely more on the stronger eye.  Consequences Weaker Vision: The affected eye may have poorer vision compared to the stronger eye, even if the eye looks normal after treatment.  Problems with Using Both Eyes: Amblyopia can make it hard to use both eyes together. This can lead to difficulties with depth perception, which is the ability to judge how far away things are.  Prevention and Treatment Early Treatment: It’s important to find and treat congenital cataracts as soon as possible. Early detection can help prevent or reduce the risk of amblyopia and support normal vision development.  Surgery: Removing the cataract through surgery is often necessary. After surgery, kids may need glasses or other tools to help them see clearly.  Vision Therapy: If amblyopia occurs, extra treatments like vision therapy or patching the stronger eye might be needed. This helps the brain learn to use the weaker eye better.  Regular Check-Ups: Ongoing care is important to keep track of how vision is developing and to address any problems.  Summary In summary, congenital cataracts can cause amblyopia if they are not treated quickly. Early diagnosis and treatment are very important to reduce the risk of amblyopia and to help children develop the best vision possible. | 5 | 5 | 5 | 5 | 5 | 5 | 5 | 5 | 5 | 5 | 5 | 5 | 5 | 5 | 5 |
|  | 9 | What is the minimum age for congenital cataract surgery? | When is Surgery Done? Early Surgery: Doctors usually want to do the surgery when the baby is very young, often within the first few months of life. This helps prevent problems with vision. Timing: Most surgeries are done between 6 to 12 weeks after birth if the cataract is affecting the baby's vision. What Affects When Surgery Happens? How Bad the Cataract Is: If the cataract is large or thick, doctors may want to do the surgery sooner. Vision Problems: If the cataract makes it hard for the baby to see, doctors will want to do the surgery early to help the baby’s eyes develop correctly. Baby’s Health: If the baby has other health issues, surgery may be delayed until those are taken care of. Why is Early Surgery Important? Stops Lazy Eye: Early surgery can help prevent amblyopia, which is when the brain ignores the weak eye. Better Vision: Doing the surgery early usually means better vision as the child grows up. Fewer Future Problems: Early surgery can help avoid serious vision problems later on. What Happens After Surgery? After the surgery, the child will need regular check-ups to make sure their vision is developing well. They might need glasses or other treatments to help them see better.  Summary There isn’t a specific age for congenital cataract surgery, but it’s best to do it as soon as possible, usually in the first few months of life. This helps the baby see better and grow normally. Regular follow-ups are important to keep track of their vision. | 4 | 3 | 5 | 4 | 4 | 5 | 5 | 4 | 5 | 5 | 5 | 5 | 5 | 4 | 5 |
|  | 10 | What happens if congenital cataracts are left untreated? | What Happens if Congenital Cataracts Aren’t Treated? If congenital cataracts are not treated, they can cause big problems for a child’s vision and how they grow. Here’s what might happen:  1. Poor Vision Blocked Light: Cataracts can block light from getting to the back of the eye, which makes it hard to see. If they aren’t treated, this can lead to serious vision problems or even blindness in that eye. 2. Lazy Eye (Amblyopia) Brain Ignoring the Eye: If the cataract stops clear images from reaching the brain, the brain might start to ignore that eye. This can make it hard for the eye to get better vision. 3. Slow Visual Development Hard to See as They Grow: Babies and young children need good vision to develop properly. Untreated cataracts can mess up their ability to see, leading to long-term problems. 4. Crossed Eyes (Strabismus) Eyes Not Working Together: When a child can’t see well, their eyes might not line up properly. This can cause more vision issues and may need extra treatment. 5. Difficulty with Daily Activities Challenges with Everyday Tasks: Vision problems can make it tough for a child to read, write, or play with friends. Why Early Treatment is Important Finding and treating congenital cataracts early is very important. This helps avoid these problems and gives the child the best chance for good vision. Treatment usually means surgery to remove the cataract, and sometimes they may need glasses or therapy to help them see better. | 5 | 5 | 5 | 5 | 4 | 4 | 5 | 4 | 5 | 5 | 5 | 5 | 5 | 5 | 5 |
|  |  | What is the success rate of congenital cataract surgery? | Success of Congenital Cataract Surgery Congenital cataract surgery often works really well, especially if it happens early and is followed by good care. Here are some key points:  1. When the Surgery is Done Early Surgery: The sooner the surgery is done (usually in the first few months of life), the better the results. Early surgery helps prevent problems like lazy eye. 2. Type of Cataract Cataract Size: How big or thick the cataract is can affect the surgery. Bigger or thicker cataracts can be harder to remove. 3. Surgeon’s Skills Experienced Doctors: The skills of the surgeon matter a lot. Doctors who have done many surgeries on babies tend to get better results. 4. Care After Surgery Follow-Up Care: After surgery, it’s important to have regular check-ups and to wear glasses if needed. This helps ensure everything goes well. 5. Child’s Health Overall Health: The child’s general health can also affect how well the surgery goes. If a child has other health issues, it might make the surgery more complicated. Success Rates Vision Improvement: Most kids see a big improvement in their vision after surgery. About 70-80% of children can see well if they get treatment early and have good follow-up care. Possible Issues: Some kids may have problems like increased pressure in the eye or issues with the lens, but many of these can be treated. Long-Term Effects Good Vision Development: With early surgery and regular check-ups, many kids can have good vision as they grow. It’s important to keep checking their eyes over time. Summary In summary, congenital cataract surgery usually has good results, especially when done early and with proper care. Most children see significant improvements in their vision, though outcomes can vary based on the type of cataract and the child’s overall health. | 5 | 5 | 5 | 5 | 5 | 5 | 4 | 4 | 5 | 5 | 5 | 5 | 5 | 5 | 5 |
|  | 12 | What is the most common complication of congenital cataract surgery? | Common Problem After Cataract Surgery: PCO What is PCO? Cloudy Capsule: After cataract surgery, sometimes the back part of the lens capsule gets cloudy. This is called posterior capsule opacification (PCO) or "secondary cataract." Why Do Kids Get PCO More? Fast Healing: Babies and young children are more likely to get PCO because their eyes heal very quickly. If PCO happens and is not treated, it can cause problems with vision. How Do Doctors Fix It? Surgery to Help: To prevent PCO, doctors often remove the cloudy part of the capsule during surgery. This is called a posterior capsulotomy. Extra Care: Sometimes, they also remove some gel-like substance behind the lens. This helps keep vision clear and reduces the chances of PCO. Summary In short, posterior capsule opacification (PCO) is a common issue after cataract surgery in children. Doctors take steps during surgery to prevent it and keep the child’s vision clear. | 4 | 3 | 5 | 3 | 3 | 5 | 5 | 4 | 5 | 4 | 4 | 5 | 4 | 3 | 5 |
|  | 13 | What is the surgery of choice for congenital cataract? | Best Surgery for Congenital Cataracts 1. What is Phacoemulsification? Breaking the Cloudy Lens: This surgery uses sound waves to break the cloudy lens into tiny pieces. Removing the Pieces: The doctor then sucks out these small pieces from the eye. Gentler Method: This way of doing surgery uses smaller cuts, making it easier on the eye. 2. What is an IOL? Putting in a New Lens: After removing the cloudy lens, the doctor puts in a new lens called an intraocular lens (IOL). This helps the child see clearly. Types of IOLs: Monofocal Lenses: These lenses help you see clearly at one distance (like far away). Accommodating Lenses: These lenses help with both near and far vision. Contact Lenses: Sometimes, very young children may use contact lenses instead of an IOL for a while. 3. When is the Surgery Done? Early is Best: The surgery is usually done in the first few months of life. This helps prevent vision problems, like lazy eye. 4. After Surgery Care Check-Ups: After the surgery, regular visits to the doctor are very important to make sure the child’s vision is getting better and the eye is healing. Glasses or Contacts: The child may need glasses or contact lenses to see better after surgery. 5. Benefits of This Surgery Clearer Vision: The main goal of the surgery is to help the child see clearly. Fewer Problems: This surgery is less likely to cause issues compared to older methods. Summary In short, phacoemulsification with IOL implantation is the best surgery for congenital cataracts. It helps children see better and has fewer risks. Doing the surgery early and having good follow-up care are very important for great results. | 4 | 3 | 4 | 4 | 4 | 4 | 4 | 4 | 4 | 4 | 4 | 4 | 4 | 4 | 4 |
|  | 14 | How are congenital cataracts optically corrected? | Congenital cataracts can be treated in several ways to help children see better. Here’s how doctors usually manage and correct these cataracts:  1. Surgery Phacoemulsification and Intraocular Lens (IOL) Implantation  What Happens: After the cataract is removed, the doctor puts in an artificial lens called an intraocular lens (IOL) to replace the cloudy lens. This lens helps focus light correctly onto the retina so the child can see clearly. Types of IOLs: The type of IOL depends on the child’s age and needs. Very young children might get the IOL during the surgery, or they may start with contact lenses until their eyes grow enough for an IOL. 2. Using Glasses and Contact Lenses Glasses  Use: After surgery, children often wear glasses to fix any vision problems that might still be there. Glasses can help with things like nearsightedness (not seeing far away), farsightedness (not seeing up close), or astigmatism (blurry vision). Customization: The glasses will be made to fit the child’s vision needs and any changes that happen after surgery. Contact Lenses  Use: Sometimes, contact lenses are used if the doctor can’t put in an IOL right away, or as a temporary solution before the child’s eyes are ready for an IOL. Contacts can be adjusted easily for better vision. Benefits: Contact lenses can give a wider view and can be more comfortable than glasses. 3. Vision Therapy Rehabilitation  Use: If a child has issues like amblyopia (lazy eye) or trouble using both eyes together, vision therapy or patching may be recommended. This helps improve how the eyes work together and encourages the use of the weaker eye. Techniques: Therapy might include exercises to strengthen eye muscles and improve visual skills. 4. Follow-Up Care Regular Monitoring  Importance: Regular check-ups with an eye doctor are very important. These visits help track how the child’s vision is developing, adjust glasses or contacts, and deal with any new problems that come up. Adjustments: Regular eye exams ensure that glasses or contact lenses are updated as needed to help the child see their best. Summary Treating congenital cataracts includes surgery like phacoemulsification with IOL implantation and using glasses or contact lenses afterward. Vision therapy may also help with extra vision problems. Regular check-ups are crucial to make sure the child gets the best possible care for their vision. | 5 | 4 | 4 | 5 | 5 | 4 | 5 | 4 | 4 | 5 | 5 | 4 | 5 | 5 | 4 |
|  | 15 | Is congenital cataract progressive? | Congenital Cataracts: Important Points 1. Do They Change? Not Usually: Once a congenital cataract forms, it usually stays the same. It doesn’t get worse like cataracts in older people. 2. What Can Happen If Not Treated? Vision Problems: If a congenital cataract is not treated, it can cause other issues: Lazy Eye: The affected eye may not see well, leading to lazy eye. Crossed Eyes: If the eyes don’t work together, one might turn in, causing crossed eyes. 3. After Surgery Cloudy Spot: After surgery, some kids might develop a cloudy area behind the lens called PCO. This can usually be fixed with a quick laser treatment. Vision Help Needed: Some children may still have trouble seeing clearly after surgery. 4. Regular Check-Ups Eye Exams Are Important: Kids need regular eye exams to check their vision and make sure any glasses fit properly. Summary In short, congenital cataracts usually don’t get worse after they form. But if not treated, they can cause vision problems. Early treatment is very important for better vision. Regular check-ups help keep an eye on their vision. | 5 | 4 | 4 | 5 | 3 | 3 | 4 | 3 | 4 | 5 | 3 | 4 | 5 | 4 | 4 |
| **RE-CD** | 1 | Will the vision improve after congenital cataract surgery? | Yes, vision often gets much better after congenital cataract surgery, especially if the cataract was blocking vision. The main goal of the surgery is to take out the cloudy lens and replace it with a clear artificial lens called an intraocular lens (IOL). If an IOL isn’t a good choice, the doctor may suggest using glasses or contact lenses to help improve vision.  Here are some important points to remember:  Early Surgery: The earlier the surgery is done, the better the chances for good vision. Doing the surgery early helps the eyes develop properly and lowers the risk of problems like amblyopia (lazy eye).  Recovery: After the surgery, it may take some time for vision to improve as the brain gets used to seeing differently, especially in very young children. Vision therapy and regular check-ups are important during this time.  Vision Improvement: Many people see a big improvement in their vision after surgery, but how well it works can be different for each person. This can depend on how bad the cataract was, if there are other eye problems, and how well the brain adjusts to the new vision.  Your eye doctor will help you know what to expect and how to support the best vision results after surgery. | 2 | 3 | 5 | 2 | 4 | 5 | 4 | 4 | 5 | 2 | 2 | 5 | 2 | 3 | 5 |
|  | 2 | If an IOL is implanted now, will it need to be replaced when the individual reaches adulthood? | In most cases, once an intraocular lens (IOL) is put in, it doesn’t need to be replaced as a person gets older. IOLs are made to be strong and last a lifetime. However, there are a few exceptions to keep in mind:  Lens Power Changes: If a child’s eye keeps growing a lot after the IOL is put in, the lens might not work as well. This could mean they need to replace or adjust the lens when they are adults, but this is pretty rare.  Complications or Changes: If there are problems, or if the lens moves out of place or causes other issues, it might need to be replaced or fixed.  Advances in Technology: Sometimes, new technology for IOLs might give better options. In these cases, replacing the lens could be considered to improve vision or comfort.  Going to regular follow-up appointments with your eye doctor will help make sure the IOL is still working well for your vision and to check for any issues that might come up. | 5 | 5 | 5 | 5 | 4 | 5 | 5 | 5 | 5 | 5 | 5 | 5 | 5 | 5 | 5 |
|  | 3 | Is the congenital cataract surgery risky? | Congenital cataract surgery is usually safe and works well, but like any surgery, it has some risks and possible problems. Here’s what you need to know:  Risks and Complications:  Infection: There’s a small chance of getting an infection after the surgery, but this is rare because doctors use clean techniques and medicine to prevent it.  Swelling: Some swelling can happen after the surgery, but doctors can treat it with medicine.  Eye Pressure: Sometimes, the pressure inside the eye can go up after surgery. Doctors will check for this and can help if it happens.  Retinal Detachment: There is a very small chance that the retina (the back part of the eye) can come loose, especially in young children. This is rare.  Lens Movement: The artificial lens (IOL) can sometimes move out of place, which might need another surgery to fix it.  Visual Outcomes: Many people see a big improvement in their vision after surgery, but some may still need glasses, contact lenses, or more surgeries to see better.  Benefits and Considerations:  Better Vision: The main benefit is that vision gets much better, especially if the cataract was blocking sight.  Early Surgery: Doing the surgery at a young age can help the eyes grow and reduce the chance of problems like lazy eye.  Regular Check-Ups: Going to the eye doctor for check-ups is very important to make sure everything is okay and that vision stays good.  Overall, congenital cataract surgery is a common procedure that works well most of the time. Talking with your eye doctor about the risks and benefits can help you make a good choice and prepare for recovery. | 4 | 3 | 5 | 4 | 3 | 4 | 5 | 4 | 4 | 5 | 4 | 4 | 5 | 5 | 5 |
|  | 4 | Do we have to proceed with the congenital cataract surgery now, or can we wait? | Deciding whether to have congenital cataract surgery right away or to wait is an important choice. Here’s an overview of things to think about when making this decision:  Reasons to Consider Early Surgery:  Visual Development: It’s really important to have surgery early so the eyes can develop properly. The brain is still learning to see in babies and young children. If a congenital cataract is not treated, it can lead to long-term vision problems, like amblyopia (lazy eye).  Preventing Problems: If cataracts are not treated, they can cause other issues, like strabismus (crossed eyes) or more serious vision problems, which might be harder to fix later.  Better Results: Research shows that having surgery earlier usually leads to better vision later on and may reduce the need for extra treatments.  Factors to Consider Before Deciding to Wait:  Severity of the Cataract: Whether you need surgery right away can depend on how bad the cataract is and how much it is affecting vision. If it isn’t causing big problems, your eye doctor might suggest just watching it for now.  Age and Health: The child’s overall health and how ready they are for surgery matter. Sometimes, the doctor might suggest waiting if they think the child is too young or has other health issues.  Chance of Natural Improvement: In rare cases, some congenital cataracts might improve on their own, but this doesn’t happen very often.  Making the Decision:  Talk with Your Doctor: It’s important to discuss everything with your eye doctor. They can explain what the cataract looks like, the risks of waiting, and the benefits of having surgery early.  Regular Check-Ups: If you decide to wait, regular eye exams are very important to watch for any changes in the cataract and to make sure it’s not hurting the child’s vision.  In the end, the decision will depend on the details of the cataract and what the child needs for their vision. Your eye doctor can give you personalized advice to help you make the best choice for your child’s health and development. | 5 | 5 | 5 | 5 | 5 | 4 | 5 | 4 | 4 | 5 | 4 | 4 | 5 | 5 | 5 |
|  | 5 | Will congenital cataract redevelop after the surgery? | Congenital cataracts usually do not come back after surgery, but there are some important things to know:  Possible Problems After Surgery:  Cloudy Membrane (PCO): After the cataract is removed, the thin layer that holds the new lens can sometimes get cloudy. This is not the cataract coming back, but it can make vision blurry. Doctors can fix this with a quick and simple procedure called YAG laser capsulotomy.  Lens Issues: Rarely, the artificial lens (IOL) can move out of place or have other problems that might affect vision. This isn’t the cataract returning; it’s a different problem that may need treatment.  Other Eye Problems: If there were other eye issues before surgery, those may still need care and checking after the cataract surgery.  Ongoing Check-Ups:  Regular Eye Exams: Even after a successful surgery, it’s important to have regular eye exams to make sure the eye is healthy and the new lens is working well.  Watching Vision Development: For children, regular check-ups are very important to see how their vision is growing and to fix any issues that may come up as they get older.  While the original cataract does not come back, regular eye care is important to keep vision clear and to catch any new problems that may arise. | 5 | 3 | 4 | 5 | 4 | 4 | 5 | 4 | 4 | 5 | 4 | 4 | 5 | 3 | 4 |
|  | 6 | Can we continue with amblyopia training and delay the congenital cataract surgery for a few years? | Amblyopia (lazy eye) training is important for helping vision, but it usually cannot take the place of congenital cataract surgery if the cataract is really blocking sight. Here’s why:  Why Early Surgery is Important:  Helping Vision Development: For babies and young children, it’s very important to avoid vision problems. Cataracts that block sight can stop the brain from learning to see properly. Removing the cataract early helps the eyes develop well and lowers the risk of lazy eye.  Preventing Lazy Eye: If a congenital cataract is not treated, it can cause lazy eye, where one eye becomes weaker than the other. Treatments for lazy eye, like patching or vision therapy, work better when started early, but they are most effective after the cataract is removed.  Risks of Waiting for Surgery:  Worsening Vision: Waiting too long for surgery can make vision worse and make it harder to treat lazy eye. The longer a cataract blocks vision, the harder it is to get good results, even with training for lazy eye.  Other Eye Problems: Untreated cataracts can lead to more issues, like crossed eyes, which can make treatment more difficult later on.  Steps to Take:  Talk to Your Eye Doctor: It’s important to talk with your eye doctor about your child’s cataract and vision needs. They can give you advice based on how serious the cataract is and how it might affect sight.  Combine Treatments: Sometimes, the eye doctor might suggest doing the surgery and continuing lazy eye training to help with any vision issues after the surgery.  In general, early surgery is often suggested to avoid problems from congenital cataracts and to help with good vision development. Your eye doctor can help you understand why early surgery might be the best choice and what other treatments you might consider. | 5 | 5 | 5 | 5 | 5 | 5 | 5 | 5 | 5 | 5 | 5 | 5 | 5 | 5 | 5 |
|  | 7 | Our eldest child has bilateral congenital cataracts, and we are considering having a second child. What is the likelihood of the second child developing cataracts? | The chance of having a second child with congenital cataracts can depend on a few things, like genetics and other factors. Here’s a simple explanation:  Genetic Factors: Inherited Conditions: If your first child has cataracts because of a genetic issue, your second child might also be at higher risk. Some conditions that cause cataracts can be passed from parents to kids. You might want to talk to a genetic counselor to understand this better.  Genetic Counseling: A genetic counselor can help you learn about your family’s genetics. They can tell you how likely it is for a second child to have cataracts and discuss testing options.  Other Factors: Non-Genetic Causes: Sometimes, cataracts happen for reasons that are not genetic. For example, if a mother has an infection during pregnancy (like rubella) or is exposed to certain harmful substances, it can lead to cataracts in the baby. If this was the cause for your first child's cataracts, the risk for a second child might be lower. Still, it’s important to have good prenatal care and avoid known risks.  Individual Risk: Every pregnancy is different. The chance of congenital cataracts in your second child might not be the same as in your first child. Your doctor will check the health of the pregnancy and any issues that may come up.  Next Steps: Talk to Your Doctor: It’s important to discuss your family history and any worries with your doctor or a genetic counselor. They can give you advice based on your situation.  Get Regular Check-Ups: Make sure to have regular doctor visits during your pregnancy to monitor health and catch any potential issues.  In general, if the first child’s cataracts are caused by a genetic condition, there might be a higher risk for the second child. However, every pregnancy is unique. Speaking with a specialist will help you get the best information and make smart choices. | 5 | 5 | 5 | 5 | 5 | 5 | 5 | 5 | 5 | 5 | 5 | 5 | 5 | 5 | 5 |
|  | 8 | After congenital cataract surgery, is it better to wear rigid gas-permeable (RGP) lenses or regular spectacles? | After congenital cataract surgery, choosing between rigid gas-permeable (RGP) lenses and regular glasses depends on a few things. These include what the patient needs, how well they can see with the intraocular lens (IOL), and what feels comfortable for them. Here’s a comparison to help you understand the options:  Rigid Gas-Permeable (RGP) Lenses: Advantages:  Good Vision: RGP lenses can provide great vision, especially if there are issues with the shape of the cornea or if the IOL power isn’t fully corrected with glasses.  Stability: They give stable and precise vision correction, which can help if there is astigmatism.  Durability: RGP lenses are usually strong and don’t scratch easily.  Disadvantages:  Comfort: They can be less comfortable than soft contact lenses or glasses, especially for people who are not used to wearing them.  Getting Used To: Some people may need time to get used to wearing RGP lenses.  Regular Glasses: Advantages:  Easy to Use: Glasses are simple to put on and don’t touch the eye, making them a safe choice.  Comfort: Many people find glasses more comfortable than contact lenses.  Low Maintenance: They are easier to clean and take care of than contact lenses.  Disadvantages:  Vision Limits: Glasses may not fix certain vision problems as well as contact lenses, especially if there are big distortions.  Limited Side Vision: Glasses might not give as wide a field of view as contact lenses.  Things to Think About: Vision Needs: If the child has specific vision needs, like astigmatism that glasses can’t fix, RGP lenses might be a better choice.  Comfort and Lifestyle: Think about what is comfortable for the child and their lifestyle. Young kids might find it easier to wear glasses instead of contact lenses.  Follow-Up Care: Regular check-ups with the eye doctor are important to make sure the chosen option is working well and is comfortable.  Conclusion: Both RGP lenses and glasses have their pros and cons. The choice often depends on the person’s specific vision needs, comfort, and lifestyle. Your eye doctor can give you advice based on how the cataract surgery went and what the child needs for their vision. | 5 | 5 | 5 | 5 | 4 | 4 | 5 | 3 | 4 | 5 | 4 | 4 | 5 | 5 | 5 |
|  | 9 | The child currently has nystagmus. Will the nystagmus resolve after congenital cataract cataract surgery? | Nystagmus is when the eyes move quickly and uncontrollably. After surgery for congenital cataracts, nystagmus might get better, stay the same, or sometimes get worse. Here’s how surgery can affect nystagmus:  Possible Effects of Cataract Surgery on Nystagmus: 1. Improvement:  Better Vision: If the cataracts were blocking vision, taking them out can help the child see clearly. This clearer vision might help reduce nystagmus, especially if it was partly caused by not seeing well. More Stability: When vision gets better, it can help steady how the eyes work, which may make nystagmus less intense. 2. No Change:  Other Causes: If nystagmus is caused by problems in the brain or eyes that aren’t related to the cataracts, the surgery might not change it much. 3. Worsening:  Adjustment Period: After surgery, there might be a short time when nystagmus gets a little worse. This can happen while the eyes and brain get used to seeing better. Management and Follow-Up: Regular Check-Ups: After cataract surgery, it’s important to watch the child’s vision and nystagmus closely. Regular visits to the eye doctor will help see how things are going and make any needed changes.  Extra Treatments: If nystagmus doesn’t get better or causes problems after surgery, other treatments might be needed. This could include vision therapy or special glasses.  Team Approach: Sometimes, a team of doctors, including eye doctors and other specialists, might work together to help manage nystagmus.  Summary: Cataract surgery can help the child see better and might improve nystagmus, but it doesn’t always make it go away. Close monitoring and possible extra treatments may be needed based on how the child responds to surgery. | 5 | 4 | 5 | 5 | 5 | 5 | 5 | 4 | 5 | 5 | 5 | 5 | 5 | 5 | 5 |
|  | 10 | We have unilateral congenital cataract in our child. It's said that the surgical outcomes for unilateral cases are not as good. Is this true? | When it comes to surgery for congenital cataracts in one eye (unilateral), the results can be different than for cataracts in both eyes (bilateral). However, having cataracts in just one eye doesn’t always mean the results will be worse. Here are some factors that can affect how well the surgery works:  Factors That Affect Outcomes: 1. Visual Development:  One Eye vs. Two Eyes: If a child has cataracts in only one eye, that eye may not have seen clearly for some time. This can affect how well the eye develops. If the cataract is removed early, the brain can adjust better, but how long the child couldn’t see clearly can still matter. 2. Amblyopia (Lazy Eye):  Risk of Amblyopia: The eye with the cataract may be at risk for amblyopia because it hasn’t had clear vision. To help with this, it’s important to remove the cataract and also treat amblyopia with methods like patching the stronger eye or doing vision therapy. 3. Surgical Technique and Lens Choice:  Lens Placement: Choosing the right artificial lens (IOL) and placing it correctly is very important. For cases with cataracts in one eye, the focus and alignment of the lens need to be just right to help the child see as well as possible. 4. Rehabilitation:  Post-Surgery Care: After surgery, care such as vision rehabilitation and using glasses or contact lenses can help improve vision. What to Expect After Surgery: 1. Visual Acuity:  With timely and appropriate surgery, many children with cataracts in one eye can see well after the surgery. However, how well they see in the end can vary based on things like how old they were when they had surgery, whether they developed amblyopia, and how well they respond to treatments after surgery. 2. Adaptation:  The brain can adapt well to changes, especially if the surgery is done early. This can help the child see nearly normally, though there may still be a slight difference in vision between the two eyes. Conclusion: Congenital cataracts in one eye can be challenging, especially with visual development and amblyopia. However, many children can get good vision with the right surgery and care afterward. The key is to find the cataract early and get treatment quickly, along with regular check-ups to watch for any new issues. Talking to a pediatric eye doctor can give more information about what to expect for your child. | 5 | 5 | 5 | 5 | 5 | 5 | 5 | 5 | 5 | 5 | 5 | 5 | 5 | 5 | 5 |
|  | 11 | After congenital cataract surgery in a child, is it necessary to regularly monitor intraocular pressure and axial length? | After a child has surgery for congenital cataracts, it’s very important to have regular check-ups to monitor the pressure inside the eye (intraocular pressure or IOP) and the length of the eye (axial length). Here’s why:  Monitoring Intraocular Pressure (IOP): Risk of Glaucoma: After surgery, kids can be at risk for glaucoma, a condition that can damage the eye. This can happen because of inflammation, changes in how the eye is shaped, or certain medications. Checking IOP regularly helps find glaucoma early, so it can be treated right away.  Signs of Complications: If the IOP is too high, it could mean there are problems like inflammation that need to be fixed quickly to avoid long-term issues.  Monitoring Axial Length: Eye Growth: Children’s eyes keep growing and changing even after cataract surgery. Measuring axial length helps track this growth and make sure everything is going well. This is important for adjusting things like glasses or contact lenses.  Lens Power Needs: Changes in how long the eye is can affect how strong the lenses need to be for clear vision. Regular measurements help ensure that the glasses or contact lenses are just right.  Follow-Up Visits: Post-Surgery Check-Ups: Kids need to see the eye doctor often, especially in the first months and years after surgery. The doctor will check IOP, axial length, and how well the child is seeing.  Long-Term Care: As the child gets older, the number of visits may go down, but it’s still important to keep up with regular check-ups. This way, any problems can be spotted and treated quickly.  Conclusion: Regular checks of both intraocular pressure and axial length after cataract surgery are key for keeping a child's eyes healthy. These check-ups help manage possible complications, track how well the child can see, and make sure that any needed changes to glasses or lenses are made. Your eye doctor will help you figure out how often these evaluations should happen based on your child’s needs and the type of surgery they had. | 5 | 5 | 5 | 5 | 5 | 5 | 5 | 5 | 5 | 5 | 5 | 5 | 5 | 5 | 5 |
|  | 12 | How long after congenital cataract surgery should the child avoid physical education classes? | After a child has congenital cataract surgery, the time they need to stay away from physical education (PE) classes and hard activities can change. This depends on things like how healthy the child is, what type of surgery they had, and what the eye doctor says. Here’s a simple guide:  Right After Surgery:  First Few Weeks: Children should avoid intense activities and PE classes for about 4 to 6 weeks after the surgery. This time helps the eye heal well and lowers the risk of problems like moving the lens inside the eye or hurting the eye.  Healing Time: During the first weeks, it’s important to make sure the eye is healing properly. Activities like heavy lifting, running, or jumping should be avoided. Gentle activities can start again slowly based on how well the child is recovering and what the eye doctor advises.  Follow-Up and Getting Back to Activities:  Check-Ups: The child will need to visit the eye doctor regularly to check how well their eye is healing. The doctor will let you know when it’s safe for them to start more active play.  Slow Return: After the first few weeks of healing, the child can slowly start doing physical activities again. The doctor may suggest starting with easier activities and then moving to harder ones if there are no problems.  Safety First: If the child plays sports or does activities where the eyes could get hurt, the doctor might recommend using safety goggles or eye shields.  Things to Think About:  Surgery Details: What happened during the surgery, like if there were any problems or extra procedures, can change how long a child should wait before doing physical activities.  Child’s Health: The child’s overall health, how comfortable they feel, and how well they are healing are also important when deciding when to go back to PE classes.  Conclusion:  While a good rule is to avoid tough physical activities for about 4 to 6 weeks after congenital cataract surgery, the best plan is to follow what the eye doctor says. They will give advice based on how the child is healing and the results of the surgery. | 5 | 4 | 5 | 5 | 5 | 5 | 5 | 5 | 5 | 5 | 5 | 5 | 5 | 5 | 5 |
|  | 13 | Can a child have a multifocal intraocular lens implanted during congenital cataract surgery, or is a monofocal lens the only option? | When kids have cataract surgery, doctors usually choose a monofocal intraocular lens (IOL) instead of a multifocal IOL. Here’s why:  Monofocal IOLs: Easy and Safe: Monofocal IOLs are the most common choice for kids. They help kids see clearly either far away or up close, but not both at the same time. They are simple to use and are very safe, which is important for children.  Predictable Results: Monofocal lenses give more reliable results, and doctors can easily make adjustments after surgery if needed. This predictability is helpful as children's eyesight changes and develops.  Growing Eyes: Young kids are still learning to see well. A monofocal IOL can help them see things that are far away, and they can use glasses for things that are close. This makes it easier for them as they grow.  Multifocal IOLs: More Complicated: Multifocal IOLs have different focuses for seeing things that are near, far, and in between. They are more complex and can sometimes cause problems like blurry vision, which might be hard for kids.  Need Time to Adjust: Kids need time to get used to multifocal lenses. These lenses are usually better for adults or older kids whose eyesight is more stable.  Not Common for Kids: Doctors don’t often use multifocal IOLs for children because they can cause more issues. Most doctors prefer monofocal lenses because they are simpler and more reliable.  Important Things to Think About: Age Matters: For very young kids, monofocal lenses are usually the best choice. For older kids, doctors might talk about multifocal lenses, but these are still not common.  Talk to the Doctor: If you’re thinking about multifocal lenses, it’s important to talk to a pediatric eye doctor who understands the best options for kids. They can help decide what is best for your child's needs.  Conclusion: Most of the time, a monofocal IOL is the best choice for cataract surgery in kids because it is safe and gives good results. Multifocal IOLs are usually for adults or older kids. It’s a good idea to talk to a pediatric eye doctor to find the best lens for your child. | 5 | 4 | 5 | 5 | 4 | 4 | 5 | 4 | 4 | 5 | 5 | 4 | 5 | 5 | 5 |
|  | 14 | The severity of congenital cataracts in both of children's eyes is not consistent, should we do cataract surgery in both eyes, or do the severe eye first? | When kids have cataracts (cloudy lenses) in both eyes, doctors think carefully about how to do surgery. Here’s a simple way they usually go about it:  1. Fix the Eye with the Worse Cataract First Worse Vision: If one eye has a cataract that makes it very hard to see, that eye usually gets surgery first. This helps fix the biggest problem right away.  Better Eye Development: Doing surgery on the worse eye helps the child’s vision develop better and can stop problems like lazy eye (amblyopia).  2. Check the Other Eye Watch and Wait: After fixing the worse eye, the doctor will keep checking the other eye. They will watch to see if it gets worse and if it needs surgery later.  Timing: When to do surgery on the second eye depends on how well the child’s vision is improving and if that eye starts having problems.  3. Things to Think About How Bad is the Vision? The more the cataract affects vision, the more important it is to do surgery.  Age of the Child: How old the child is and how well their vision is developing matter. Early surgery on the worse eye helps them see better as they grow.  Surgery Risks: Every surgery has some risks, so it’s important to think about them. Fixing the eye with the worse cataract first helps with planning for recovery.  Check-Ups: After surgery, the child needs to come back for check-ups to see how well the surgery worked and decide when to look at the other eye.  4. Talk to the Eye Doctor Consult a Pediatric Eye Doctor: It’s important to talk to a doctor who knows about kids’ eyes. They can help figure out the best plan for your child.  Personal Plan: The decision to operate on one eye or both eyes will depend on what is best for your child.  Conclusion Most of the time, the eye with the worse cataract is fixed first to help with serious vision problems. The other eye will be watched closely for any needed treatment later. This step-by-step plan helps kids see better. Your eye doctor will give you the best advice for your child’s situation. | 5 | 4 | 5 | 5 | 5 | 5 | 5 | 5 | 5 | 5 | 4 | 5 | 5 | 5 | 5 |
|  | 15 | The congenital cataract surgery has been completed, and now amblyopia training is required. However, all amblyopia training programs increase the duration and intensity of eye use, which could lead to a worsening of myopia. Is this a concern? | Amblyopia training, also called vision therapy or patching, is an important part of helping kids with lazy eye (amblyopia) after cataract surgery. Some parents worry that using their eyes more during this training might make nearsightedness (myopia) worse, but there are ways to handle this. Here’s what you need to know:  1. Why Amblyopia Training is Important: Helping Vision: The main goal of amblyopia training is to make the weaker eye stronger and help the child see better. This training includes fun activities that help the child focus and use their eyes properly. 2. Watching for Myopia: Keeping an Eye on Changes: Using the eyes more can sometimes lead to myopia, but the good things from treating amblyopia are usually worth it. Regular check-ups with the eye doctor will help keep track of any vision changes.  Adjusting Treatment: If the doctor sees that myopia is getting worse, they might change the child’s glasses or suggest special lenses to help control it.  3. Balanced Training: Organized Programs: A good amblyopia training program makes sure the child is using their eyes the right amount. This helps avoid overusing their eyes while still helping them see better.  Personalized Plans: The training should be just right for each child. The eye doctor will change the plan as needed to make sure the child doesn’t strain their eyes too much.  4. Regular Check-Ups: Eye Exams: Going to the eye doctor regularly is very important. These visits help check how well the amblyopia treatment is working and watch for any myopia. If needed, the doctor can change the treatment plan. 5. Extra Help for Myopia: Controlling Myopia: If myopia becomes a problem, the doctor might suggest other options, like special glasses or eye drops, to help. Conclusion: Even though some parents worry that using the eyes more during amblyopia training could make myopia worse, doctors can usually manage this risk. The benefits of helping the child’s vision are usually greater than the risks. Regular check-ups with the eye doctor help make sure the treatment is working well for your child. | 5 | 5 | 5 | 4 | 5 | 5 | 5 | 4 | 5 | 5 | 5 | 5 | 5 | 5 | 5 |
